# Construction of Modifiable Phthalocyanine-Based Covalent Organic Frameworks with Irreversible Linking for Efficient Photocatalytic CO_2_ Reduction

**DOI:** 10.1007/s40820-025-01967-y

**Published:** 2026-01-15

**Authors:** Xuefei Zhou, Shaowei Yang, Zhengyang Hu, Zhanwei Chen, Ying Guo, Tianshuai Wang, Qiuyu Zhang, Hepeng Zhang

**Affiliations:** Xi’an Key Laboratory of Functional Organic Porous Materials, School of Chemistry and Chemical Engineering, Northwestern Polytechnical University, Xi’an, 710129 People’s Republic of China

**Keywords:** Photocatalytic CO_2_RR, Phthalocyanine-based COF, Irreversible covalent bond, Electronic property modulation, Photoelectron transfer

## Abstract

Covalent organic frameworks (COFs) are considered promising catalysts for photocatalytic CO_2_ reduction reaction (pCO_2_RR) due to facilitated regulations. However, the instability of COFs with dynamic reversible covalent bonds and the limited modifiability of COFs with irreversible covalent bonds restricted the enhancement of the pCO_2_RR performance. Herein, three phthalocyanine-based COFs with ether-linked, CoOP, CoPOP, and CoBOP, were successfully prepared via in situ polycondensation using modifiable bis-phthalonitrile. CoBOP achieved a record of syngas performance in pCO_2_RR systems with photosensitizers and sacrificial agents (CO 83.7 mmol g^−1^ h^−1^ and H_2_ 54.7 mmol g^−1^ h^−1^), surpassing most COF photocatalysts. Additionally, CoOP, CoPOP, and CoBOP exhibit stabilities in extreme environments owing to their irreversible covalent bonds. Experimental and density functional theory analyses confirm that the optimally matched the lowest unoccupied molecular orbital of the linking unit between the photosensitizer and active unit endowed CoBOP with the highest photoelectron transfer efficiency among the three catalysts, boosting its pCO_2_RR activity. This work is highly instructive for designing COFs with structure-adjustable and irreversible covalent bonds.

**Supplementary Information:**

The online version contains supplementary material available at 10.1007/s40820-025-01967-y.

## Highlights

- Phthalocyanine-based covalent organic frameworks photocatalysts (CoOP, CoPOP, and CoBOP) with irreversible covalent linking were synthesized by designing bis-phthalonitrile precursors, exhibiting exceptional stability in thermal, acidic, alkaline, and organic environments.
- Tuning the conjugation length of the linking unit effectively modulates the electronic features of the photocatalyst.
- The linking unit serves as a ‘ladder’ between excited [Ru(bpy)_3_]Cl_2_ and Co^2+^, allowing the electrons to cascade down and facilitating rapid transfer, which is responsible for the excellent photocatalytic CO_2_ reduction reaction performance of the photocatalysts.

**Supplementary Information:**

The online version contains supplementary material available at 10.1007/s40820-025-01967-y.

## Graphical abstract

**Figure:**
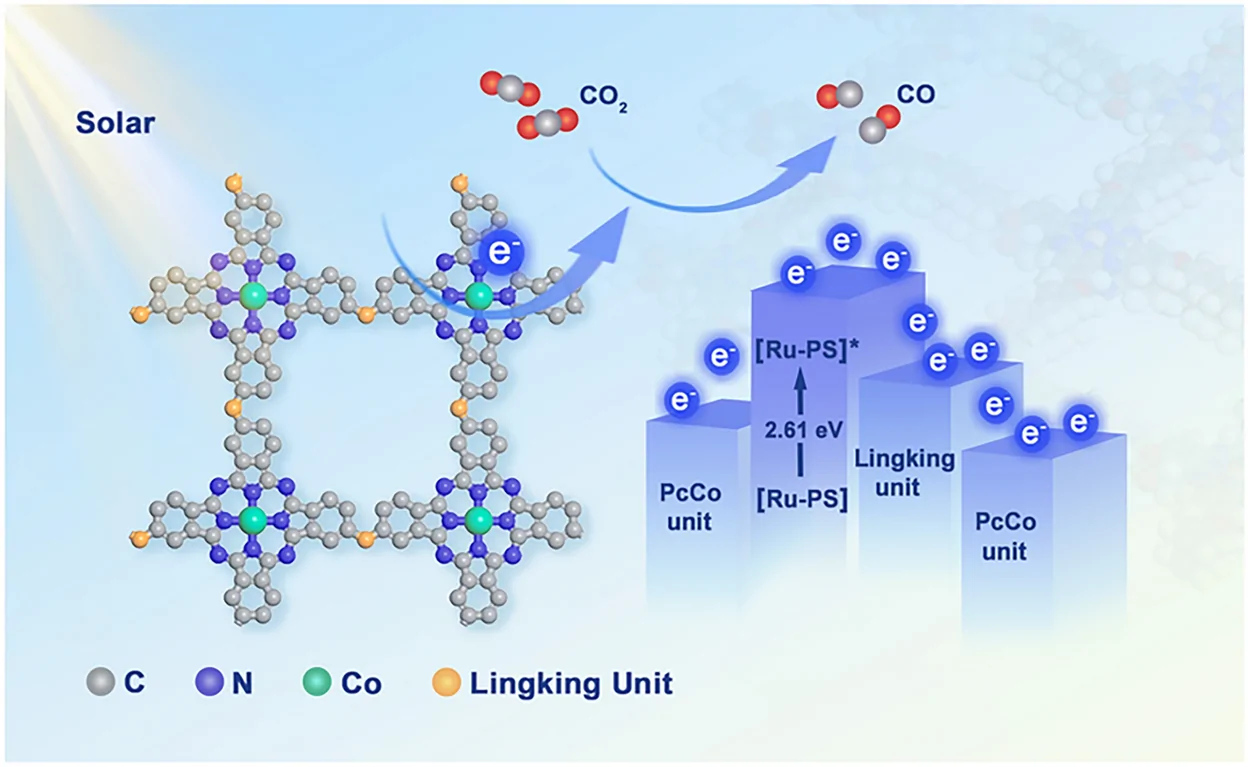

## Introduction

Excessive CO_2_ emissions from fossil fuel consumption and human activities are fueling environmental crises, including global climate change and sea level rise, which threaten sustainable development [1–4]. Consequently, converting CO_2_ into high-value chemicals and fuels, such as syngas, a crucial feedstock for producing energy fuels, particularly in Fischer–Tropsch synthesis, evokes extensive attention and stimulating intensity [5, 6]. Among CO_2_ conversion strategies, photocatalytic CO_2_ reduction reaction (pCO_2_RR) offers a highly promising approach by directly harnessing solar energy [7, 8]. However, the inferior CO_2_ conversion efficiency compared with electrocatalytic and thermocatalytic CO_2_ reduction reactions inhibits the development of pCO_2_RR [9–12]. To solve this intractable issue, many significant efforts have been devoted to the detection of efficient photocatalysts, such as inorganic semiconductors [13], heterostructure materials [14], and metal–organic frameworks [15]. Although substantial progress has been achieved, the development of photocatalysts with poor charge recombination, wide light harvest range, and high energy efficiency is still a significant and challenging task [16].

As the core of pCO_2_RR, the electronic properties of photocatalysts, such as energy band gap (E_g_) and conduction band (CB) position, are crucial to their catalytic performance. The solar absorption capacity of a photocatalyst correlates with its E_g_, while the CB position is key to enabling reduction reactions and also impacts electron transfer from photosensitizer to photocatalyst [17, 18]. Thus, the ability to conveniently adjust these characteristics is essential for highly efficient photocatalysts. Covalent organic frameworks (COFs) are characterized by their structural designability and π-conjugated networks [19–22], making them particularly well-suited to modulate the E_g_ and CB. Specifically, the molecular structures of linker units and catalytic centers in COFs can be precisely tuned, greatly enhancing their photocatalytic performance [23–26], all of which make COFs a promising catalyst for pCO_2_RR. Phthalocyanine (Pc), an 18-electron π-conjugation molecule similar to porphyrin [27], possesses exceptional chemical and thermal durability and stable coordination capacity with metal ions, making it a popular candidate for COF building blocks [28, 29]. Many Pc-based COFs have been developed for use in pCO_2_RR and exhibited excellent photocatalytic performance [30, 31]. Unfortunately, most reported Pc-based COFs are synthesized with dynamic reversible covalent bonds, such as imine and borate bonds, which lowers their stability, especially in acidic, alkaline, and organic solvent circumstances, restricting their further applications.

Recently, COFs with irreversible covalent bonds have stood out for their excellent stability in extremely acidic and basic conditions [32–34]. Inspired by the in situ polycondensation or ionothermal synthesis of tetraniliprole aromatic compounds with metal ions, Pc-based COFs with irreversible bond covalent linking are also obtained, ensuring their remarkable thermal stability and resistance to acids and bases [35–37]. However, the limited modifiability of the linking unit restricts the characteristic of facile structural designability of COFs, thereby preventing the further enhancement of their catalytic performance. A possible solution involves the design and synthesis of tunable bis-phthalonitrile with stable chemical bonds, and then in situ polycondensation of them to form Pc-based COFs with irreversible covalent bonds. This might improve the modifiability of Pc-based COFs by changing the molecular structure of bis-phthalonitrile while ensuring its excellent stability. However, it remains undeveloped to date.

**Fig. 1:**
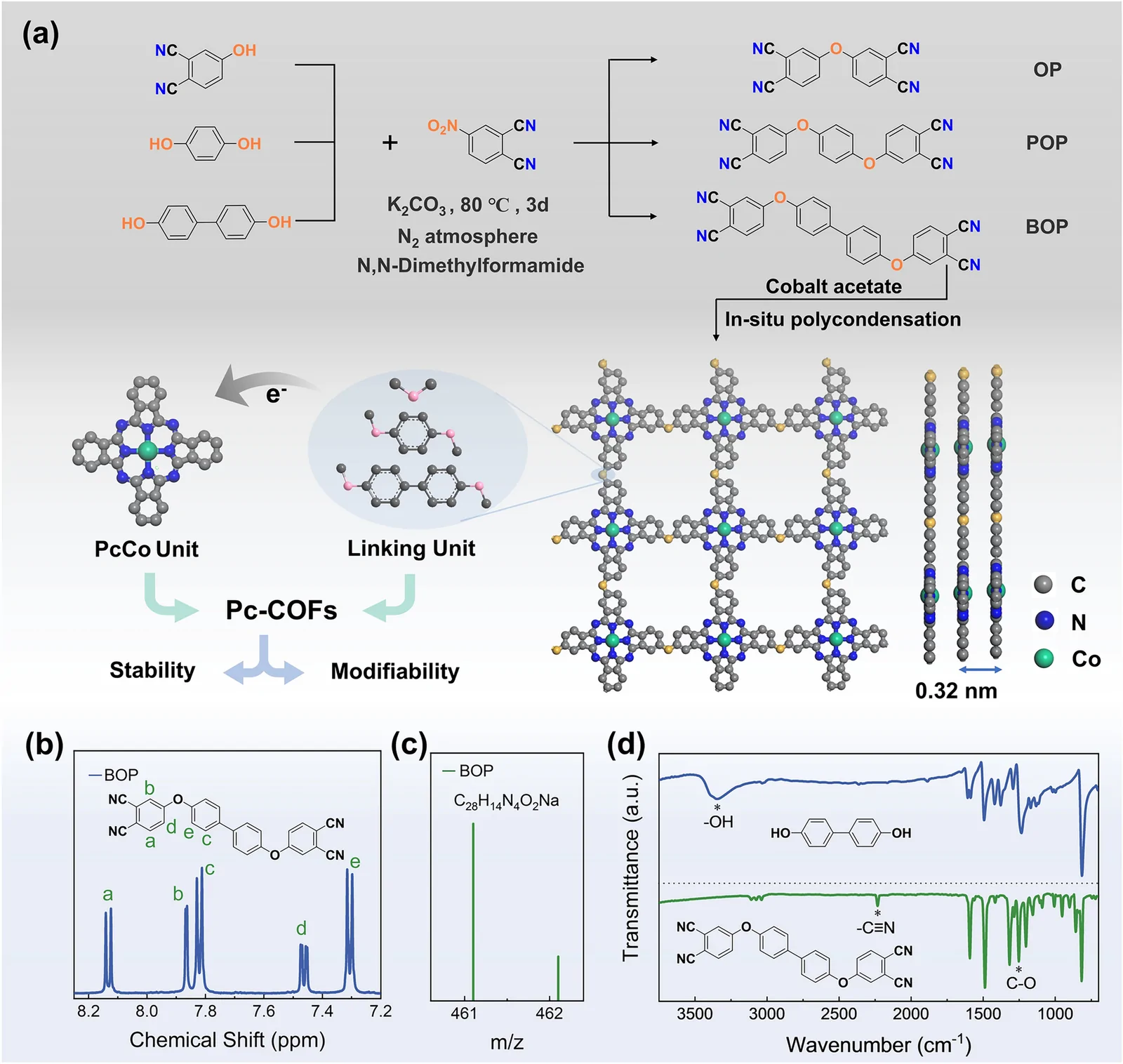
**a** Schematic synthesis of CoOP, CoPOP, and CoBOP. **b** ^1^H NMR spectra of BOP. **c** HR-MS spectra of BOP. **d** FT-IR spectra of 4,4'-dihydroxydiphenyl and BOP

In this work, one strategy for preparing Pc-based COF photocatalysts with tunable irreversible bond covalent linking by bis-phthalonitrile precursors was proposed. Benefiting from the modifiability of bis-phthalonitrile precursors, three stable ether bond-linked Pc-based COFs with different linking unit lengths, denoted as CoOP, CoPOP, and CoBOP, were obtained (Fig. 1a). As expected, CoOP, CoPOP, and CoBOP displayed excellent stabilities in acidic, alkaline, and organic solvents due to the presence of irreversible bonds in the linking units. More importantly, the conjugation length of the linking units could effectively modulate the electronic properties of the three Pc-based COF photocatalysts, which favored the establishment of an efficient electron transport channel between the photosensitizer and the catalytic active site. Taking advantage of the optimally matched lowest unoccupied molecular orbital (LUMO) position of the BOP linking unit between the excited photosensitizer and active Co^2+^ unit, CoBOP demonstrated impressive photocatalytic performance. It successfully reduced CO_2_ to syngas in the presence of photosensitizers and sacrificial agents, achieving CO and H_2_ generation rates of 83.7 and 54.7 mmol g^−1^ h^−1^, respectively. This surpasses most of the reported COF-based catalysts in pCO_2_RR systems with photosensitizers and sacrificial agents (Table S1). In addition, CoBOP has excellent photocatalytic CO_2_ to syngas in a photosensitizer-free system (CO 8.2 mmol g^−1^ h^−1^, H_2_ 4.0 mmol g^−1^ h^−1^).

## Experimental Section

### Photocatalyst Preparation

#### Synthesis of Precursors (OP, POP, and BOP)

4-nitrophthalonitrile (2 mmol), along with 4-hydroxyphthalonitrile (2 mmol), were meticulously introduced into the three-necked flask (100 mL) with 50 mL N,N-dimethylformamide (DMF) and 4 g K_2_CO_3_. The reaction was heated to 80 °C for 3 days under N_2_ atmosphere. After cooling to room temperature, transfer the reaction solution to a 250 mL beaker and add 150 mL of ultrapure water. After standing, filtering, washing with ultrapure water and ethanol, and drying in a vacuum oven at 65 °C overnight, a yellow powder sample (OP) can be obtained. POP and BOP synthesis followed the identical protocol employed for OP, except that 4-hydroxyphthalonitrile (2 mmol) was replaced with hydroquinone (1 mmol) and 4,4-dihydroxydiphenyl (1 mmol), respectively.

#### Synthesis of Photocatalyst (CoOP, CoPOP, and CoBOP)

OP (200 mg) and an excess of Co(OAC)_2_·4H_2_O were added to a three-necked flask with 50 mL of n-pentanol and heated to 140 °C under an N_2_ atmosphere. The reaction was carried out for 3 days, and 0.1 mL of DBU was added to the reaction system every 12 h using a syringe. After cooling to room temperature, the reaction solution was transferred to a 250 mL beaker, and 150 mL of ethanol was added. After filtration and washing with DMF, ethanol, and ultrapure water sequentially several times, the green grass solid powder (CoOP) was collected after drying under vacuum at 70 °C overnight. CoPOP and CoBOP synthesis followed the identical protocol employed for CoOP, except that OP was replaced with POP and BOP, respectively.

### CO_2_ Photocatalytic Reduction

Photocatalytic experiments were performed in a 180 mL quartz glass reactor at 27 °C under a 300 W Xe lamp (AM1.5G, 300 mW cm^−2^). The quartz reactor was used to disperse 1 mg of catalysts and 20 mg [Ru(bpy)_3_]Cl_2_ within a solution containing 12 mL MeCN, 4 mL deionized water, and 4 mL TEOA. The reaction device was closed after CO_2_ blowing for 30 min, and the photocatalytic reaction was carried out for 1 h. The production of CO and H_2_ was monitored using a gas chromatography (FULI INSTRUMENTS GC9720Plus) instrument. In addition, ^1^H NMR spectroscopy was used to detect the possible liquid products.

## Results and Discussion

### Design, Synthesis, and Structural Characterization of CoOP, CoPOP, and CoBOP

The stable Pc-based COFs were prepared by a two-step strategy (Fig. 1a): (1) the synthesis of three ether bond connected bis-phthalonitrile precursors, 4,4'-oxydiphthalonitrile (OP), 4,4'-(1,4-phenylenebis(oxy)) diphthalonitrile (POP), and 4,4'-([1,1'-biphenyl]-4,4'-diylbis (oxy)) diphthalonitrile (BOP), through the aromatic nucleophilic substitution reaction; (2) the preparation of cobalt-coordinated Pc-based COF photocatalysts by the in situ polycondensation of the three obtained precursors and cobalt acetate in n-pentanol solution. The successful synthesis of BOP precursor is supported by ^1^H NMR (Fig. 1b) [(500 MHz, DMSO-d6) δ 8.13 (d, J = 8.7 Hz, 2H), 7.86 (d, J = 2.6 Hz, 2H), 7.82 (d, J = 8.7 Hz, 4H), 7.46 (dd, J = 8.7, 2.6 Hz, 2H), 7.31 (d, J = 8.7 Hz, 4H)] and high resolution mass spectrometry (HR-MS) (m/z = 461.1, Fig. 1c). Moreover, fourier transform infrared spectroscopy (FT-IR) further confirms the successful synthesis of BOP precursor by the disappearance of -OH stretching vibration at 3350 cm^−1^ and the observations of the stretching vibration of -C≡N and C–O–C at 2235 and 1250 cm^−1^, respectively (Fig. 1d). The ^1^H NMR, HR-MS, and FT-IR spectra of OP and POP reach the same conclusion (Figs. S1-S6). FT-IR of CoBOP (Fig. S7) confirms the full conversion of BOP precursor by the disappearance of the -C≡N stretching vibration at 2235 cm^−1^ and the appearance of the stretching vibration of -C = N and -C-N in the phthalocyanine ring at 1473 and 1235 cm^−1^, respectively. Furthermore, the observation of the IR peak of phthalocyanine ring coupled Co–N bonds at 830 cm^−1^ demonstrates the successful coordination between Co and pyrrolic N [34, 38]. The FT-IR spectra of CoOP and CoPOP reach the same conclusions (Figs. S8 and S9).

**Fig. 2:**
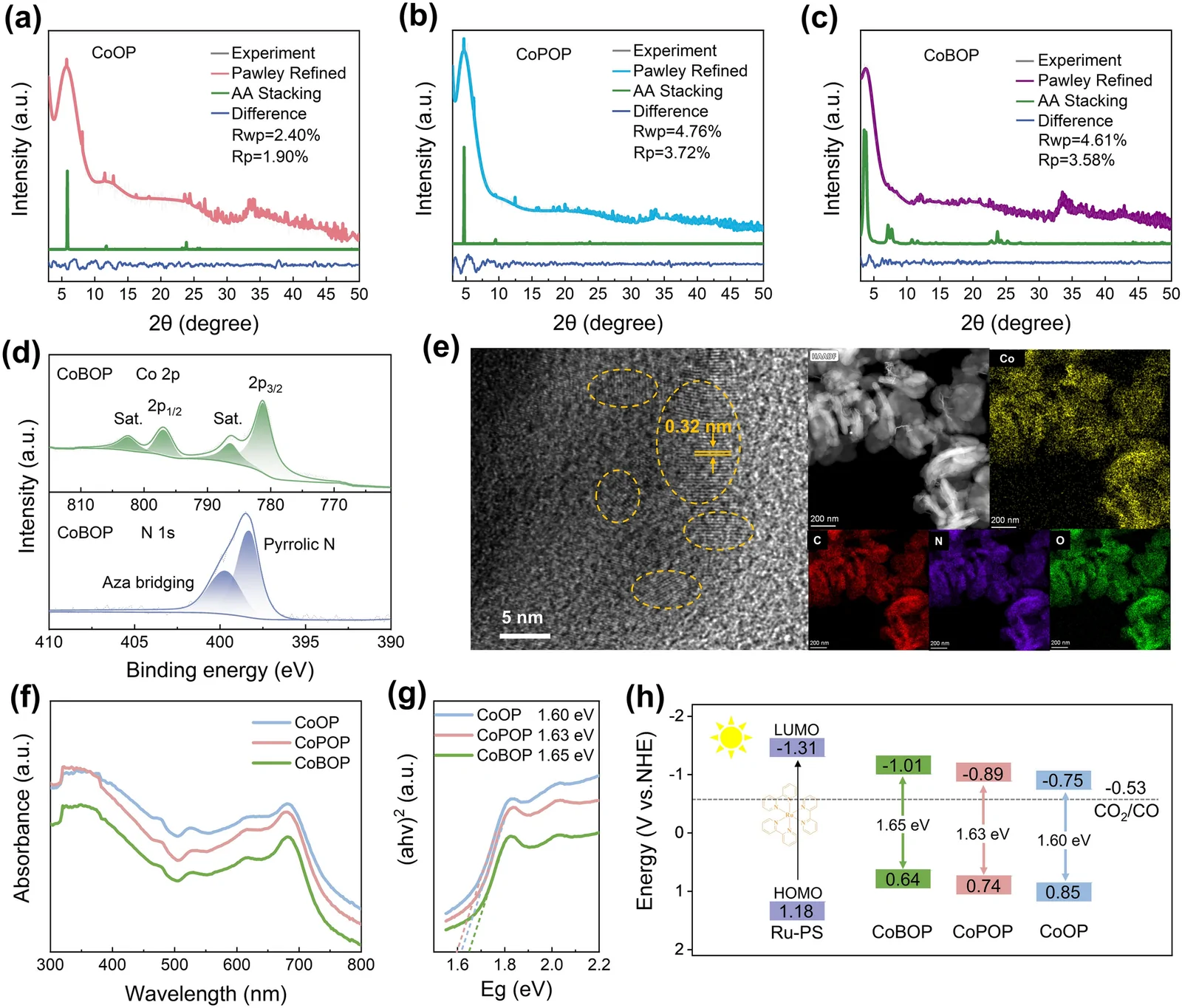
Experiment, Pawley Refined, and AA Stacking simulated PXRD crystal structure based on theoretical structural simulation using the Materials Studio package of CoOP (**a**), CoPOP (**b**), and CoBOP (**c**). **d** Co 2*p* and N 1s XPS spectra of the CoBOP. **e** TEM image and EDS mapping pictures of CoBOP. **f** UV–vis DRS of CoOP, CoPOP, and CoBOP. **g** Kubelka–Munk-transformed reflectance spectra of CoOP, CoPOP, and CoBOP. **h** Band structure (vs NHE) diagram based on UV–vis spectra and Mott-Schottky plots for CoOP, CoPOP, and CoBOP

The crystal structures of CoOP, CoPOP, and CoBOP were determined through powder X-ray diffraction (PXRD) combined with a theoretical structural simulation using the Materials Studio package [39]. Intense diffraction peaks from (100) facets of CoOP, CoPOP, and CoBOP are at 5.70°, 4.78°, and 3.75°, respectively (Fig. 2a–c), agreeing well with the results of the PXRD patterns simulated using the AA stacking mode. Moreover, Pawley refinements based on the simulated structures also match well with the experiment results (*R*_p_ = 1.90% and *R*_wp_ = 2.40% for CoOP, *R*_p_ = 3.72% and *R*_wp_ = 4.76% for CoPOP, and *R*_p_ = 3.58% and *R*_wp_ = 4.61% for CoBOP), indicating the successful preparation of the Pc-based COFs (Tables S2–S4) [40]. Additionally, the three catalysts exhibit minor diffraction peaks near 24° and 34°. To elucidate the origin of these reflections, the PXRD patterns were analyzed using an AB-stacking model (Fig. S10). The fitting results suggest that these small peaks may arise from the presence of trace AB stacking within the catalysts. The absence of dynamic bond exchange during synthesis for the three COFs can limit defect correction, resulting in non-ideal stacking arrangements.

X-ray photoelectron spectroscopy (XPS) further verifies the successful preparation of CoOP, CoPOP, and CoBOP. The Co 2*p* XPS spectrum of CoBOP shows the characteristic Co 2*p*_3/2_ and Co 2*p*_1/2_ at around 781.3 and 797.0 eV, respectively [41–43]. The N 1 s XPS spectrum of CoBOP illustrates the characteristic bridging aza-nitrogens and pyrrolic N at around 400.0 and 398.0 eV, respectively (Fig. 2d). The C 1 s XPS spectrum of CoBOP reveals a -C=C characteristic peak at 284.6 eV and -C-N characteristic peak at 286.2 eV, and the O 1s XPS spectra of CoBOP present -C-O-C characteristic peak at 531.5 eV (Figs. S11 and S12) [35]. All of these undoubtedly induce the formation of the phthalocyanine ring and the existence of ether bonds in the CoBOP photocatalyst. The similar XPS spectra of CoOP and CoPOP also give the same consequences (Figs. S13–S16).

The scanning electron microscopy (SEM) reveals that CoOP, CoPOP, and CoBOP all exhibit irregular morphologies with sizes ranging from tens to hundreds of nanometers (Figs. S17–S19). Additionally, the transmission electron microscopy (TEM) image of CoBOP displays lattice fringes with a spacing of 0.32 nm for π-π stacking. Energy-dispersive X-ray spectroscopy (EDS) images show the uniform distributions of C, N, O, and Co elements in the CoBOP (Fig. 2e). TEM and EDS images of CoOP and CoPOP are similar to the observations of CoBOP (Figs. S20 and S21). The N_2_ sorption isotherms measured at 77 K indicate that the Brunauer–Emmett–Teller (BET) surface areas of CoOP, CoPOP, and CoBOP are 44.9, 55.1, and 30.5 m^2^ g^−1^, respectively. To understand the relatively low BET surface areas of the COFs, AA/AB stacking simulations were performed in conjunction with XRD analysis. The results reveal the presence of non-ideal stacking. N_2_ sorption isotherms analysis indicates the peak pore sizes are 3.8, 3.9, and 3.9 nm for CoOP, CoPOP, and CoBOP, respectively, which differ from the theoretical pore sizes predicted from the simulations (1.4, 1.9, and 2.2 nm), suggesting the existence of pore collapse. These findings collectively indicate that the presence of non-ideal stacking and pore collapse accounts for the relatively low BET surface areas (Fig. S22) [44]. Additionally, to evaluate CO_2_ affinity, the adsorption behavior of these COFs was measured at 1 bar. At 273 K, the CO_2_ uptake capacities are 11.2 cm^3^ g^−1^ for CoOP, 11.3 cm^3^ g^−1^ for CoPOP, and 10.7 cm^3^ g^−1^ for CoBOP. Increasing the temperature to 298 K reduces the uptake to 7.8, 6.6, and 7.5 cm^3^ g^−1^, respectively, consistent with the expected decrease in physisorption at elevated temperatures (Figs. S23–S25) [45]. These results demonstrate that the three COFs maintain moderate CO_2_ adsorption under conditions relevant to the photocatalytic operation. The isosteric heat of adsorption (Qₛₜ) values are calculated from the isotherms obtained at the two temperatures, yielding 25.4 kJ mol⁻^1^ for CoOP, 35.4 kJ mol⁻^1^ for CoPOP, and 27.1 kJ mol⁻^1^ for CoBOP (Figs. S26–S28) [46]. With increasing CO_2_ uptake, the Q_st_ values gradually decrease but remain essentially stable within a certain range, suggesting the relatively uniform distribution of active sites for the three COFs catalysts. CoPOP exhibits the highest Q_st_, yet weaker pCO_2_RR performance compared to CoBOP, implying that CO_2_ adsorption is not the dominant factor governing performance differences.

### Thermal and Chemical Structure Stabilities of CoOP, CoPOP, and CoBOP

As mentioned above, one of the purposes of designing the ether-linked COFs is to improve their stabilities; therefore, the durability of CoOP, CoPOP, and CoBOP under thermal, acidic, alkaline, and organic solvent conditions were all tested. The thermogravimetric analyses confirm the good thermal stabilities of CoOP, CoPOP, and CoBOP, with a weight loss of < 15% at temperatures up to 400 °C under the N_2_ atmosphere (Fig. S29). Moreover, after being treated for 48 h with extremely acidic and basic conditions in 3 M HCl and 3 M KOH, respectively, as well as acetonitrile, the crystallinities of CoOP, CoPOP, and CoBOP are well maintained (Figs. S30–S32). Which endows them with the ability to maintain catalytic activity under harsh conditions [47, 48].

### Band Structure and Spectral Characterization of CoOP, CoPOP, and CoBOP

Another purpose of structuring CoOP, CoPOP, and CoBOP is to regulate their intrinsic optical features. Diffuse reflectance ultraviolet–visible spectra (UV–Vis DRS) exhibit that all three photocatalysts embrace outstanding light absorption properties with a spectral response of 300–800 nm (Fig. 2f). The absorption peak located near 680 nm is the characteristic peak of phthalocyanine [49]. Utilizing the Kubelka–Munk (KM) method [50], the E_g_ determined via the Tauc plots are 1.60, 1.63, and 1.65 eV for CoOP, CoPOP, and CoBOP, respectively (Fig. 2g). Subsequently, Mott–Schottky curve measurements at different frequencies [46] were further accomplished to learn the CB position of CoOP, CoPOP, and CoBOP, and the results show that the CB of CoOP, CoPOP, and CoBOP are found to be − 0.95, − 1.09, and − 1.21 eV vs. Ag/AgCl (Figs. S33–S35), corresponding to − 0.75, − 0.89, and − 1.01 eV vs. the normal hydrogen electrode (NHE, pH = 7). The CB positions become more negative as the conjugation length increases of the linking unit. The band structure diagrams (vs. NHE, pH = 7) for CoOP, CoPOP, CoBOP, and [Ru(bpy)_3_]Cl_2_ show that all the CB of CoOP, CoPOP, and CoBOP are more positive than that of [Ru(bpy)_3_]Cl_2_ (Fig. 2h), ensuring the easy and efficient photogenerated electrons transfer of the photosensitizer to the photocatalysts during the pCO_2_RR process [51]. Meanwhile, the CB positions of CoOP, CoPOP, and CoBOP are all more negative than the reduction potential of CO_2_/CO (− 0.53 eV vs NHE, pH = 7), which favors the reduction of CO_2_ to CO [52]. Furthermore, XPS band structure testing was performed. The Band structure (vs vacuum) of CoOP, CoPOP, and CoBOP based on UV–vis spectra and XPS-VB exhibited a consistent trend (Fig. S36) [53]. The density functional theory (DFT) calculations disclose that the lowest unoccupied molecular orbital (LUMO) of CoOP, CoPOP, and CoBOP are extended into the linking π-conjugated units (Fig. S37), meaning that the change of linking π-conjugated units influences the LUMO distributions of the photocatalysts. Based on the experimental characterizations and theoretical calculations, one important conclusion can be seen that the changes in conjugation length of the linking unit can indeed effectively regulate the intrinsic features of the photocatalyst.

### Photocatalytic CO_2_RR Performance of CoOP, CoPOP, and CoBOP

**Fig. 3:**
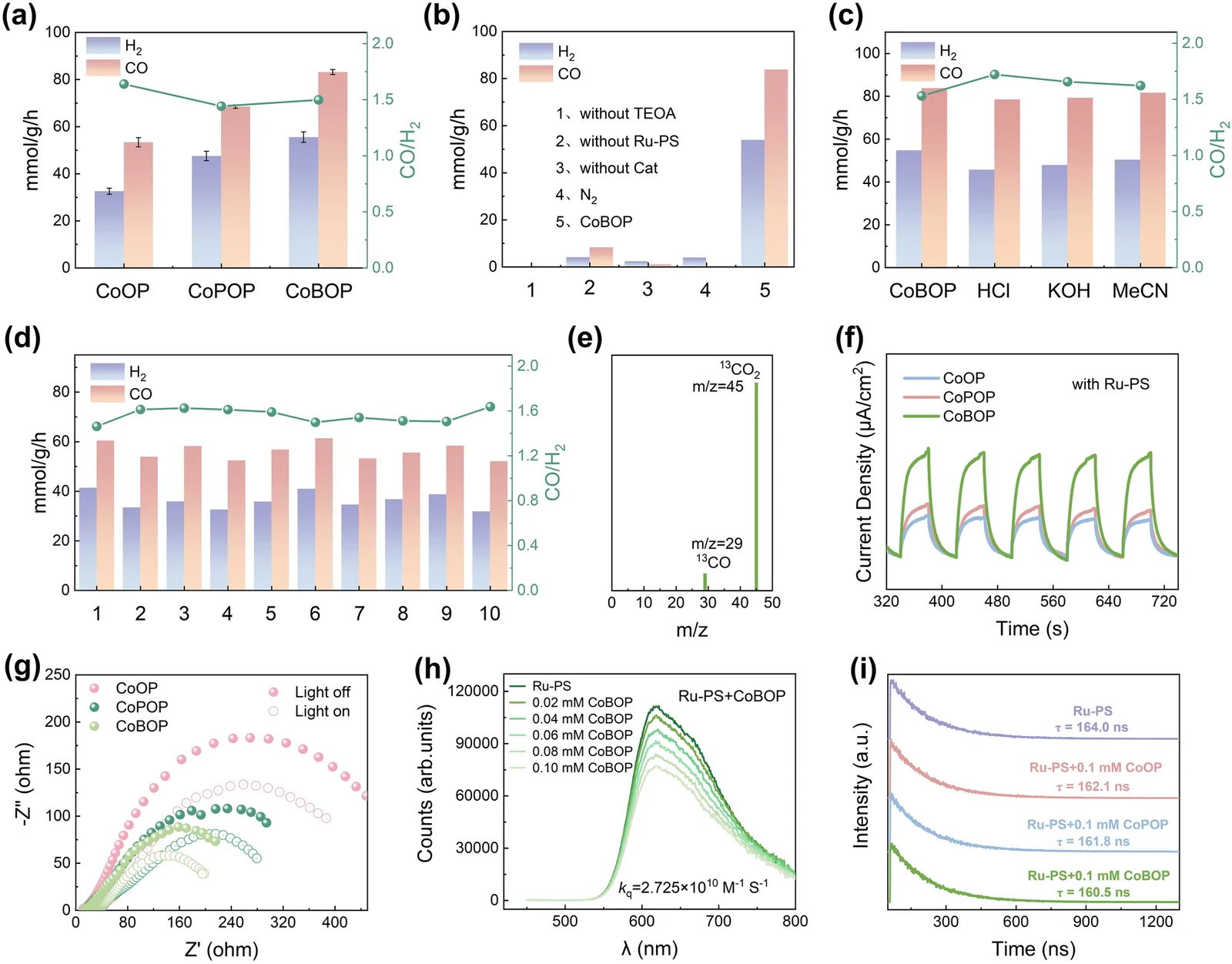
**a** Photocatalytic activity of CoOP, CoPOP, and CoBOP. **b** Photocatalytic activity of CoBOP under various reaction conditions (Ru-PS: [Ru(bpy)_3_]Cl_2_; Cat: catalysts; without Ru-PS (10 mg Cat)). **c** Photocatalytic activity of CoBOP after immersion in different solutions. **d** Durability measurements of CoBOP (2 mg). **e** ^13^C isotope tests under a ^13^CO_2_ atmosphere. **f** Transient photocurrent response of CoOP, CoPOP, and CoBOP at 0.2 M Na_2_SO_4_ electrolyte with [Ru(bpy)_3_]Cl_2_. **g** EIS plots of the CoOP, CoPOP, and CoBOP at 0.2 M Na_2_SO_4_ electrolyte with [Ru(bpy)_3_]Cl_2_. **h** Steady-state PL spectra of a CH_3_CN solution containing 0.05 mM Ru-PS in the presence of 0 ~ 0.10 mM CoBOP, respectively. **i** Time-resolved PL decay spectra of a CH_3_CN solution containing 0.05 mM Ru-PS in the presence of 0.10 mM CoOP, CoPOP, and CoBOP, respectively

To comprehensively assess the photocatalytic performance of CoOP, CoPOP, and CoBOP, pCO_2_RR experiments were carried out in acetonitrile (MeCN)/water mixed solution, while [Ru(bpy)_3_]Cl_2_ (Ru-PS) and triethanolamine (TEOA) were used as a photosensitizer and sacrificial agent, respectively. Encouragingly, as shown in Fig. 3a, CoOP, CoPOP, and CoBOP all show excellent performance of pCO_2_RR to syngas, and no other products were detected (Fig. S38). Compared to CoOP (CO 53.6 mmol g^−1^ h^−1^, H_2_ 32.6 mmol g^−1^ h^−1^, CO/H_2_ = 1.64) and CoPOP (CO 68.8 mmol g^−1^ h^−1^, H_2_ 46.2 mmol g^−1^ h^−1^, CO/H_2_ = 1.49), CoBOP exhibits a record production yield for CO of 83.7 mmol g^−1^ h^−1^ and H_2_ of 54.7 mmol g^−1^ h^−1^ (CO/H_2_ = 1.53), representing the best performance in pCO_2_RR systems with photosensitizers and sacrificial agents on the COF photocatalysts reported to date (Table S1). Furthermore, time-dependent photocatalytic CO_2_ reduction tests were carried out for CoBOP (Fig. S39). The results show that both CO and H_2_ yields increase progressively within 3 h, while the CO/H_2_ ratio remains essentially stable (1.62–1.67). In addition, the apparent quantum efficiency (AQE) values of CoBOP at different wavelengths are shown in Fig. S40 [54]. The wavelength-dependent trend is consistent with the absorption profile of the photosensitizer [Ru(bpy)_3_]Cl_2_, and the maximum AQE is observed to be 0.065% at 420 nm.

Further explorations reveal that almost no CO can be detected in the absence of TEOA, photocatalyst, or CO_2_ (Fig. 3b), implying the essentiality of these components for the pCO_2_RR. CoBOP also demonstrates exceptional photocatalytic efficiency in reducing CO_2_ to syngas within a system that does not require a photosensitizer (CO 8.2 mmol g^−1^ h^−1^, H_2_ 4.0 mmol g^−1^ h^−1^). All of the above observations confirm the outstanding stability of the obtained Pc-based COFs. As depicted in Fig. 3c, CoBOP after being treated with different solutions still displays remarkable photocatalytic performance similar to the pristine CoBOP, with CO/H_2_ ratios maintained within 1.53–1.72, confirming the impressive stability of CoBOP endowed by the irreversible bond covalent linking. In addition, CoBOP can be recycled at least 10 times with only a slight decrease in photocatalytic performance, while maintaining CO/H_2_ ratios in the range of 1.46–1.64 (Fig. 3d), highlighting the robustness of the syngas composition under prolonged operation. As shown in the XRD and FTIR spectra (Fig. S41), the structural integrity of the catalyst remains well preserved after the photocatalytic reaction. The diffraction patterns exhibit no significant shifts or loss of crystallinity, and the FT-IR spectra show consistent vibrational features before and after catalysis. These results collectively indicate that the framework structure of the COF catalyst retains its stability under the reaction conditions. As illustrated in Fig. S42, no linear correlation is observed between the catalyst loading and the pCO_2_RR performance. Intriguingly, a smaller amount of catalyst often results in enhanced activity. Moreover, the result of the isotope experiment using ^13^CO_2_ as a reactant further verifies that CO is derived from the CO_2_ species rather than other carbon-containing compounds (Fig. 3e).

### Exploration of Structure–property Correlation

To gain insight into the underlying reasons for the remarkable pCO_2_RR performance of the proposed COFs and the difference in photocatalytic performance among CoOP, CoPOP, and CoBOP, the photoelectrochemical features of the three catalysts were probed. Firstly, the transient photocurrent response was carried out. The photocurrent intensities of the three catalysts are ordered as CoOP > CoPOP > CoBOP with pure 0.2 M Na_2_SO_4_ as electrolyte (Fig. S43). This order is opposite to their pCO_2_RR performance. Considering the involvement of the photosensitizer in the actual photocatalytic reaction system, the transient photocurrent response was further investigated in an electrolyte containing the Ru-PS. Interestingly, the order of the photocurrent intensities of the three photocatalysts turns reversed, CoBOP > CoPOP > CoOP (Fig. 3f), matching well with their photocatalytic performances. Moreover, the electrochemical impedance spectroscopy (EIS) results exhibit an order reversal in the presence and absence of the photosensitizer (CoOP < CoPOP < CoBOP without Ru-PS, CoBOP < CoPOP < CoOP with Ru-PS) (Fig. S44), indicating CoBOP possesses the lowest interfacial charge transport resistance when the photosensitizer is present. Furthermore, to better investigate the influence of sunlight irradiation on the charge transfer process, the EIS spectra under light-on and light-off conditions were compared, as shown in Fig. 3g. The results reveal that under light-on conditions, the arc radius of the catalysts is reduced compared to those of in the dark state, suggesting that sunlight excitation promotes charge separation and facilitates interfacial charge transfer. In addition, steady-state and time-resolved PL measurements without Ru-PS (Fig. S45) show that CoBOP exhibits the strongest emission and shortest carrier lifetime, indicating a higher tendency for electron–hole recombination. Nevertheless, CoBOP achieves the highest photocatalytic activity, demonstrating that intrinsic recombination does not govern the pCO_2_RR performance. Commonly, the excited photosensitizer is regarded as a paramount photogenerated electron donor when it exists in the photocatalytic system [55]. Under these circumstances, the photocurrent intensity depends more on the rate of electron transfer from the photosensitizer to the catalyst. Therefore, the higher photocurrent intensity and superior photocatalytic performance of CoBOP compared to the other two catalysts should be attributed to its better photoelectron transfer capacity from the excited Ru-PS to the active site.

1 $$\frac{{I}_{0}}{I}=1+K\left[Q\right]=1+{k}_{q}{\tau }_{0}[Q]$$

The steady-state photoluminescence (PL) and time-resolved PL decay spectra further verify this inference. The Steady-state PL spectra of a CH_3_CN solution with 0.05 mM Ru-PS in the presence of 0 ~ 0.10 mM CoBOP were measured, respectively (Fig. 3h). The apparent quenching constant (k_q_) for CoBOP obtained by the Stern–Volmer (S-V) plots (Eq. 1 [56]) between Ru-PS and CoBOP is 2.725 × 10^10^ M^−1^ S^−1^ (Fig. S46), obviously higher than that of CoPOP (1.580 × 10^10^ M^−1^ S^−1^) and CoOP (9.834 × 10^9^ M^−1^ S^−1^) (Figs. S47 and S48). Moreover, time-resolved transient spectroscopy studies display the shortest lifetime of 160.5 ns for Ru-PS + CoBOP compared with 161.8, 162.1, and 164.0 ns for the Ru-PS + CoPOP, Ru-PS + CoOP, and original Ru-PS, respectively (Fig. 3i). Both of them indicate that CoBOP has the highest photoelectron transfer efficiency from the photosensitizer compared to the CoPOP and CoOP, which agrees well with the results of transient photocurrent response and EIS [55].

**Fig. 4:**
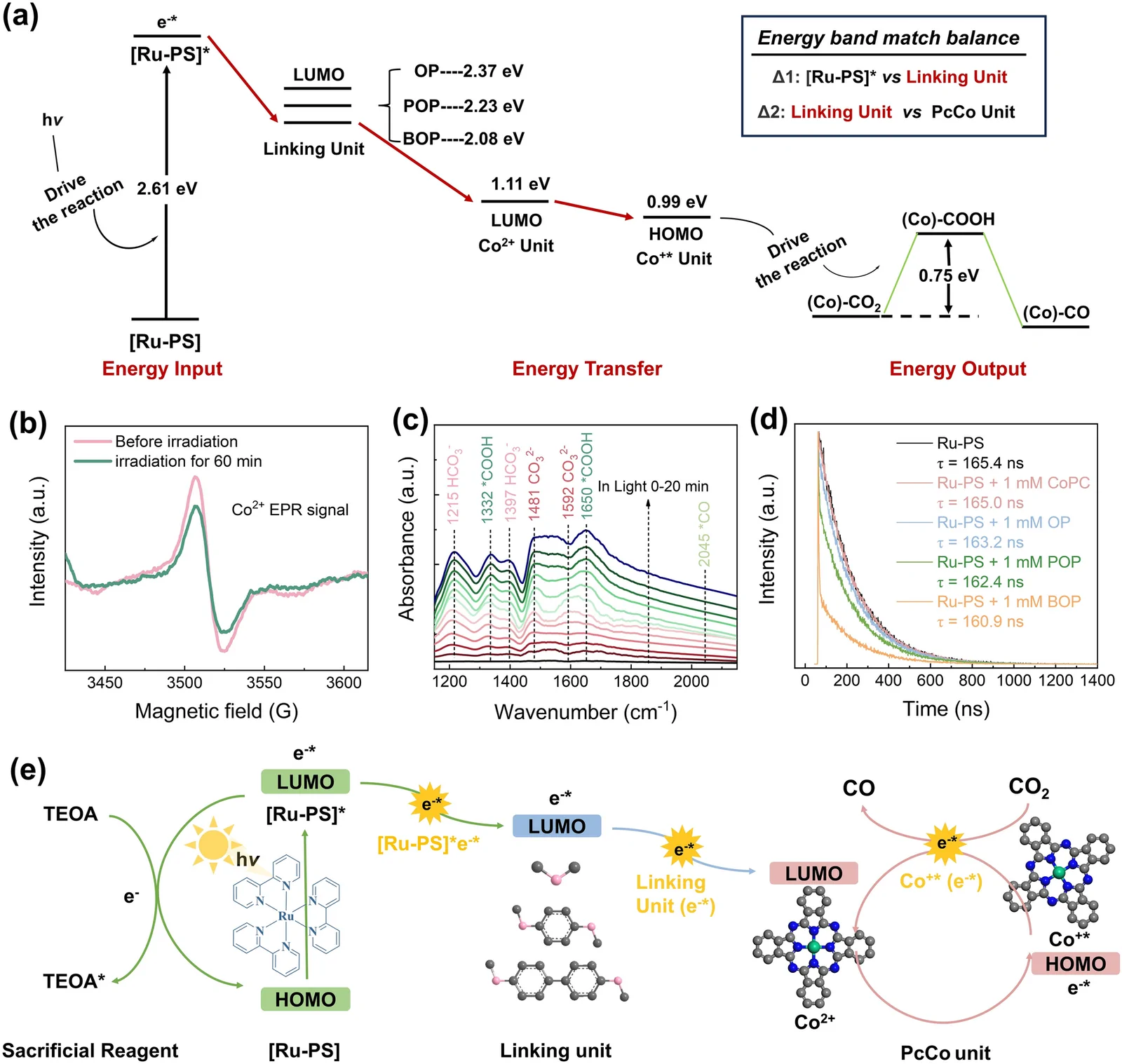
**a** DFT calculations of the excited electron (e^−^*) transfer mechanism. **b** Electron paramagnetic resonance spectra of CoBOP with various states. **c** In situ FT-IR spectroscopy during photocatalytic CO_2_RR over CoBOP. **d** Time-resolved PL decay spectra of a CH_3_CN solution containing 0.05 mM Ru-PS in the presence of 1.0 mM OP, POP, BOP, and CoPC, respectively. **e** The excited electron (e^−^*) transfer mechanism during the pCO_2_RR process for catalysts

For further understanding of the electron transfer process in theory, especially the underlying reasons for the outstanding pCO_2_RR performance of CoBOP, DFT calculations were performed (Fig. 4a) [57]. Firstly, under sunlight irradiation, the photosensitizer [Ru(bpy)_3_]Cl_2_ captures photons with a wavelength of 474 nm, generating electron–hole pairs via excitation. This raises [Ru(bpy)_3_]Cl_2_ from its ground state Ru-PS to the excited state Ru-PS*, where the excited electron (e^−^*) resides in the LUMO of Ru-PS with an additional energy of 2.61 eV. The DFT calculations show the LUMO of OP, POP, and BOP linking units are 2.37, 2.23, and 2.08 eV, respectively, which all can be smoothly driven by the E(e^−^*) of 2.61 eV with energy declines (Δ1) of 0.24, 0.38, and 0.53 eV for OP, POP, and BOP linking units, respectively. Once the charge-separated state {Ru-PS(hole)}-{linking units(e^−^*)} is formed, the e^−^* journey would further fill into the LUMO energy of Co^2+^ catalytic active site from linking units, forming Co⁺, with energy drop gaps (Δ2) of 1.26 eV for OP, 1.12 eV for POP, and 0.97 eV for BOP. This makes the backtrace of e^−^* from Co^+^* to [Ru-PS](hole) impossible due to the high energy requirement; and therefore, the TEOA becomes the electron candidate to reduce [Ru-PS](hole) to [Ru-PS]. Quasi-in situ EPR analysis of CoBOP (Fig. 4b) reveals a distinct Co^2+^ signal before light irradiation. After 60 min of irradiation, the signal intensity decreases to some extent, indicating the conversion of Co^2+^ into EPR-silent Co^+^ species by accepting photogenerated electrons [58, 59]. The e^−^* in Co^+^* possesses an energy of 0.99 eV, aligning well with the energy barrier for CO_2_ reduction to CO (0.75 eV), after the formation of CO, the Co^+^* turns back to Co^2+^.

In situ FT-IR spectroscopy monitored possible intermediates formed during the pCO_2_RR over CoBOP (Fig. 4c). Distinct absorption bands corresponding to bicarbonate (HCO_3_^−^, 1215 and 1397 cm^−1^) and carbonate (CO_3_^2−^, 1481 and 1592 cm^−1^) are detected, which can be attributed to the dissolution of CO_2_ [60, 61]. In addition, characteristic peaks at 1332 and 1650 cm^−1^ are assigned to the key intermediate *COOH, whereas the subtle peak at 2045 cm^−1^, attributed to the intermediate *CO, is also observed. Notably, the intensities of these intermediate peaks increase progressively with prolonged light irradiation, indicating the stepwise formation and transformation of intermediates during the reaction. These results confirm that the photocatalytic CO_2_ reduction to CO over CoBOP follows the reaction pathway: CO_2_(g) → *COOH → *CO → CO(g).

According to the orbital theory, the large energy gap is inconvenient to the electron transfer, although it is a process of declining energy [62, 63]. Therefore, the direct electron transfer from the LUMO of Ru-PS* to the LUMO of Co^2+^ is difficult due to the high energy gap of 1.50 eV. Luckily, the linking unit acts as a ‘ladder’ between Ru-PS* and the LUMO of Co^2+^, allowing the electrons to cascade down and facilitate rapid transfer. The analyses of time-resolved transient spectroscopy for Ru-PS, Ru-PS with OP, Ru-PS with POP, Ru-PS with BOP, and Ru-PS with PcCo showcase that the photoelectron extraction efficiency of all the linking units (OP 163.2 ns, POP 162.4 ns, BOP 160.9 ns) are higher than Co^2+^ (PcCo 165.0 ns) (Fig. 4d), further verifying this inference. This should be responsible for the excellent pCO_2_RR performance of the as-prepared Pc-based COFs. Furthermore, among the three OP, POP, and BOP linking units, the LUMO of BOP (2.08 eV) sits relatively middle of Ru-PS* (2.61 eV) and the LUMO of Co^2+^ (1.11 eV) compared to that of OP (2.37 eV) and POP (2.23 eV), making closer Δ1 (0.53 eV) and Δ2 (0.97 eV). Which should promote the electron transfer between Ru-PS* and the LUMO of Co^2+^, and then enhance the pCO_2_RR performance of CoBOP. Thus, modifying the linking units within Pc-based COFs using irreversible covalent bonds effectively optimizes their electronic properties, significantly promoting their pCO_2_RR performance. The whole potential photocatalytic processes are shown in Fig. 4e.

## Conclusions

In summary, one type of Pc-based COF photocatalysts with tunable conjugation length linking unit and irreversible covalent bonds was designed and successfully synthesized. Benefiting from the ether connection, all CoOP, CoPOP, and CoBOP exhibited superior thermal and chemical structural stabilities. CoBOP, with a long-conjugated linking unit, demonstrated an outstanding pCO_2_RR to syngas performance with CO productivity of 83.7 mmol g^−1^ h^−1^ and H_2_ productivity of 54.7 mmol g^−1^ h^−1^. Experimental analysis and theoretical calculations highlighted that the tunable linking unit endowed the COF catalysts with adjustable photoelectronic identities, which could effectively promote the electron injection from the excited Ru-PS sensitizer to the catalytically active sites of the photocatalyst, thereby enhancing the pCO_2_RR performance. The optimally matched LUMO position of BOP linking unit between the excited photosensitizer and active Co^2+^ unit resulted in easier electron transfer, leading to its superior photocatalytic performance. This work provides innovative insights into the design and development of highly stable, superior photocatalytic performance, and chemical structure tunable photocatalysts.

## Supplementary Information

Below is the link to the electronic supplementary material.

Supplementary file1 (DOCX 26577 KB)
