# Supplementary material for: Construction of Modifiable Phthalocyanine-Based Covalent Organic Frameworks with Irreversible Linking for Efficient Photocatalytic CO2 Reduction
Source: Nanomicro Lett. 2026 Jan 15;18:119. doi: 10.1007/s40820-025-01967-y (PMC12804500; doi:10.1007/s40820-025-01967-y)
Supplement: Supplementary file 1 — Supplementary file1 (DOCX 26577 KB) [file 40820_2025_1967_MOESM1_ESM.docx]

Supporting Information for

**Construction of Modifiable Phthalocyanine-Based Covalent Organic Frameworks with Irreversible Linking for Efficient Photocatalytic CO_2_ Reduction**

Xuefei Zhou^1#^, Shaowei Yang^1#^, Zhengyang Hu^1^, Zhanwei Chen^1^, Ying Guo^1^, Tianshuai Wang^1^*, Qiuyu Zhang^1^*, Hepeng Zhang^1^*

^1^ Xi’an Key Laboratory of Functional Organic Porous Materials, School of Chemistry and Chemical Engineering, Northwestern Polytechnical University, 710129 Xi’an, P. R. China

^#^Xuefei Zhou and Shaowei Yang contributed equally to this work.

*Corresponding authors. E-mail: [tianshuai@nwpu.edu.cn](mailto:tianshuai@nwpu.edu.cn) (Tianshuai Wang); [qyzhang@nwpu.edu.cn](mailto:qyzhang@nwpu.edu.cn) (Qiuyu Zhang); [zhanghepeng@nwpu.edu.cn](mailto:zhanghepeng@nwpu.edu.cn) (Hepeng Zhang)

**S1 Experimental Section**

**S1.1 Chemicals**

4-nitrophthalonitrile (98%) was purchased from Shanghai Maclin Biochemical Technology Co., Ltd. 4-hydroxyphthalonitrile (≥97%), hydroquinone (AR) and 4,4-dihydroxydiphenyl (99%), Cobalt(II) acetate tetrahydrate (≥99.9%) and tris(2,2′-bipyridyl)dichlororuthenium(II) (98%) were purchased from Shanghai Aladdin Biochemical Technology Co., Ltd. 1,8-diazabicyclo[5.4.0]undec-7-ene (DBU) (99%) and n-pentanol (GC, >99%) were purchased from Shanghai Yien Chemical Technology Co., Ltd. N, N-Dimethylformamide (DMF) (AR), Acetonitrile (MeCN) (AR), Triethanolamine (TEOA) (AR), ethanol (AR) and Sodium sulfate anhydrous (AR) were purchased from Guangdong Guanghua Sci-Tech Co., Ltd. Potassium carbonate anhydrous (AR) was purchased from [Tianjin Kemiou Chemical Reagent Co.,](http://www.tjkermel.com/) Ltd. The above reagents were of analytical grade and used directly without any further purification before the experiment. Nafion solution (5 wt%) was purchased from Sigma-Aldrich LLC.

**S1.2 Characterization**

The ^1^H NMR measurement was performed using Bruker Advance 500MHz Bruke. IR spectra were confirmed through Nicolet™ iS50 FTIR Spectrometer. Powder X-ray diffraction (PXRD) patterns of catalysts were recorded on a Brooke GmbH X-ray diffractometer (Germany). The 2θ angle ranged from 1° to 50° with a scan step of 5°/min. Field emission scanning electron microscopy (FE-SEM, FEI Verious G4) and transmission electron microscopy (TEM, FEI Talos F200X TEM, operating voltage 200 kV) were used to study the morphology and structure of the samples. N_2_ and CO_2_ adsorption-desorption was measured with the 3H-2000PS2 type PS2-0790 Surface Area Porosity Analyzer. The element composition of the samples was analyzed by X-ray photoelectron spectroscopy (XPS, Axis Ultra DLD by Kratos Company in the United Kingdom.

**S1.3 Photocurrents and Mott-Schottky (MS) curve measurements**

5 mg of catalysts were meticulously distributed within a solution comprising Nafion (100 µL) and ethanol (900 µL), followed by ultrasonic treatment. Apply 200 µL of the solution dropwise on the surface of the FTO conductive glass and air dry. The FTO glass, coated with the catalyst, acted as a photoelectrode. A conventional system of a three-electrode cell in a solution of pH = 7 was used.

**S1.4 Photocatalytic CO_2_RR Cyclic experiments**

Following 1 hour of illumination, the catalyst is isolated from the reaction solution via filtration. Wash with ethanol and deionized water several times during the filtration process. Subsequently, vacuum drying at 60 °C yields the regenerated solid sample. Upon adding equivalent volumes of fresh acetonitrile, H_2_O, and [Ru(bpy)_3_]Cl_2_·6H_2_O, CO_2_ is reintroduced into the photocatalytic system for the next photocatalytic cycle.

**S1.5 Determination of quenching rate (k_q_)**

A reported method [S1] was followed to calculate the kq in the steady-state measurements, where the fluorescence intensity of photo-excited Ru-PS in the presence of a quencher was measured and fitted to the Stern-Volmer equation.

$\frac{I_{O}}{I}=1+K\left[ Q \right]=1+k_{q}\tau_{0}[Q]$ (S1)

In this equation, *I*_0_ and *I* are the fluorescence intensity values in the absence and presence of the quencher, *K* is the Stern-Volmer constant for dynamic quenching, *k_q_* is the apparent rate of bimolecular quenching, *τ*_0_ is the lifetime of the excited state without quencher, and [Q] is the concentration of the quencher.

**S1.6 Apparent quantum efficiency**

The apparent quantum efficiency (AQE) measurements for CoBOP under monochromatic irradiation. Specifically, AQE was determined at 365, 420, 500, 600, 700, and 800 nm using the following equation:

$$AQE \left( \% \right)=\frac{{2\times n}_{co}{\times N}_{A}\times h\times c}{S\times P\times T\times\lambda}\times100\%$$

Where *n_co_* is the molar amount of CO, *N_A_* is Avogadro’s constant (6.022 × 10^23^ mol^-1^), *h* is Planck’s constant (6.63 × 10^-34^ m^2^ kg s^-1^), *c* is the speed of light (3 × 10^8^ m·s^-1^), *S* is the irradiation area (cm^2^), *P* is the irradiation intensity (W cm^-2^), *T* is the irradiation time (s), and *λ* is the wavelength of the incident light.

**S1.7 Computation methods**

All quantum chemical calculations were performed with the Gaussian 16 program using the level of B3LYP/6-311+G(d,p) [S2].

**S2 Supplementary Figures and Tables**

**
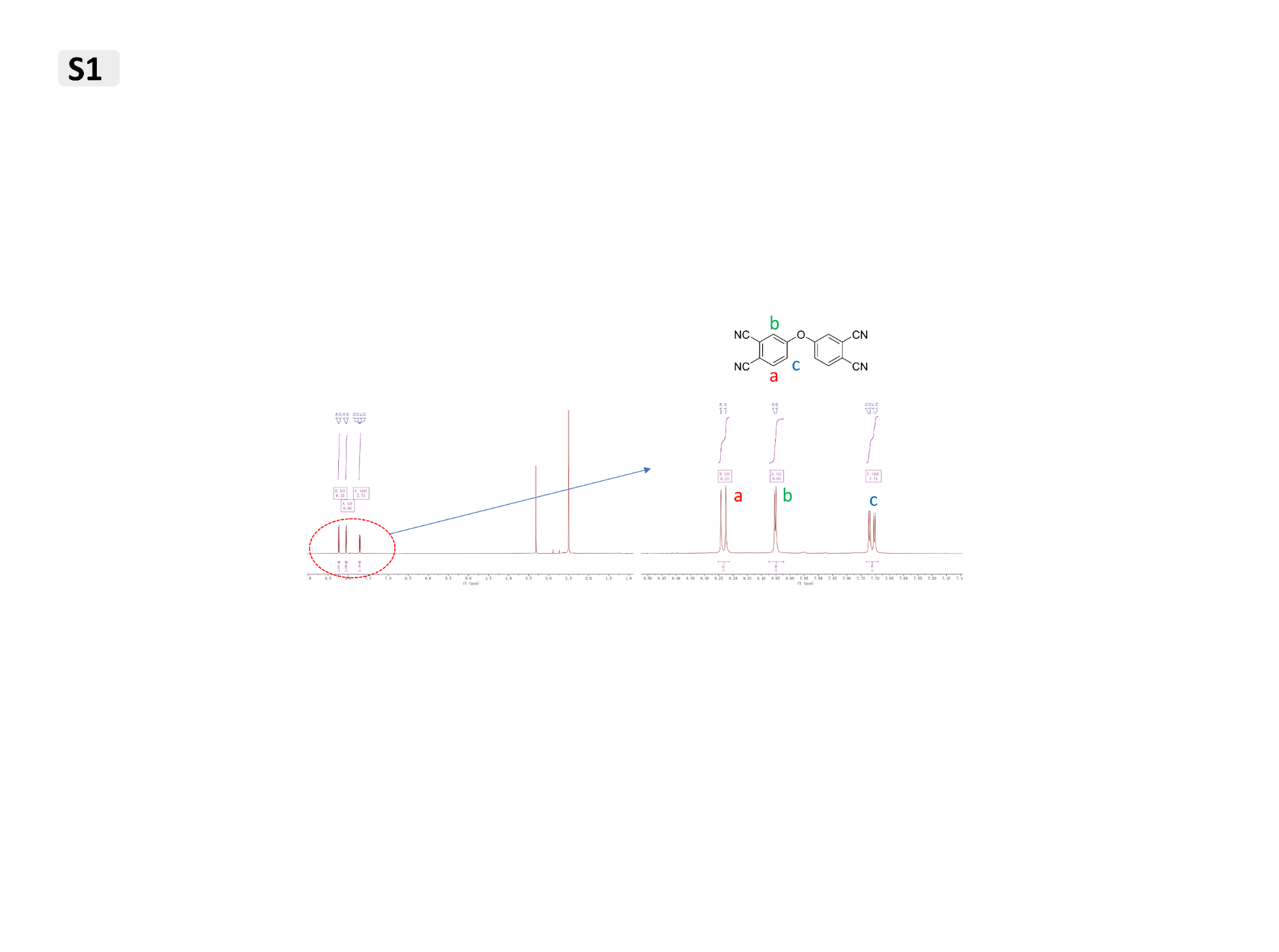
**

**Fig. S1** ^1^H NMR spectra of OP. (500 MHz, DMSO-d6) δ 8.23 (d, J = 8.7 Hz, 2H), 8.05 (d, J = 2.6 Hz, 2H), 7.71 (dd, J = 8.7, 2.5 Hz, 2H)


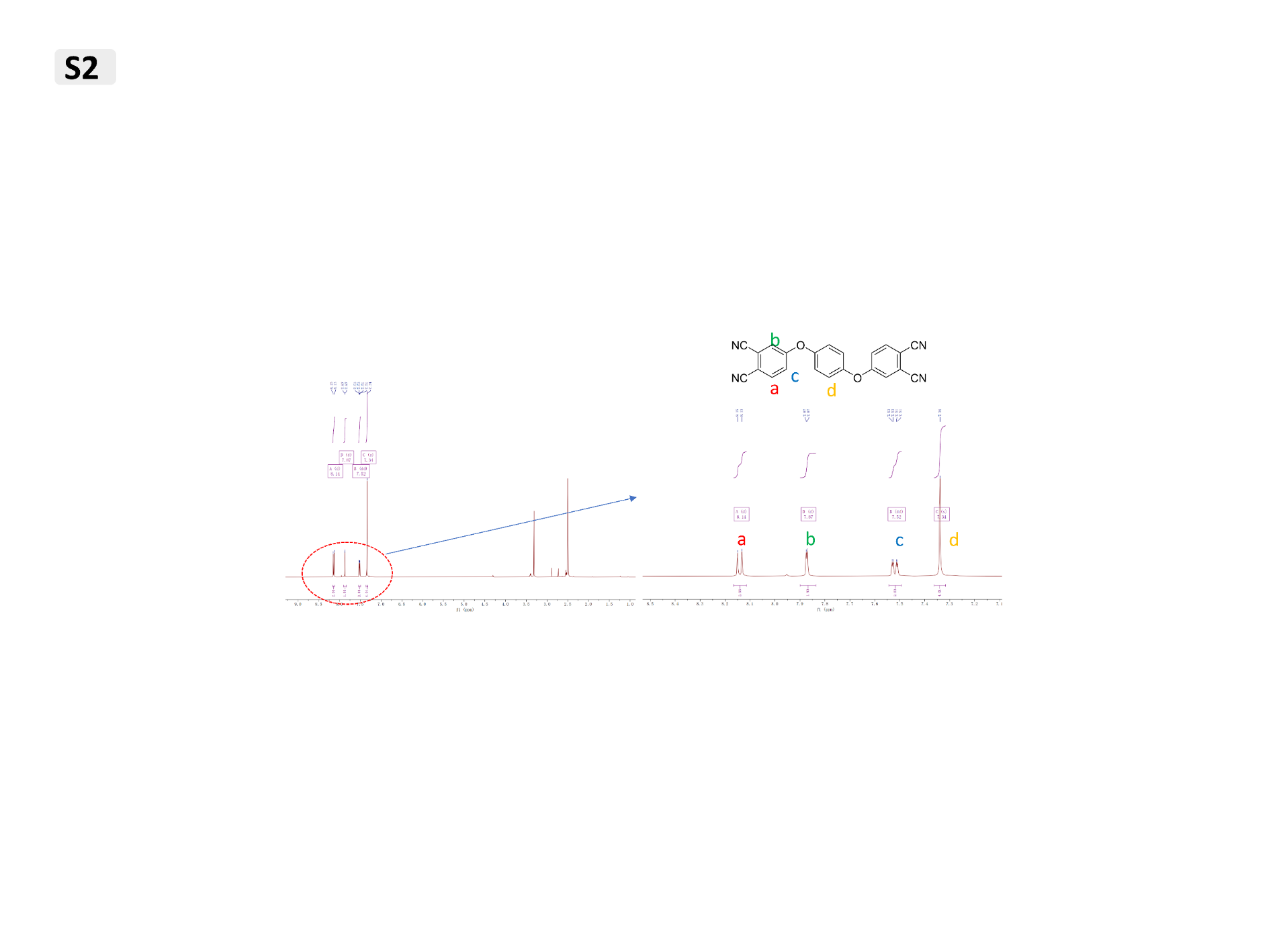


**Fig. S2** ^1^H NMR spectra of POP. (500 MHz, DMSO-d6) δ 8.14 (d, J = 8.8 Hz, 2H), 7.87 (d, J = 2.6 Hz, 2H), 7.52 (dd, J = 8.8, 2.7 Hz, 2H), 7.34 (s, 4H)


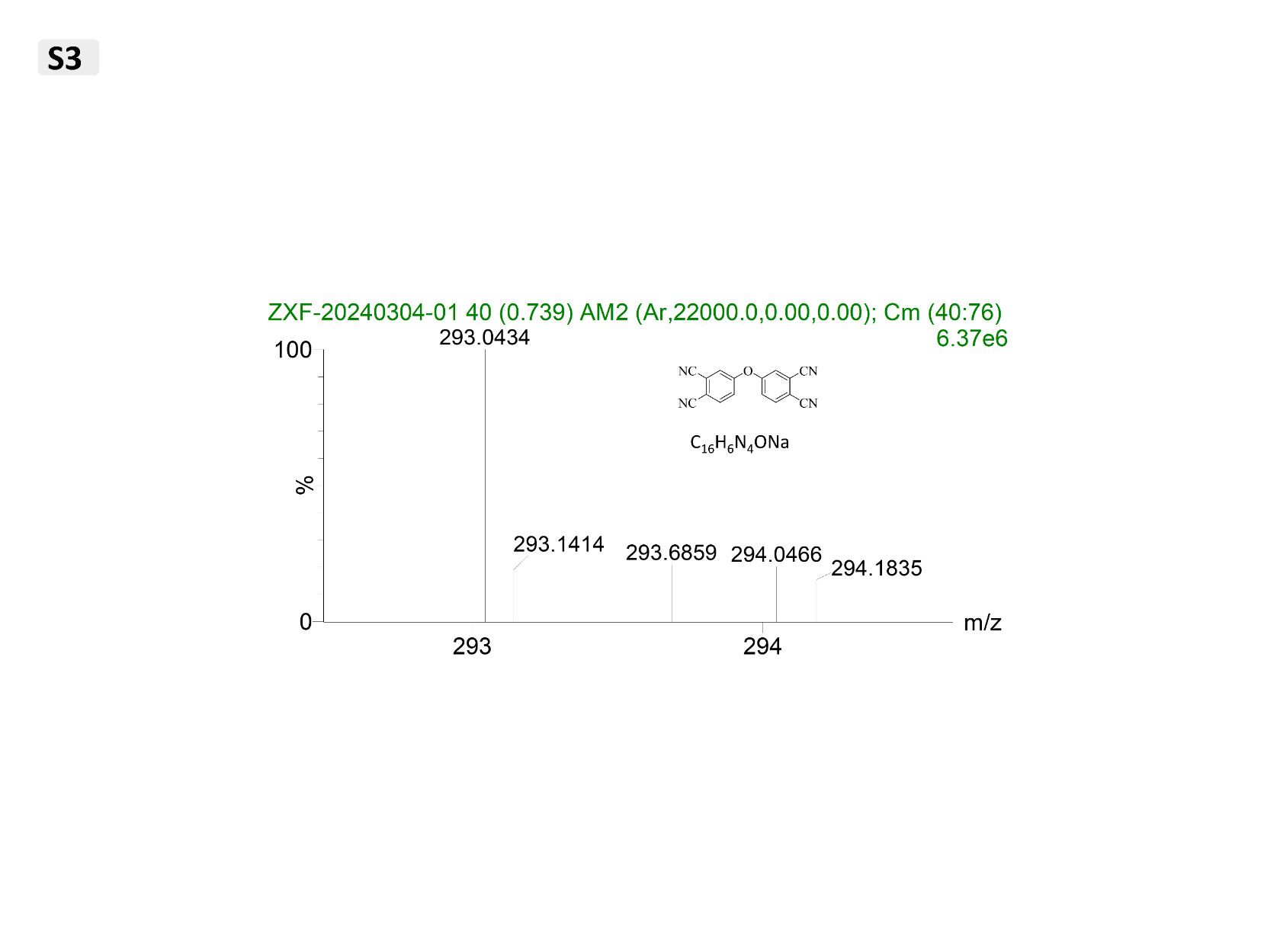


**Fig. S3** HR-MS spectrum of OP


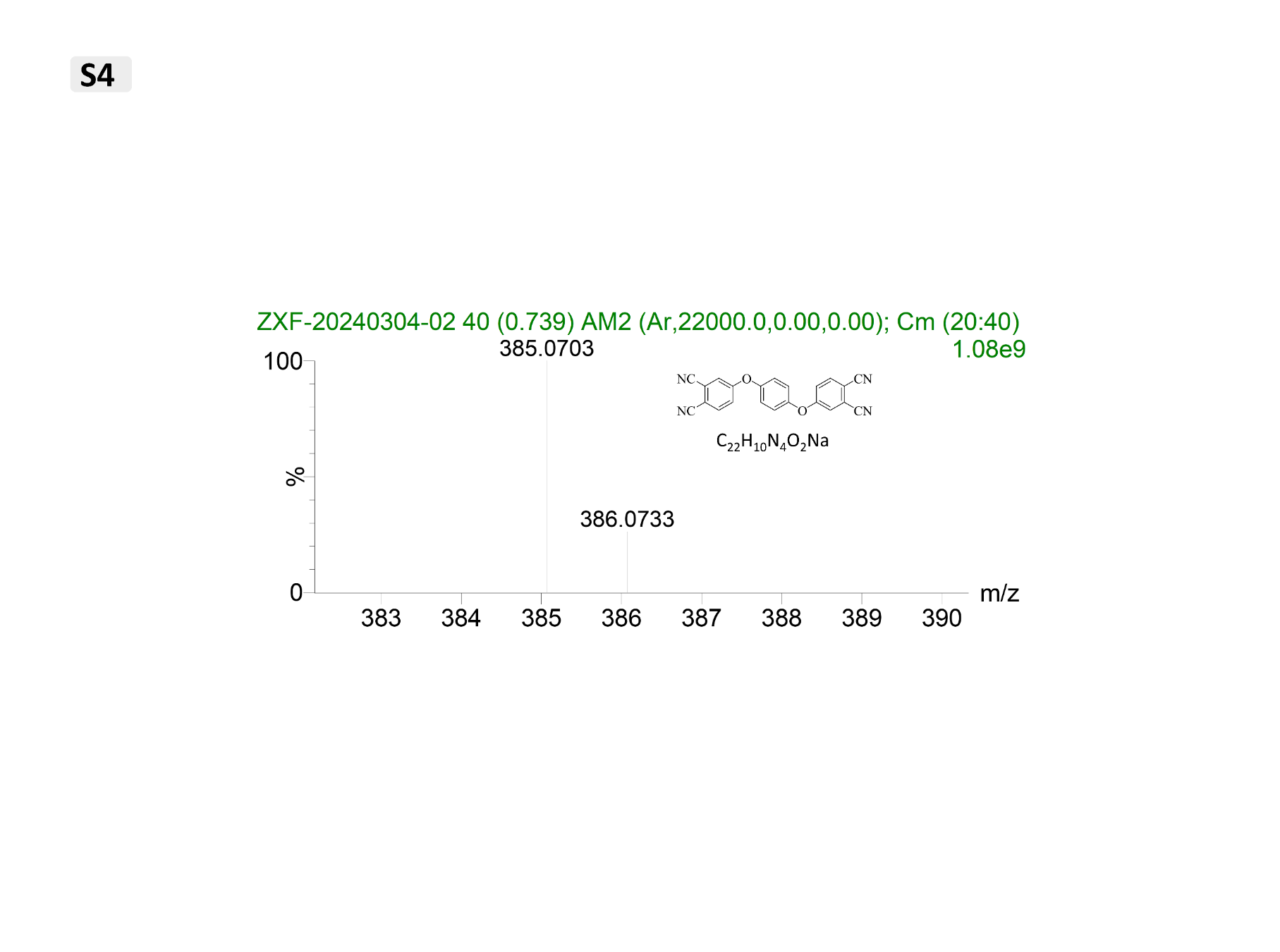


**Fig. S4** HR-MS spectrum of POP


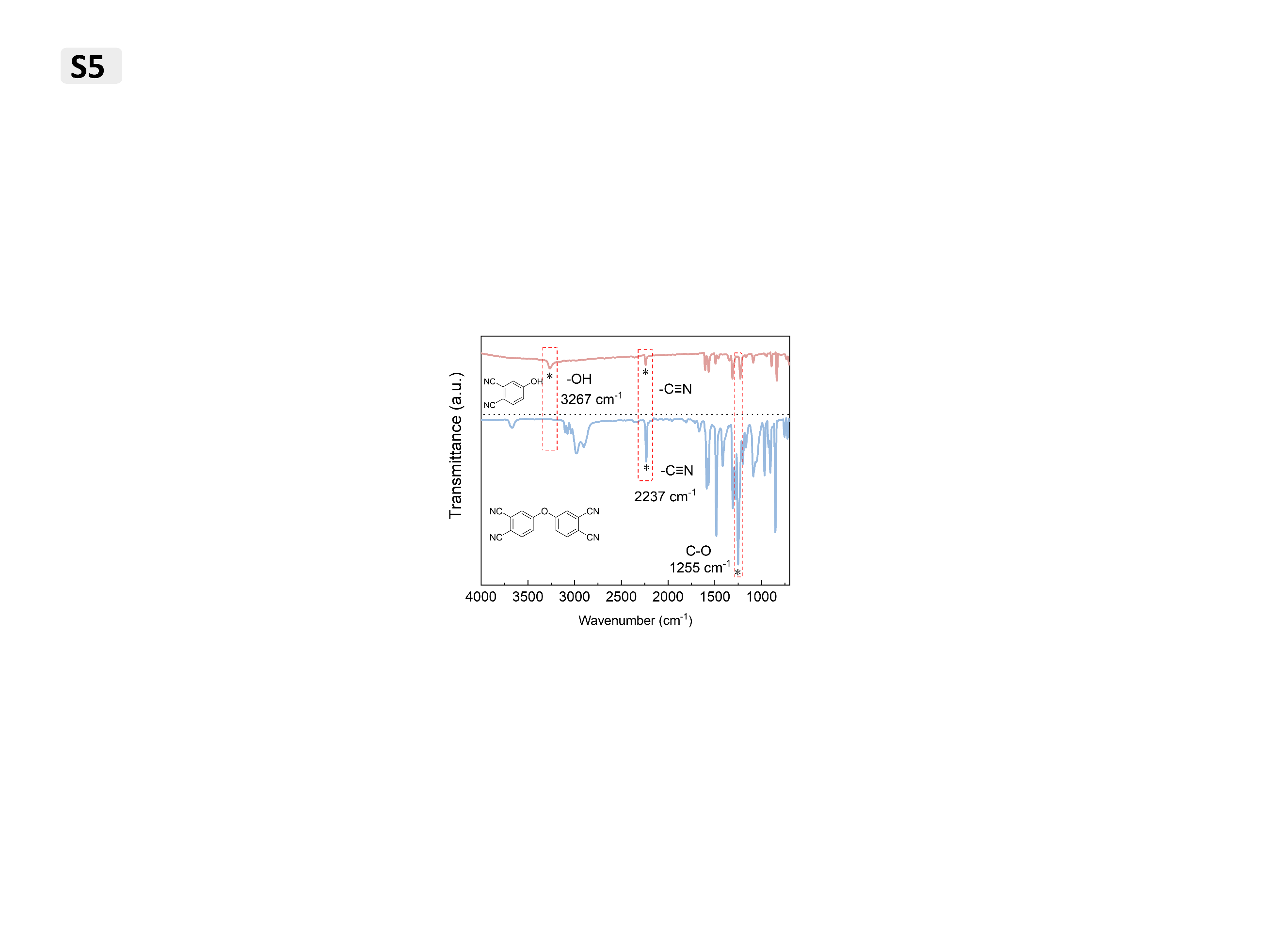


**Fig. S5** FT-IR spectra of 4-Nitrophthalonitrile and OP


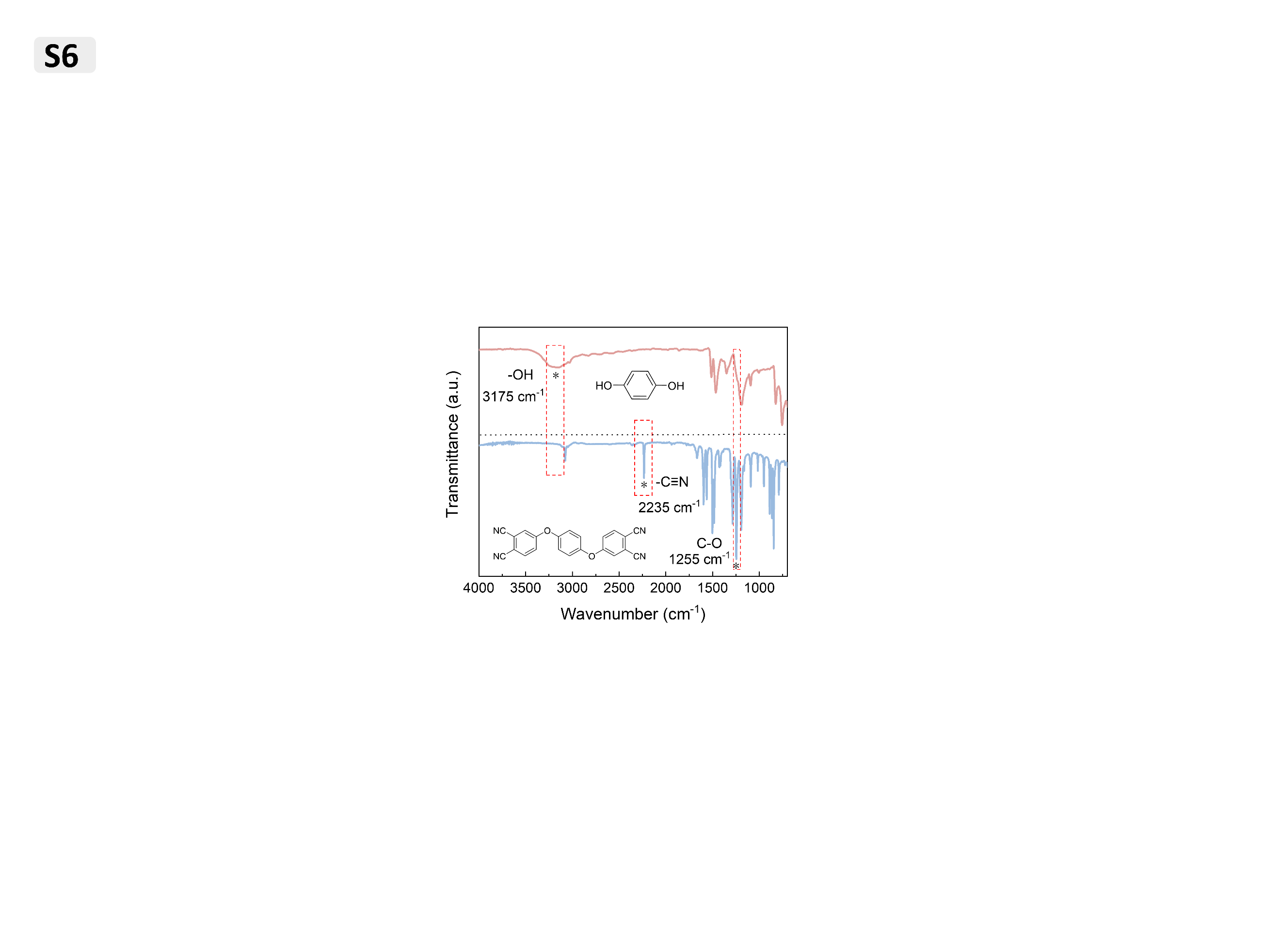


**Fig. S6** FT-IR spectra of hydroquinone and POP


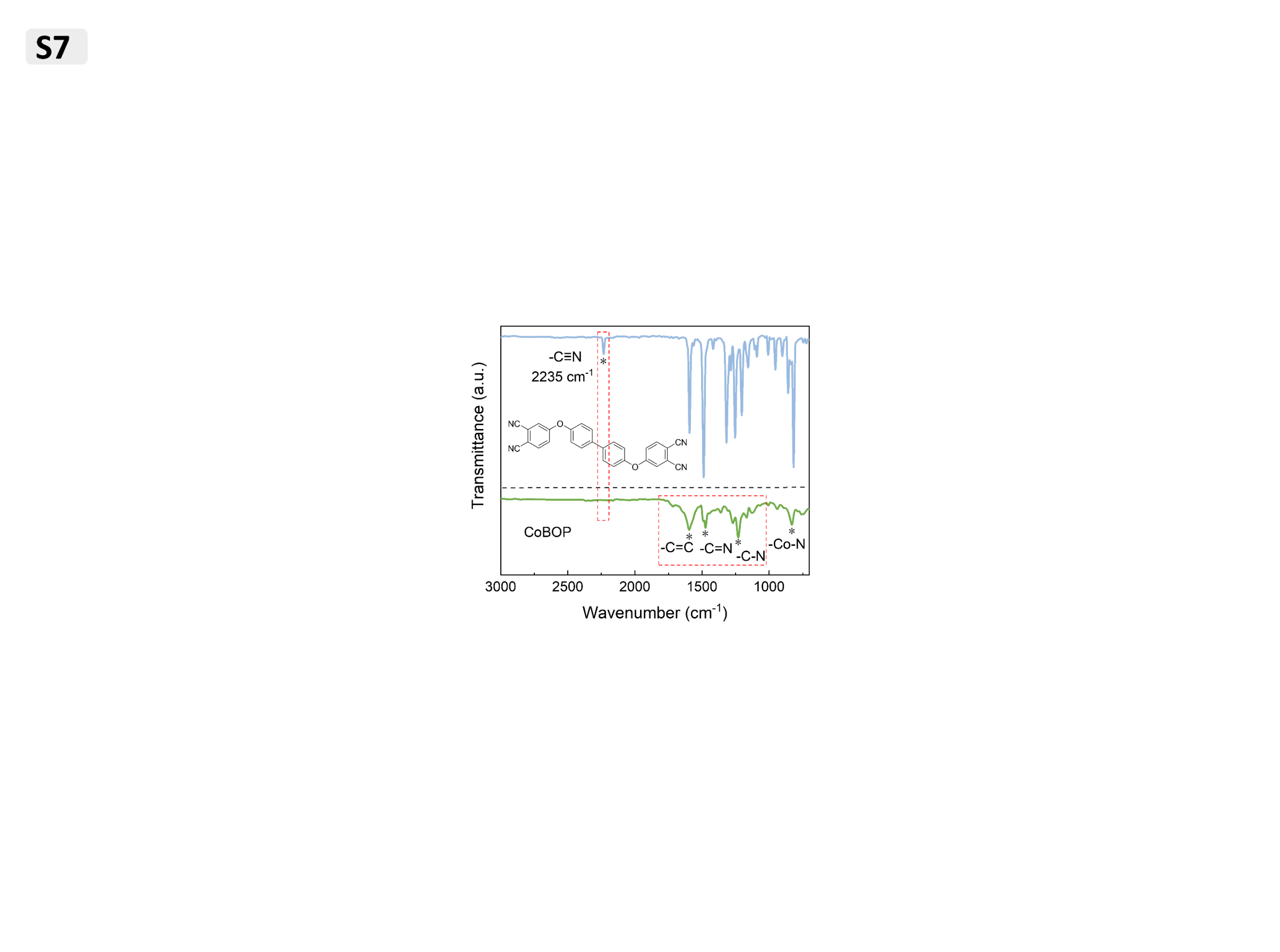


**Fig. S7** FT-IR spectra of BOP and CoBOP


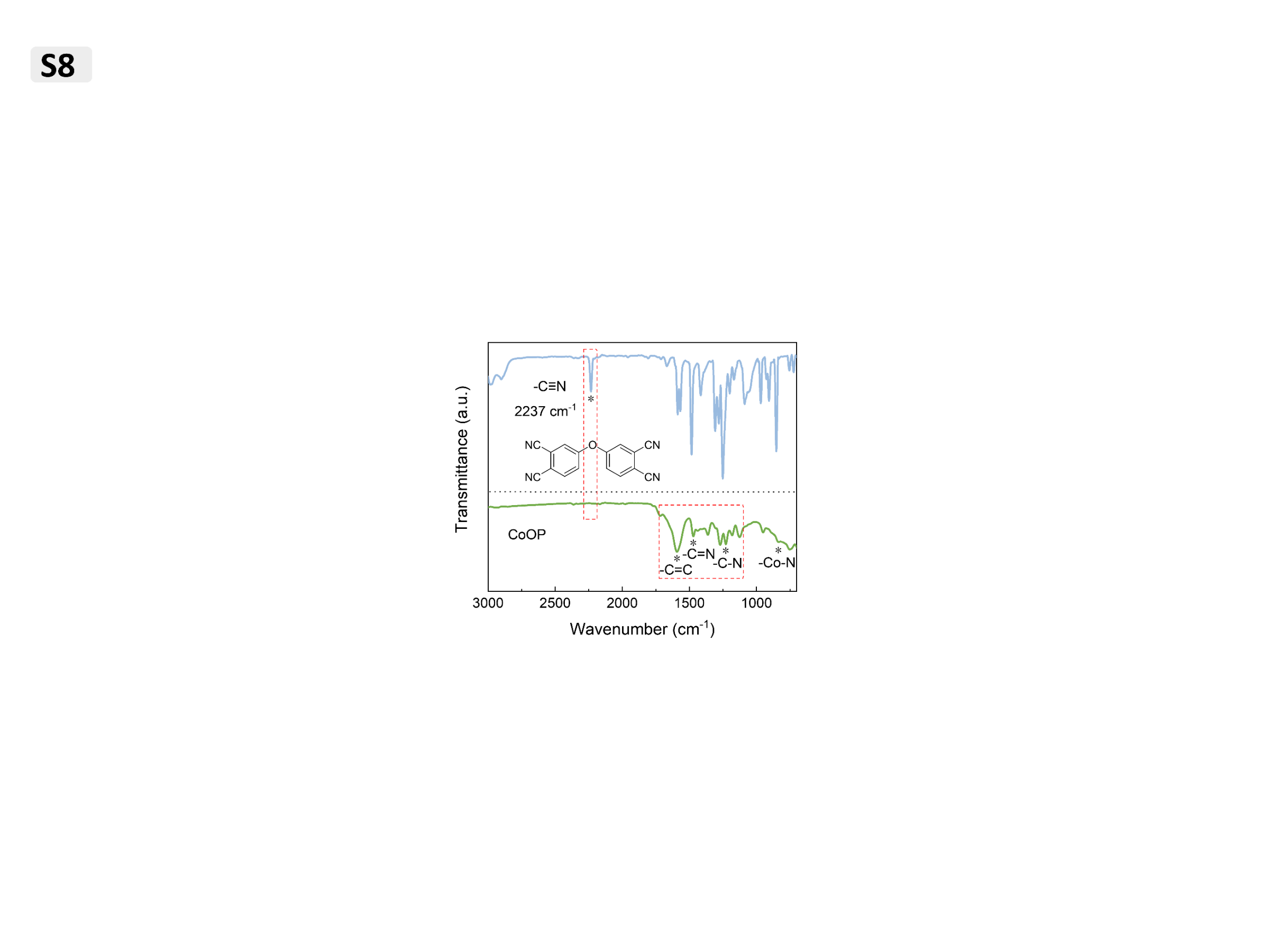


**Fig. S8** FT-IR spectra of OP and CoOP


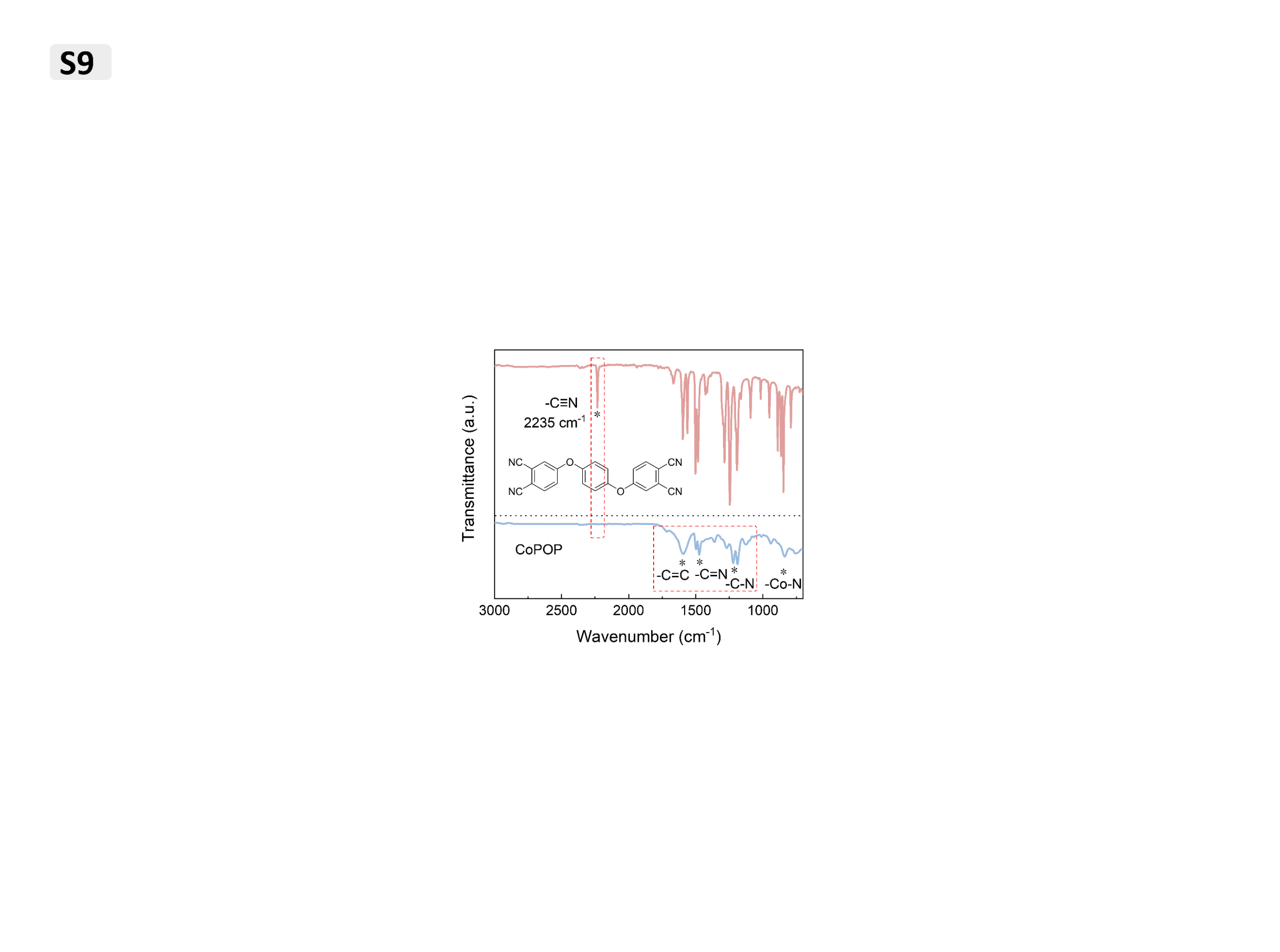


**Fig. S9** FT-IR spectra of POP and CoPOP


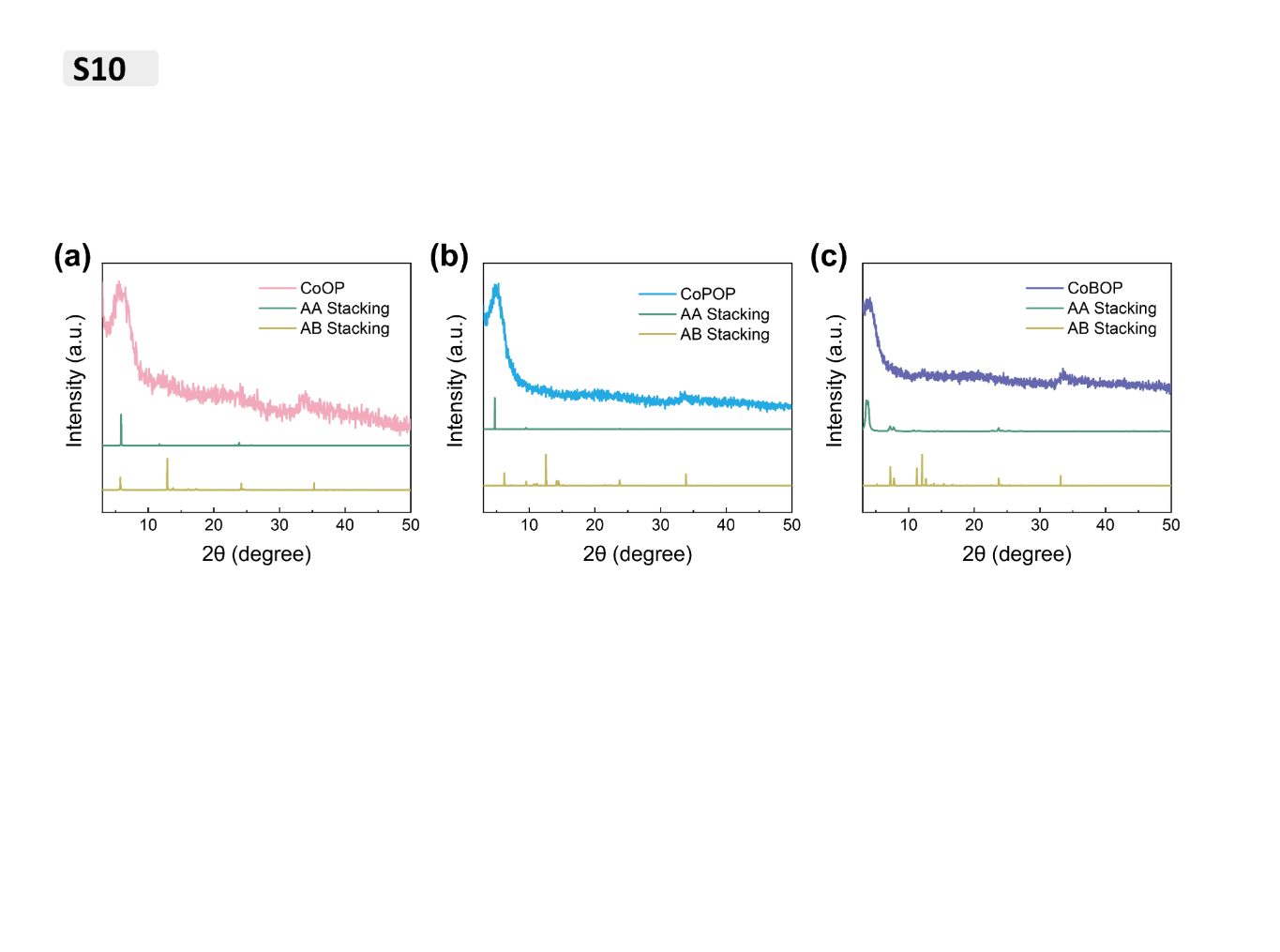


**Fig. S10** AB Stacking simulated PXRD crystal structure based on theoretical structural simulation using the Materials Studio package of CoOP **a**, CoPOP **b**, and CoBOP **c**


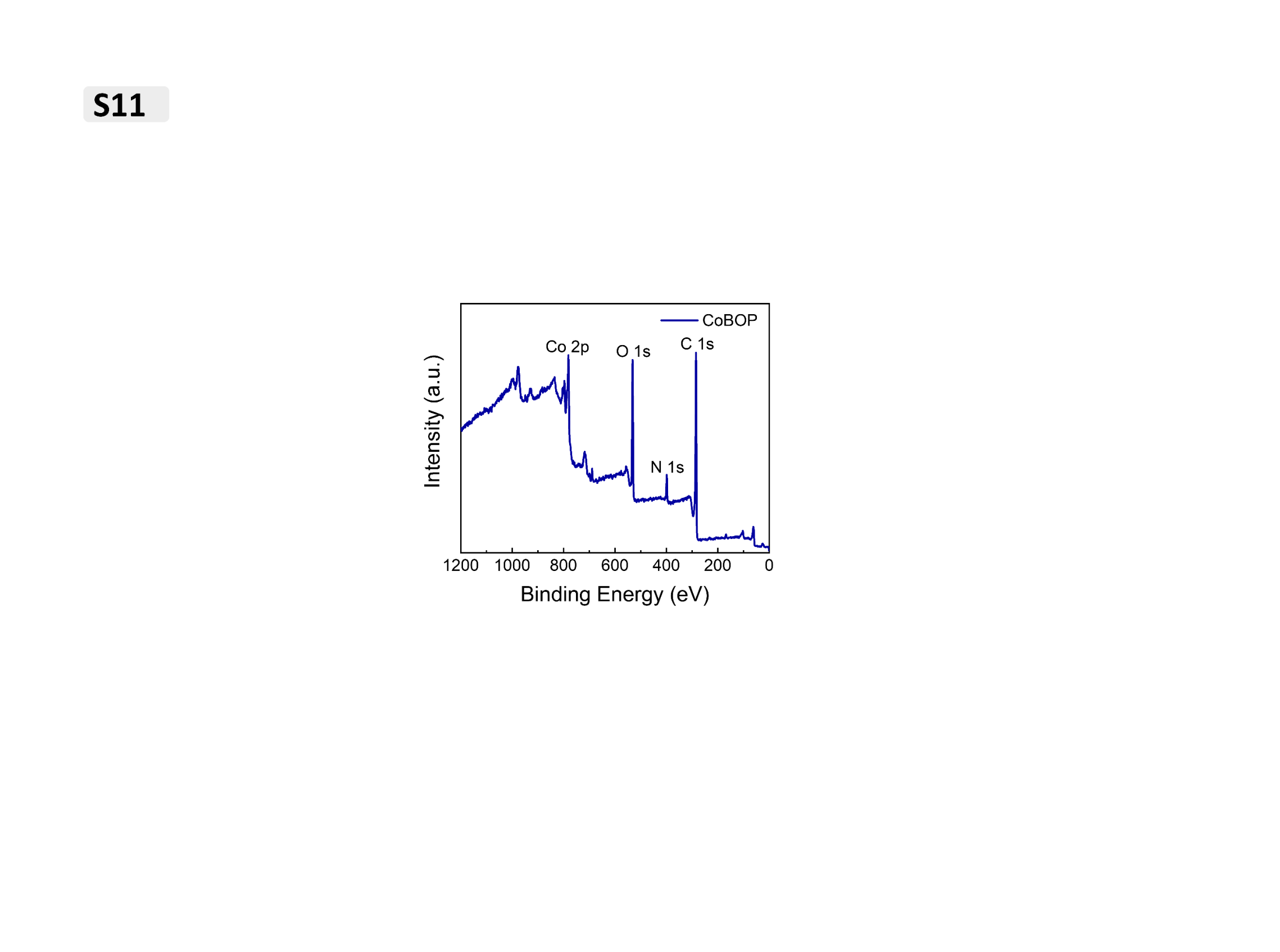


**Fig. S11** XPS spectra of CoBOP


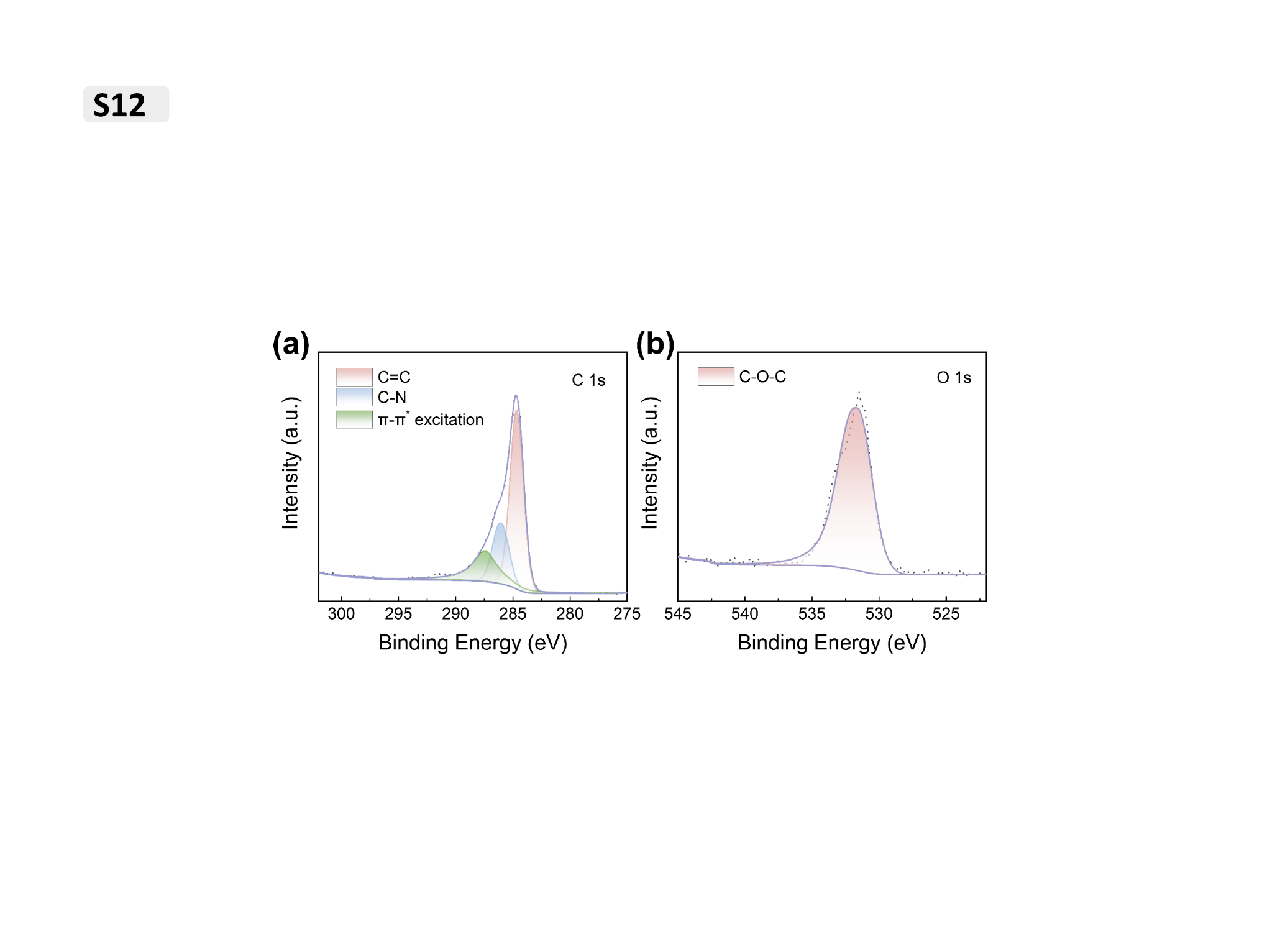


**Fig. S12** **a** C 1s XPS spectra of CoBOP. **b** O 1s XPS spectra of CoBOP


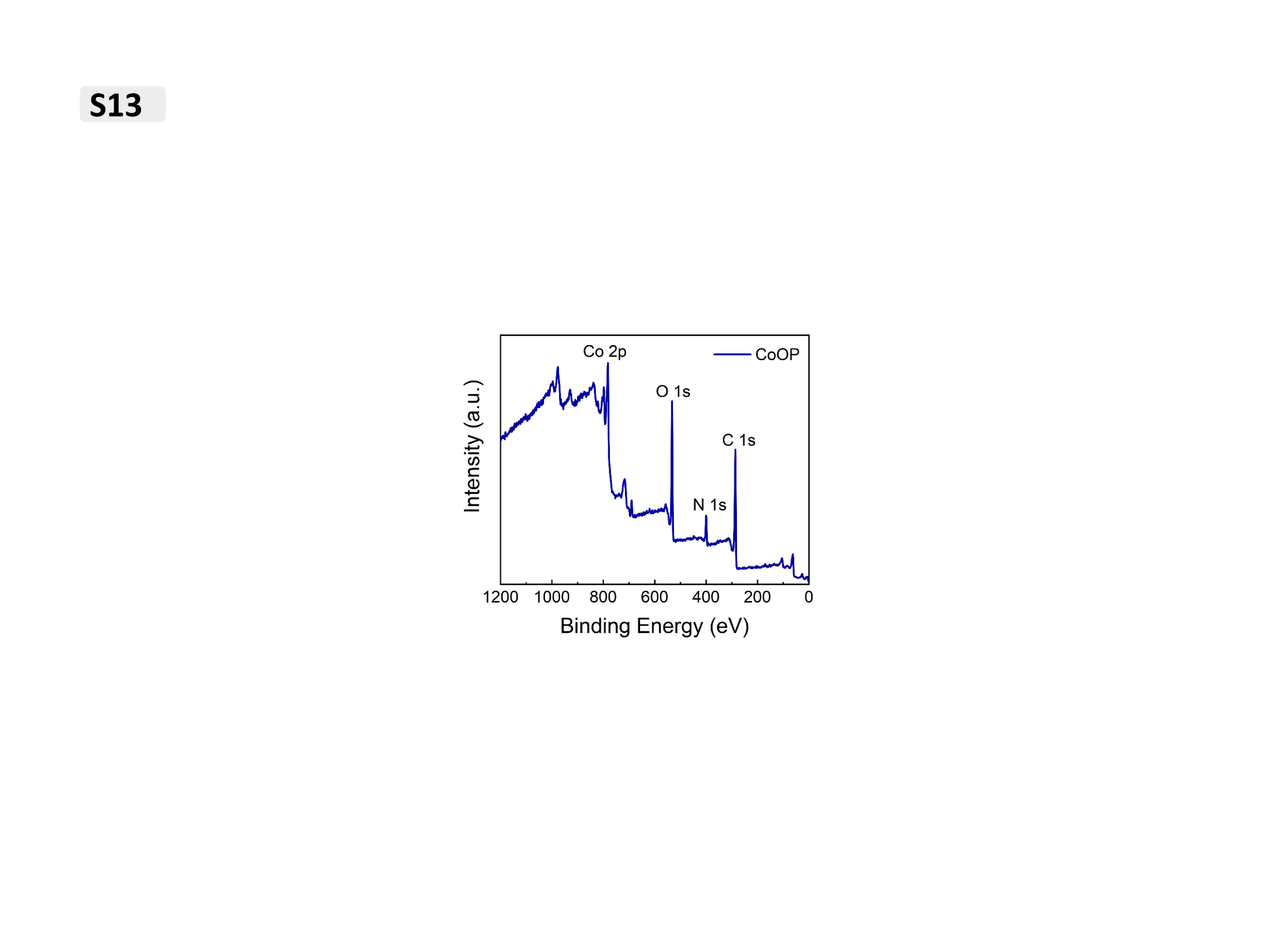


**Fig. S13** XPS spectra of CoOP


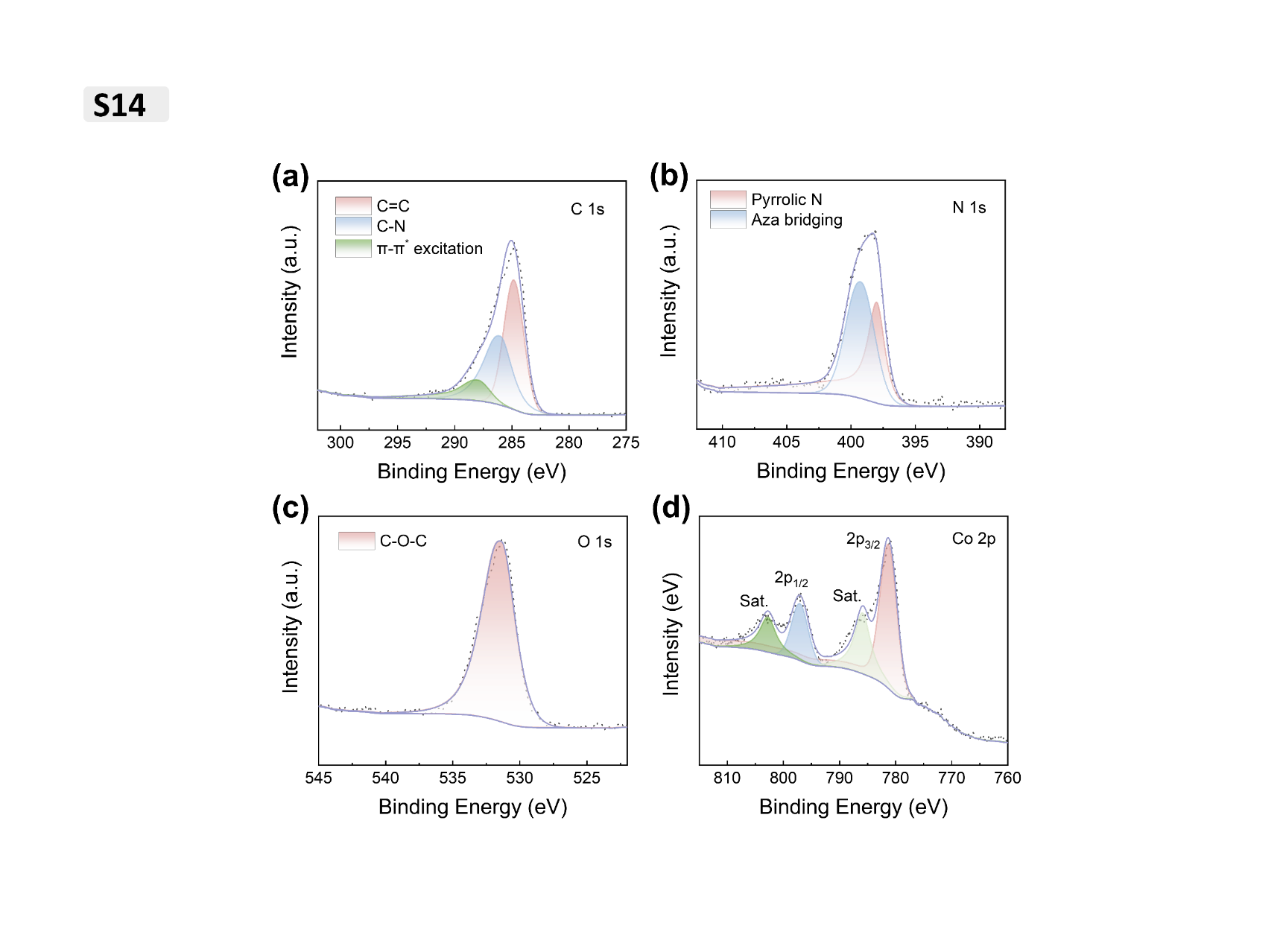


**Fig. S14** **a** C 1s XPS spectra of CoOP. **b** N 1s XPS spectra of CoOP. **c** O 1s XPS spectra of CoOP. **d** Co 2p XPS spectra of CoOP


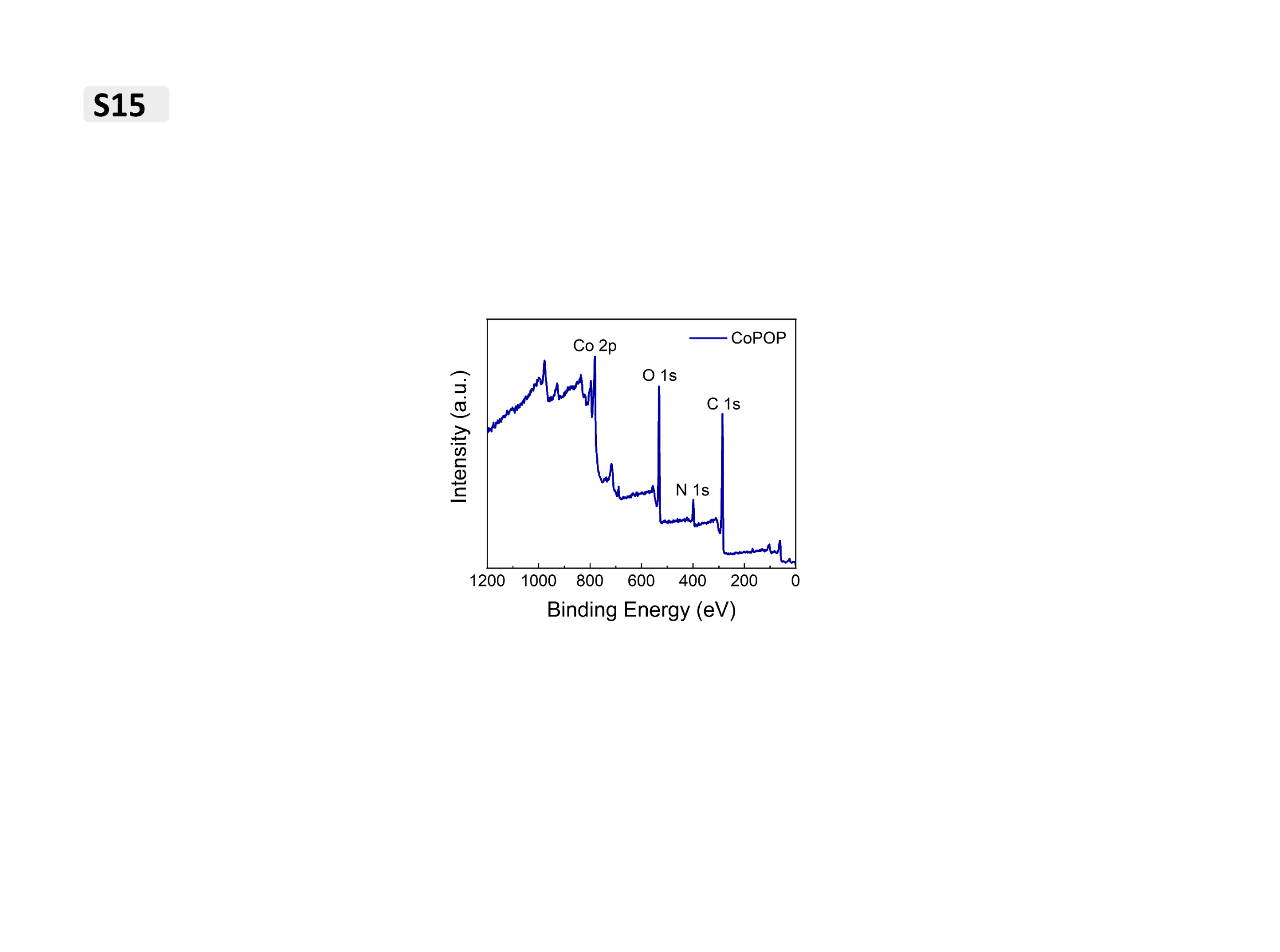


**Fig. S15** XPS spectra of CoPOP


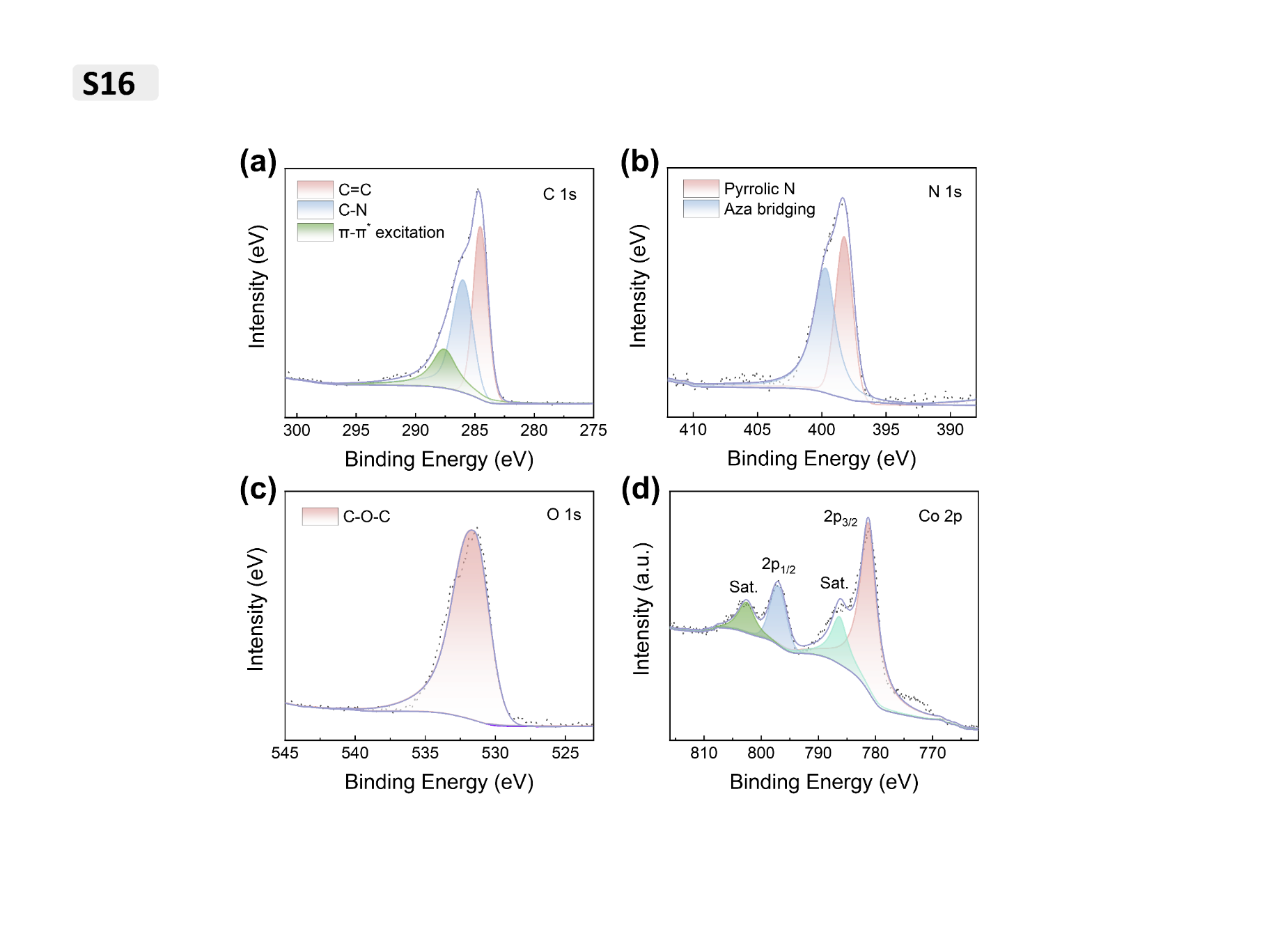


**Fig. S16** **a** C 1s XPS spectra of CoPOP. **b** N 1s XPS spectra of CoPOP. **c** O 1s XPS spectra of CoPOP. **d** Co 2p XPS spectra of CoPOP


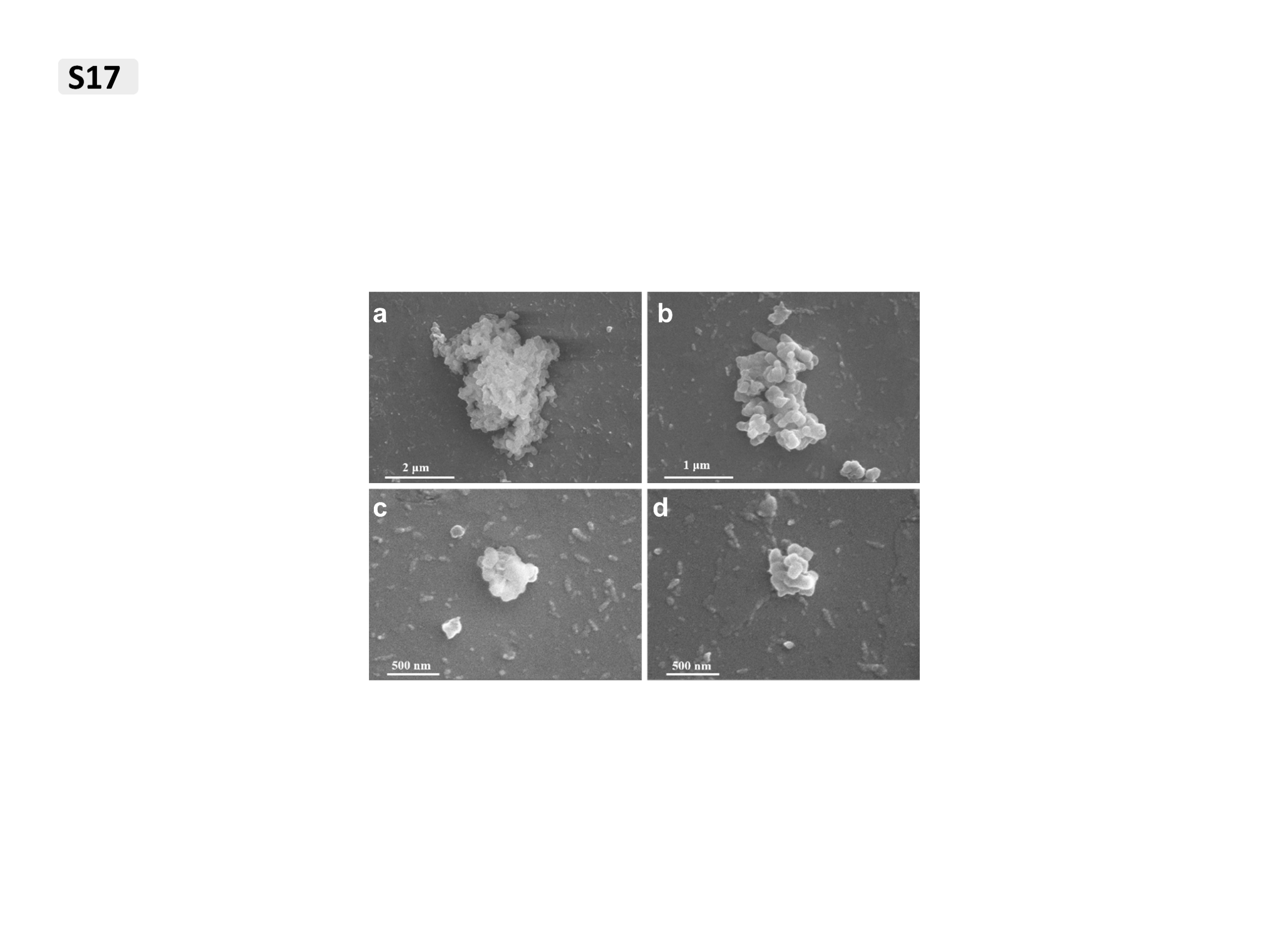


**Fig. S17 a** SEM images of CoOP with 2 μm. **b** SEM images of CoOP with 1 μm. **c**, **d** SEM images of CoOP with 500 nm


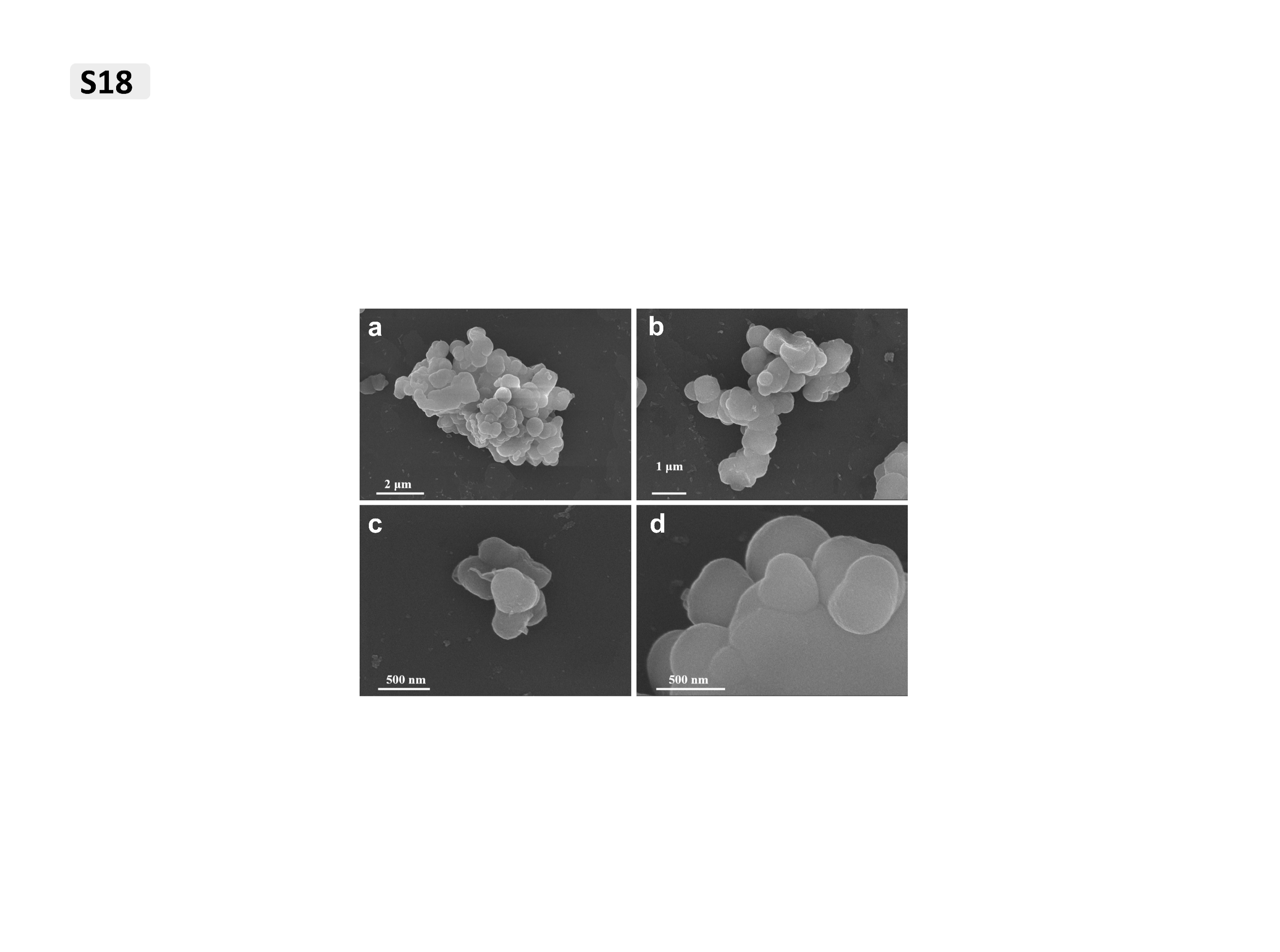


**Fig. S18** **a** SEM images of CoPOP with 2 μm. **b** SEM images of CoPOP with 1 μm. **c, d** SEM images of CoPOP with 500 nm


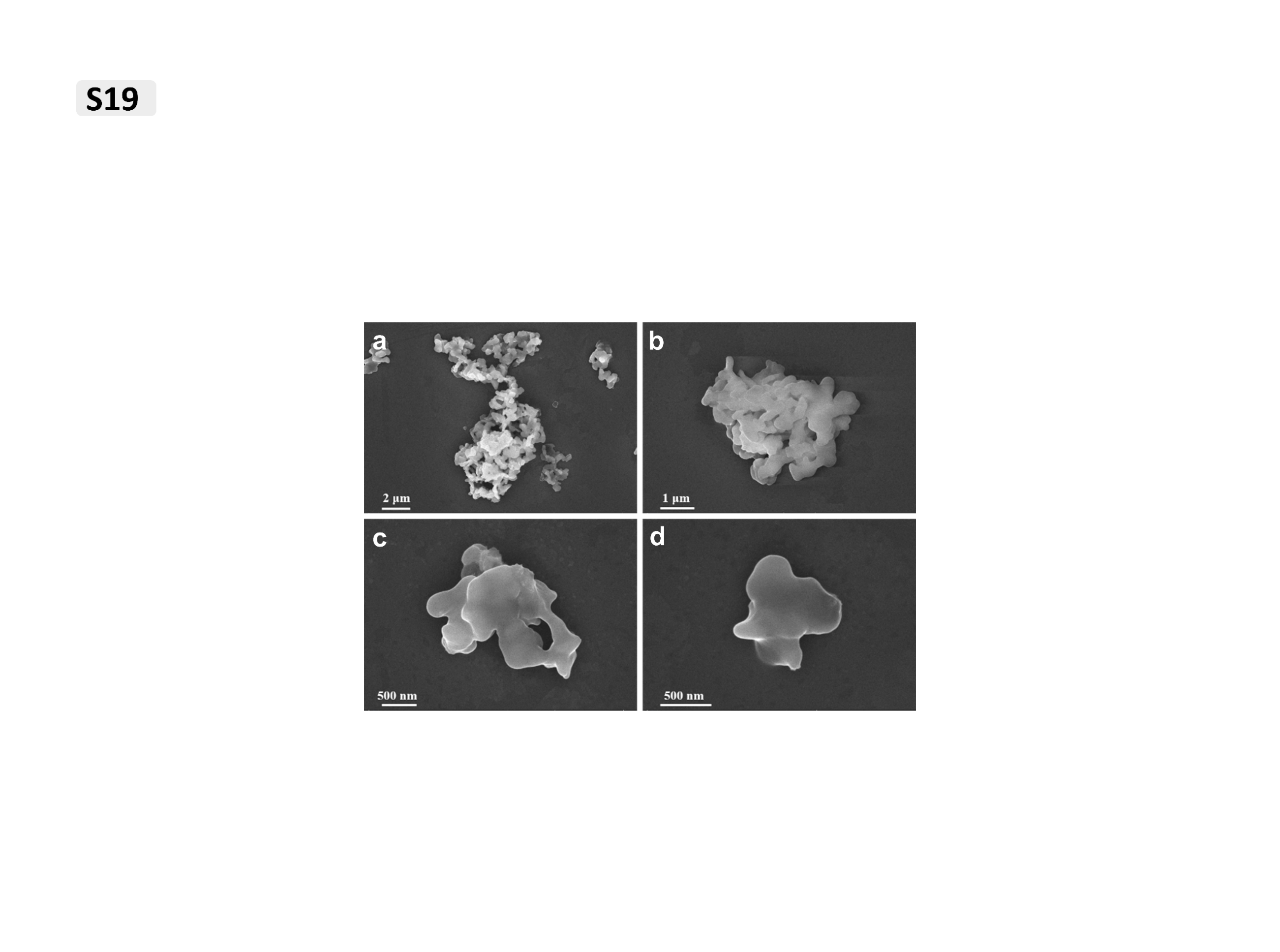


**Fig. S19** **a** SEM images of CoBOP with 2 μm. **b** SEM images of CoBOP with 1 μm. **c, d** SEM images of CoBOP with 500 nm


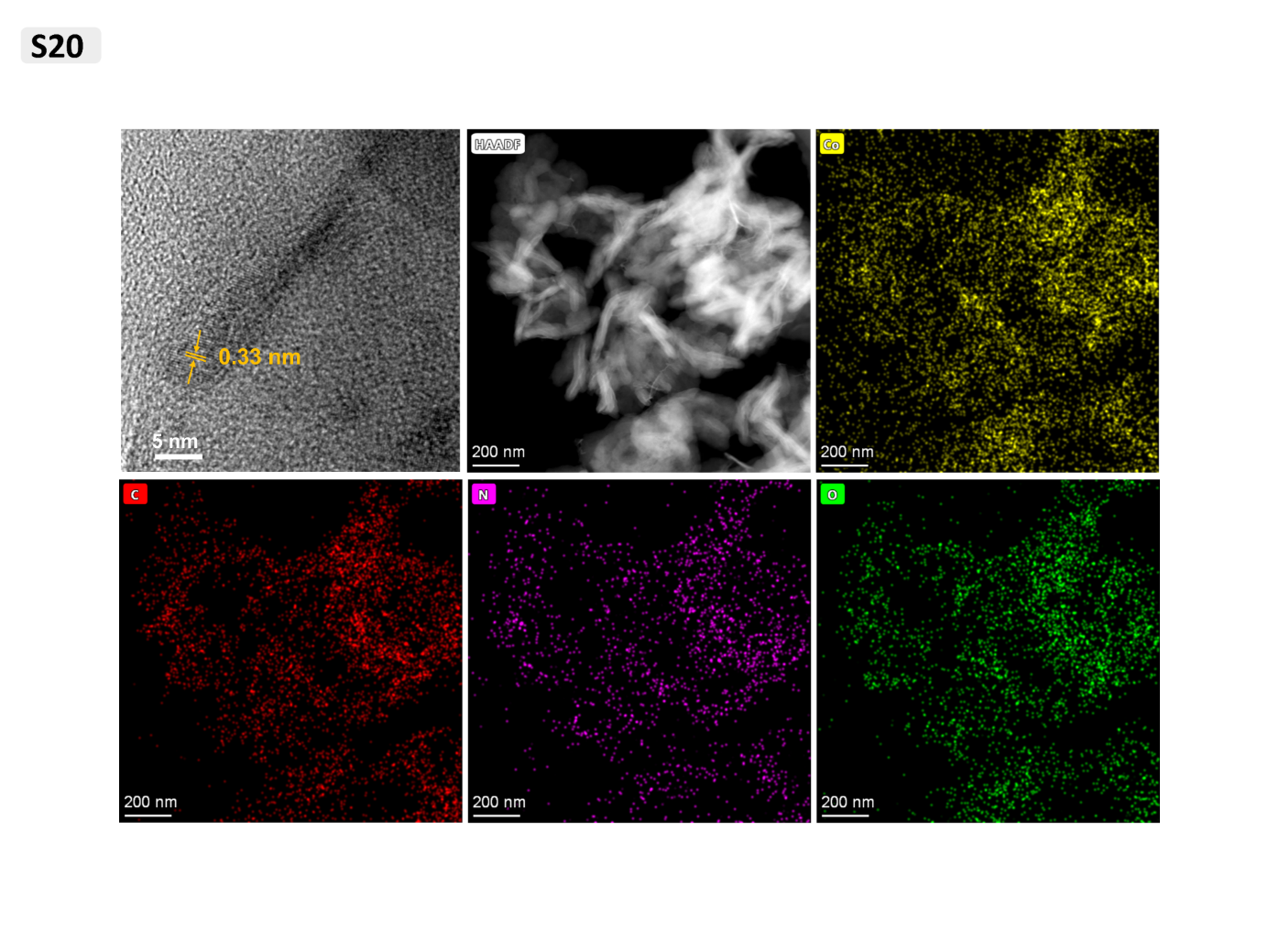


**Fig. S20** **a** TEM images of CoOP. **b** EDS mapping pictures of CoOP


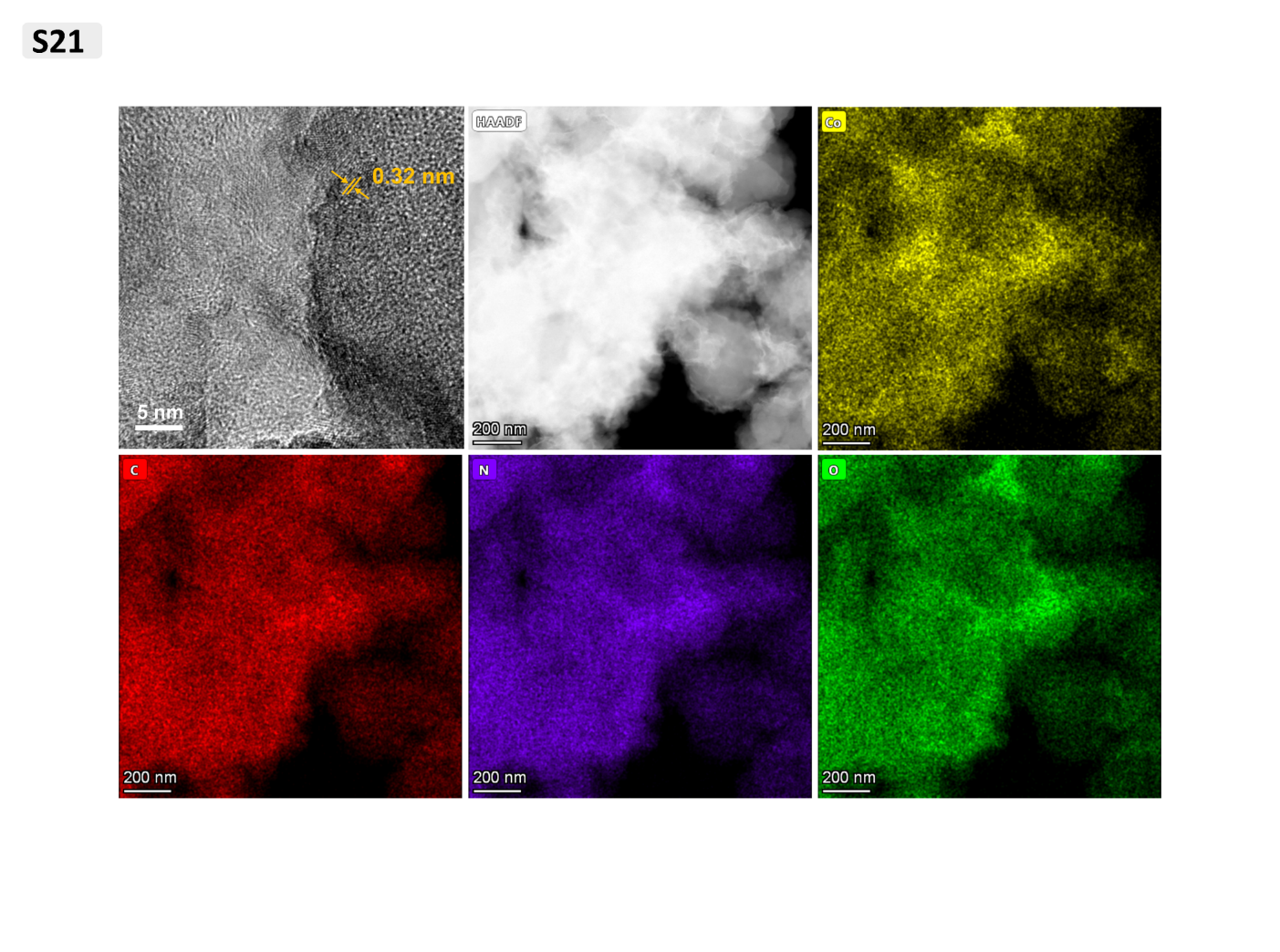


**Fig. S21** **a** TEM images of CoPOP. **b** EDS mapping pictures of CoPOP

**
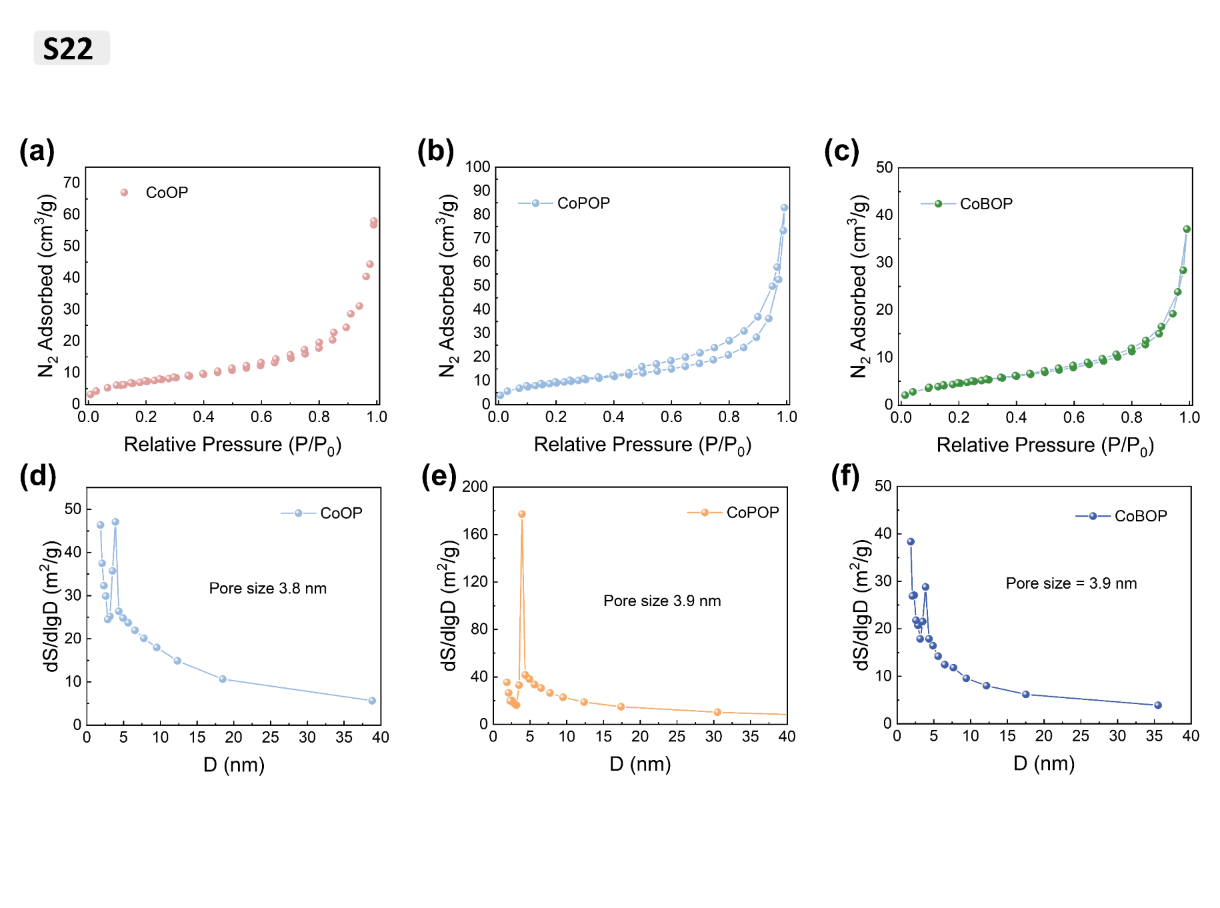
**

**Fig. S22** Nitrogen adsorption and desorption isotherms of CoOP **a**, CoPOP **b**, CoBOP **c**. The pore size distribution of CoOP **d**, CoPOP **e**, and CoBOP **f** by BJH (desorption)


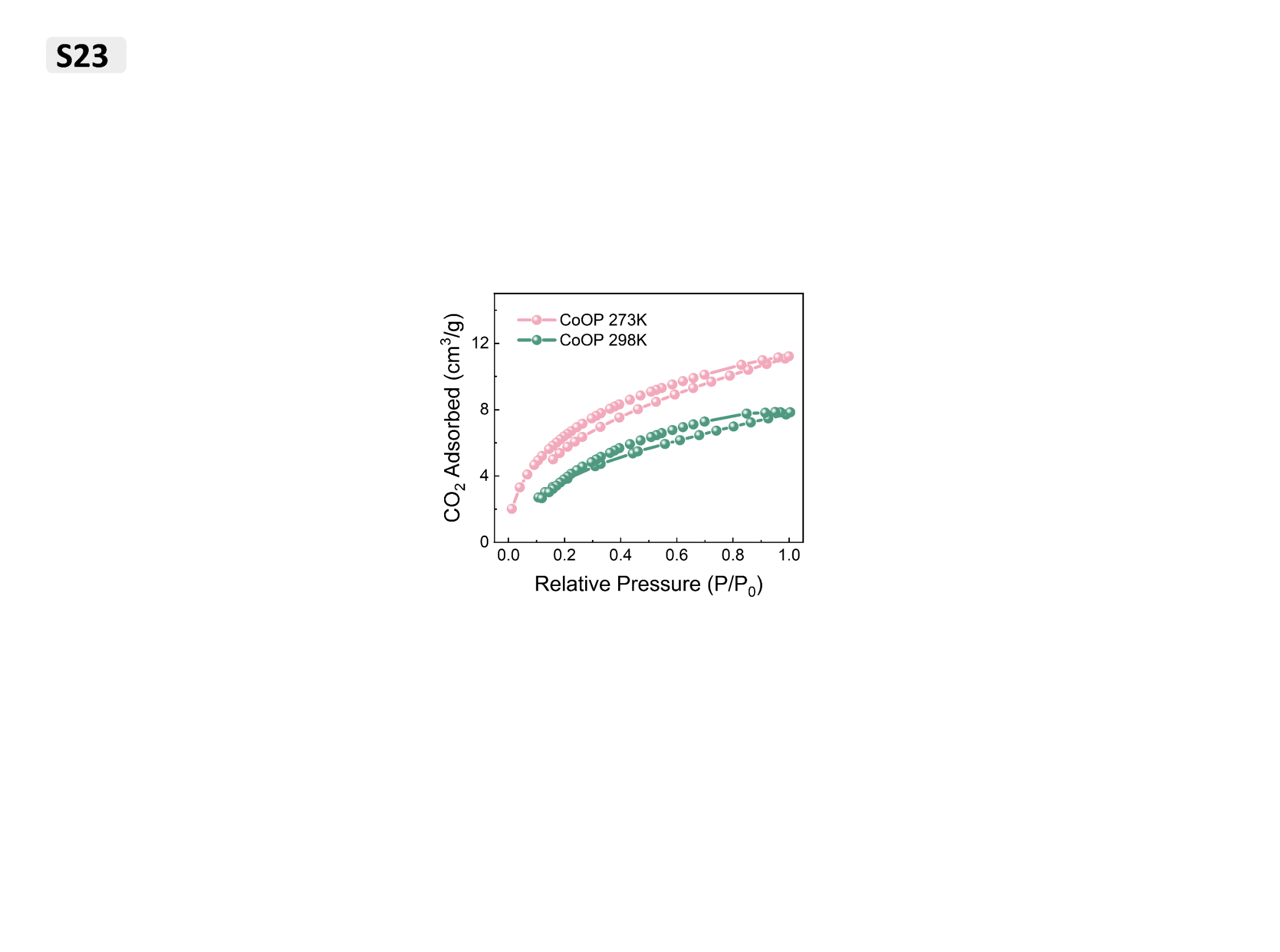


**Fig. S23** CO_2_ sorption isotherms in CoOP


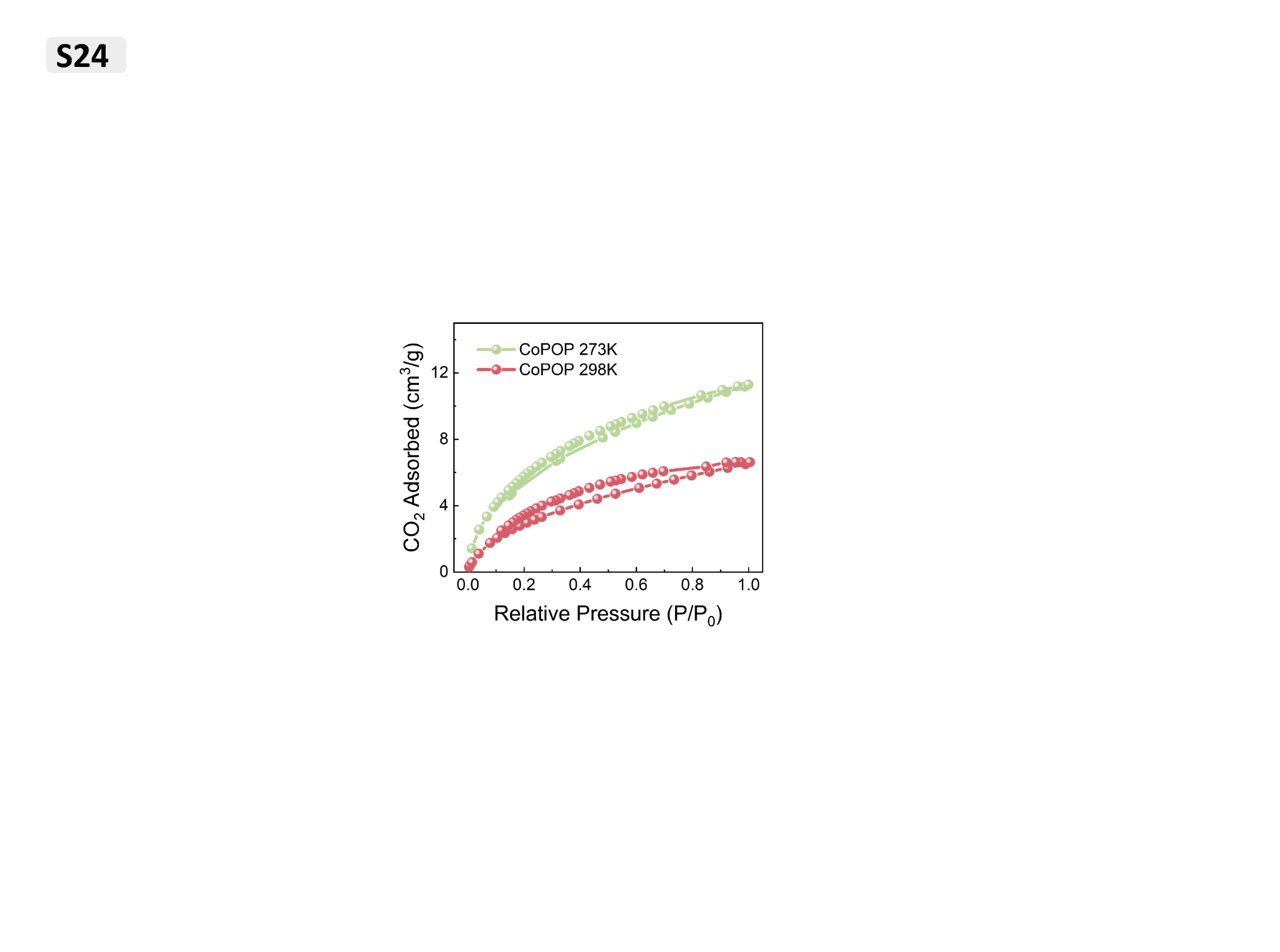


**Fig. S24** CO_2_ sorption isotherms in CoPOP


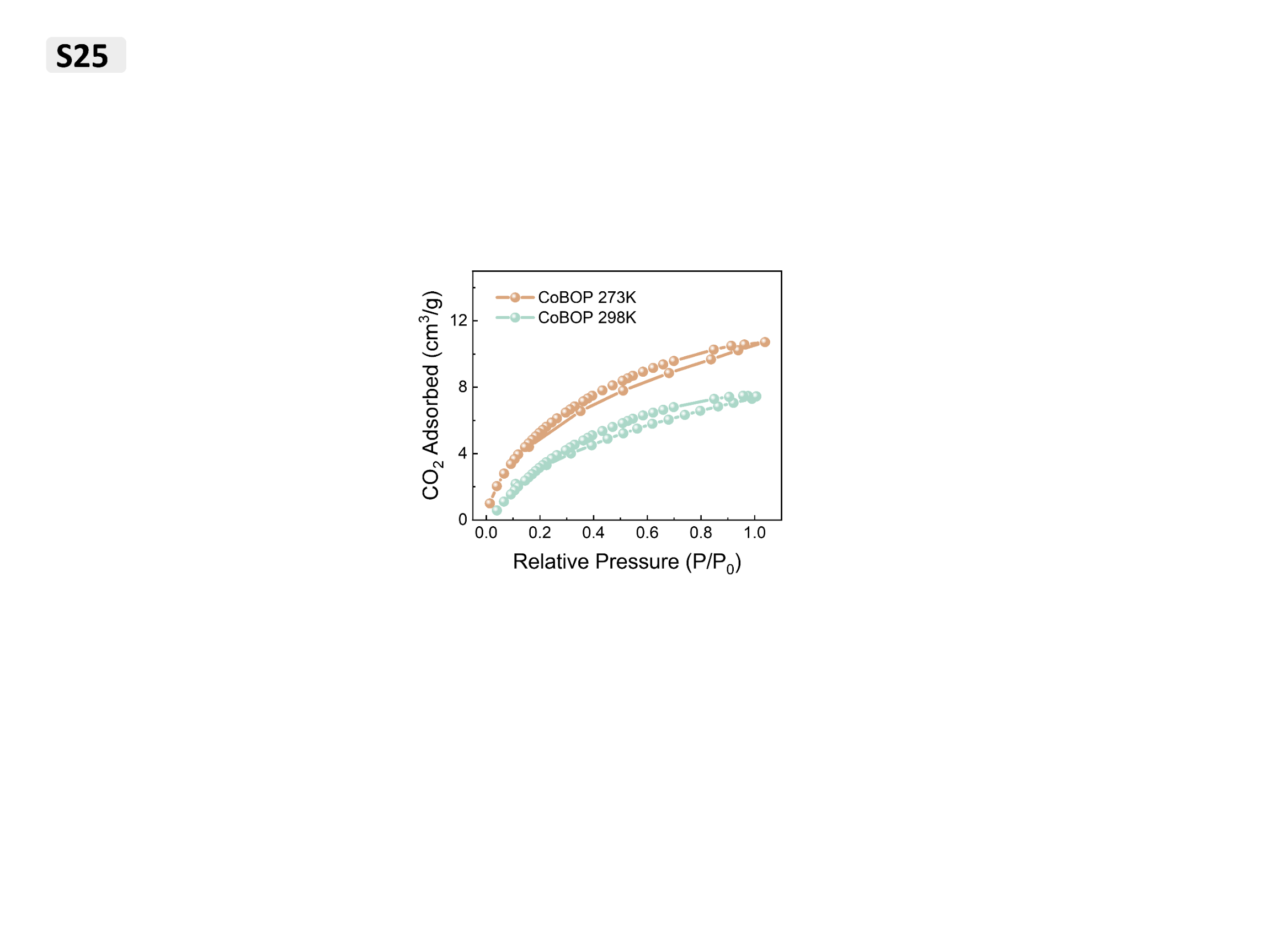


**Fig. S25** CO_2_ sorption isotherms in CoPOP


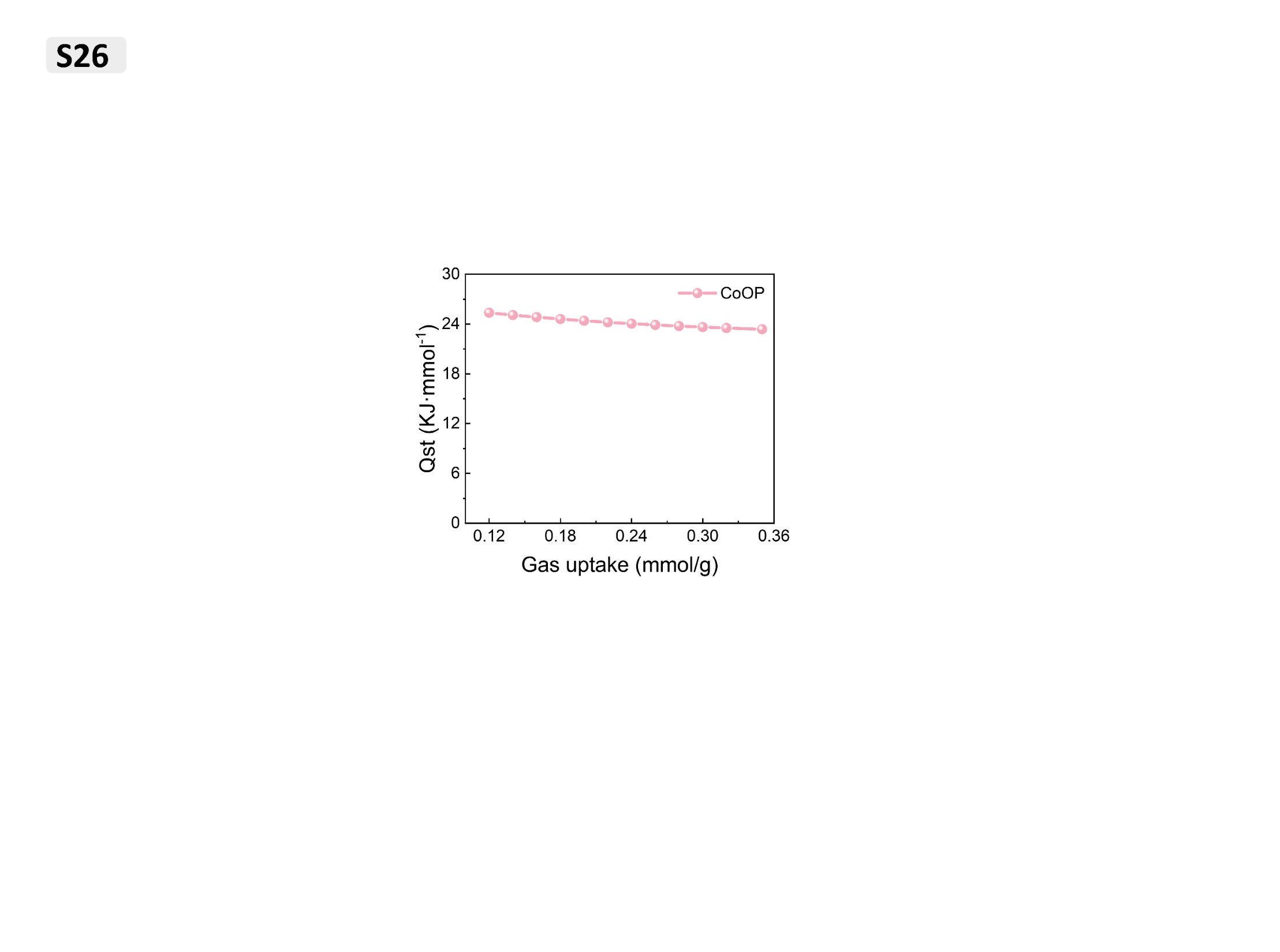


**Fig. S26** Q_st_ curve of CO_2_ of CoOP


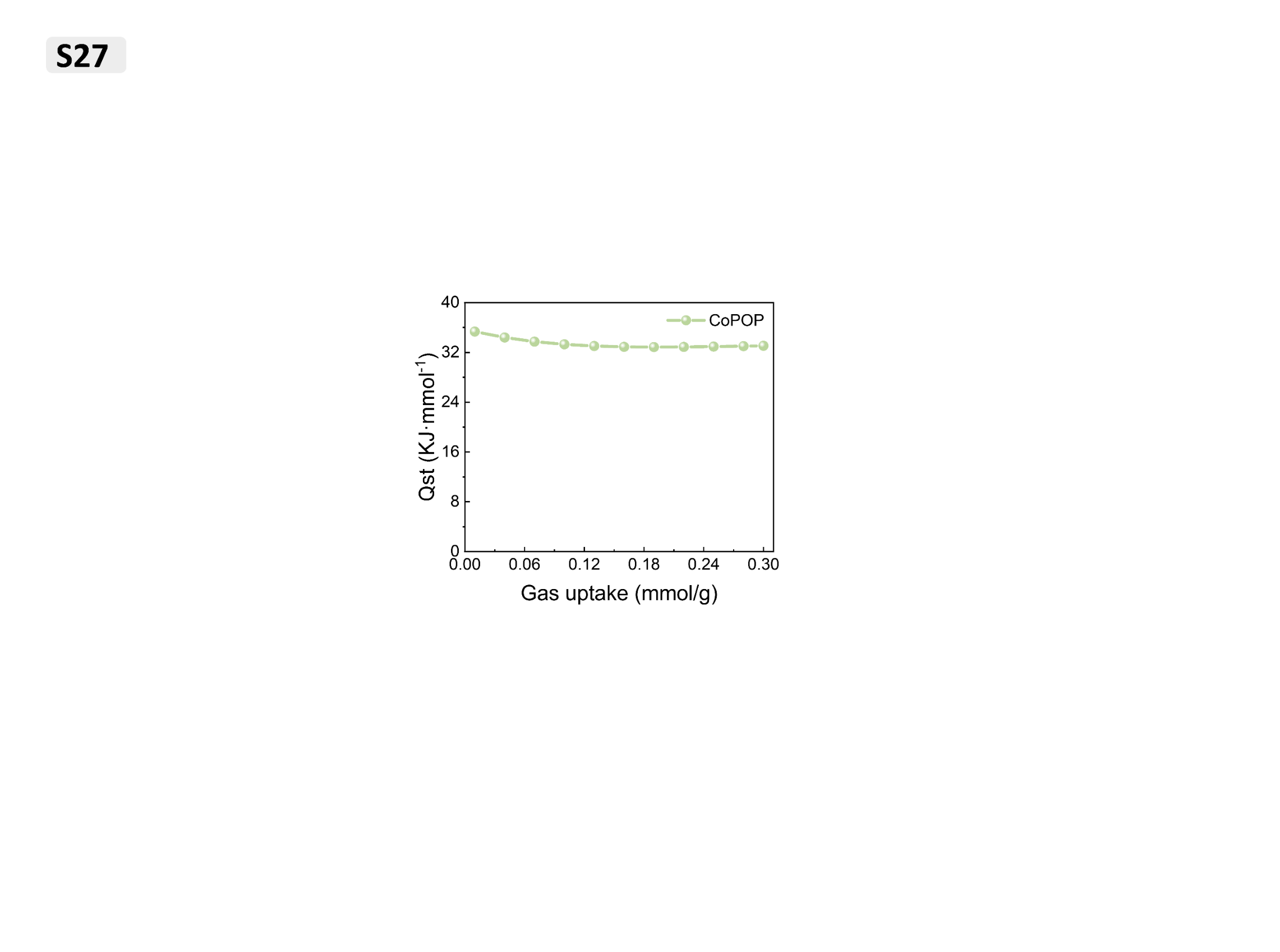


**Fig. S27** Q_st_ curve of CO_2_ of CoPOP


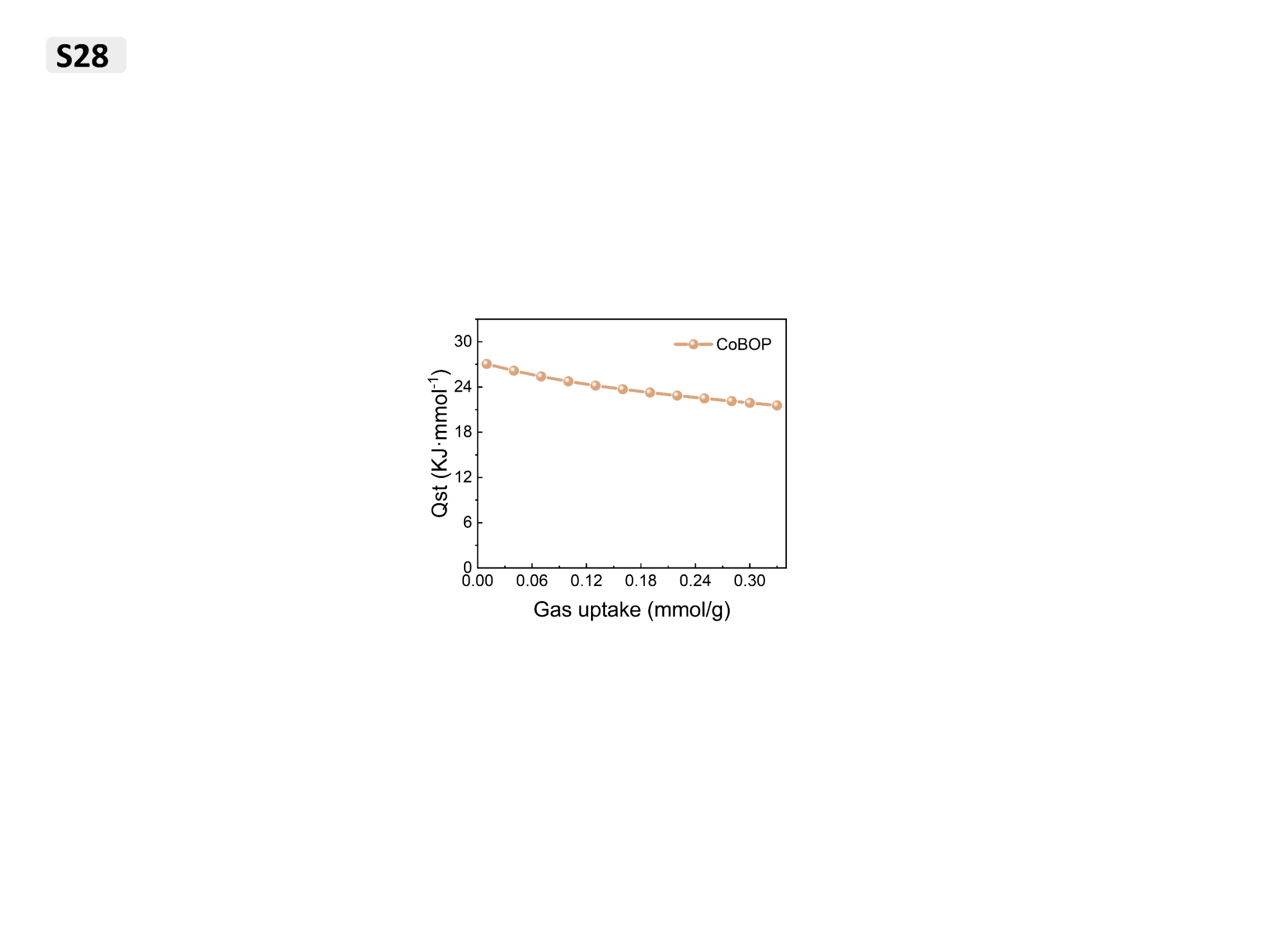


**Fig. S28** Q_st_ curve of CO_2_ of CoBOP


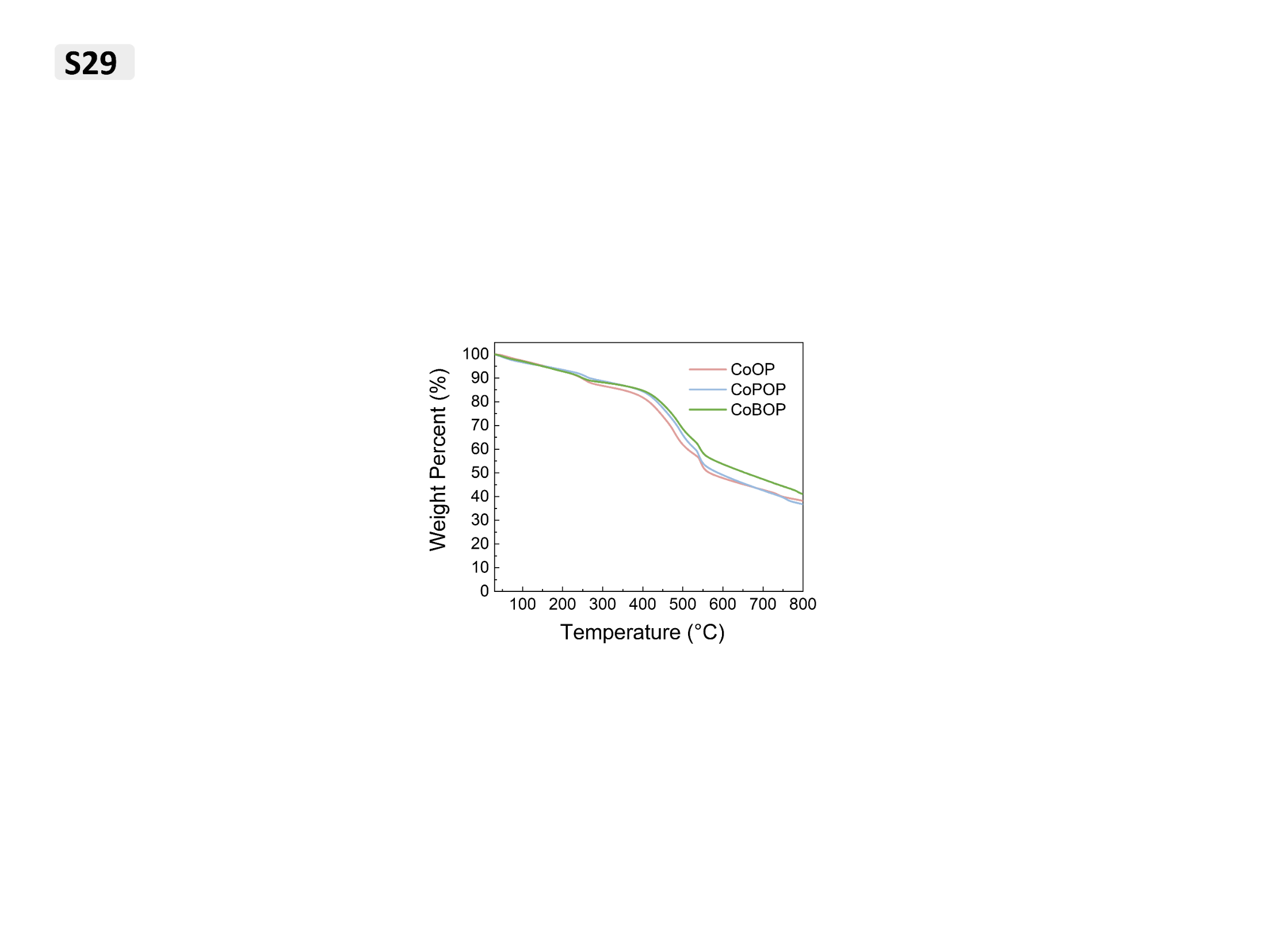


**Fig. S29** TGA spectra of CoOP, CoPOP, and CoBOP

**
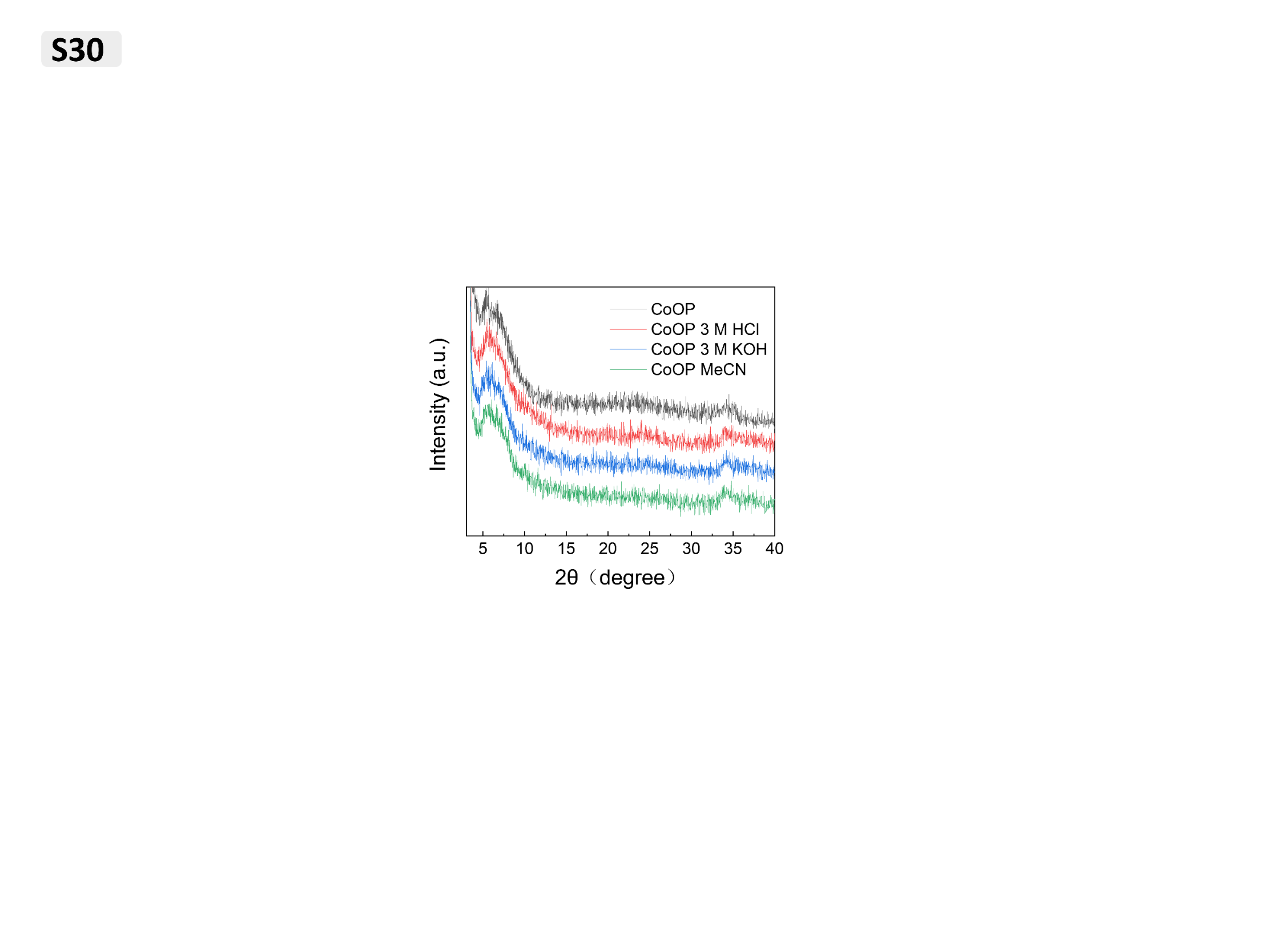
**

**Fig. S30** XRD spectra of CoOP after immersion in various solutions


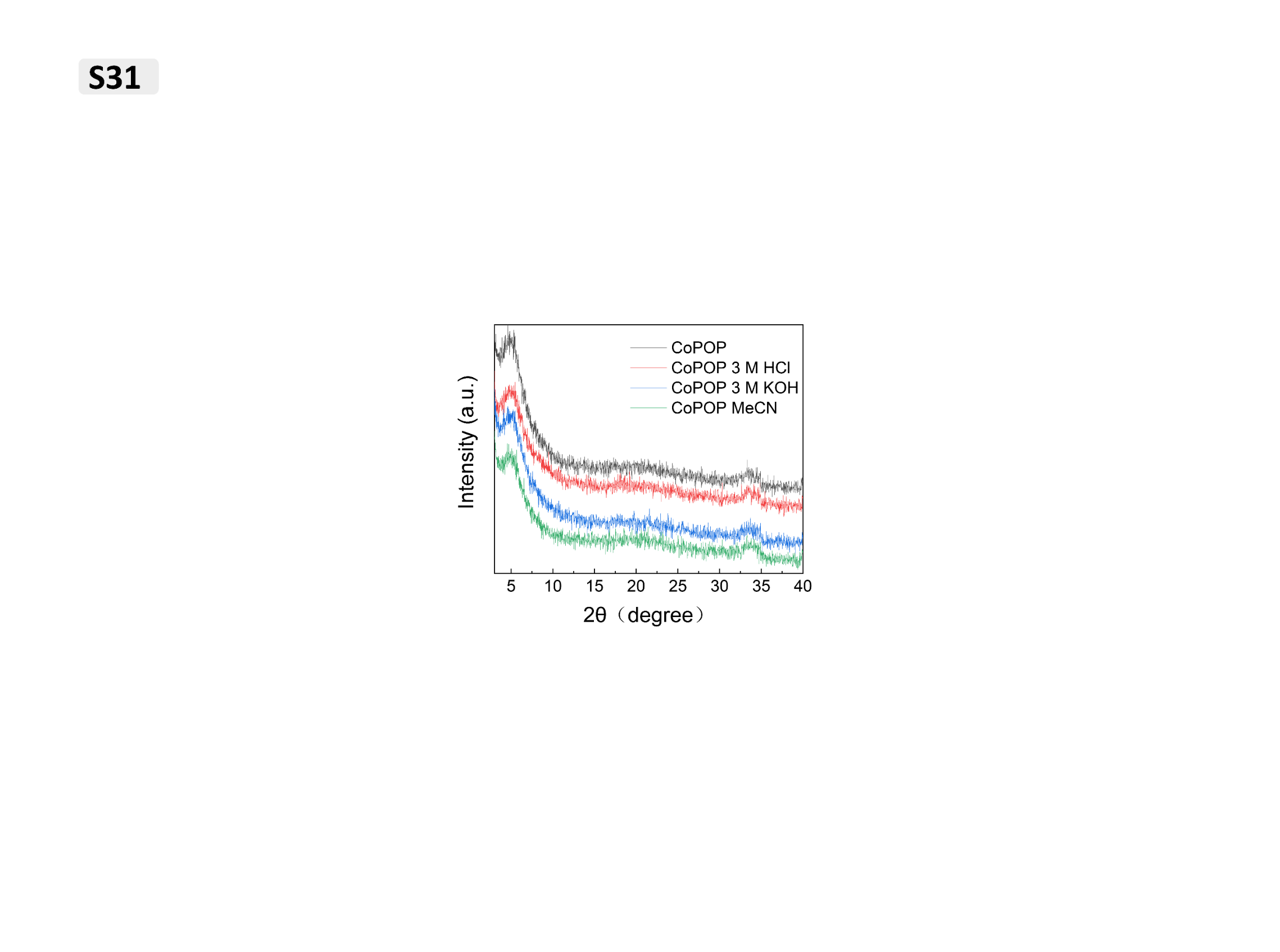


**Fig. S31** XRD spectra of CoPOP after immersion in various solutions


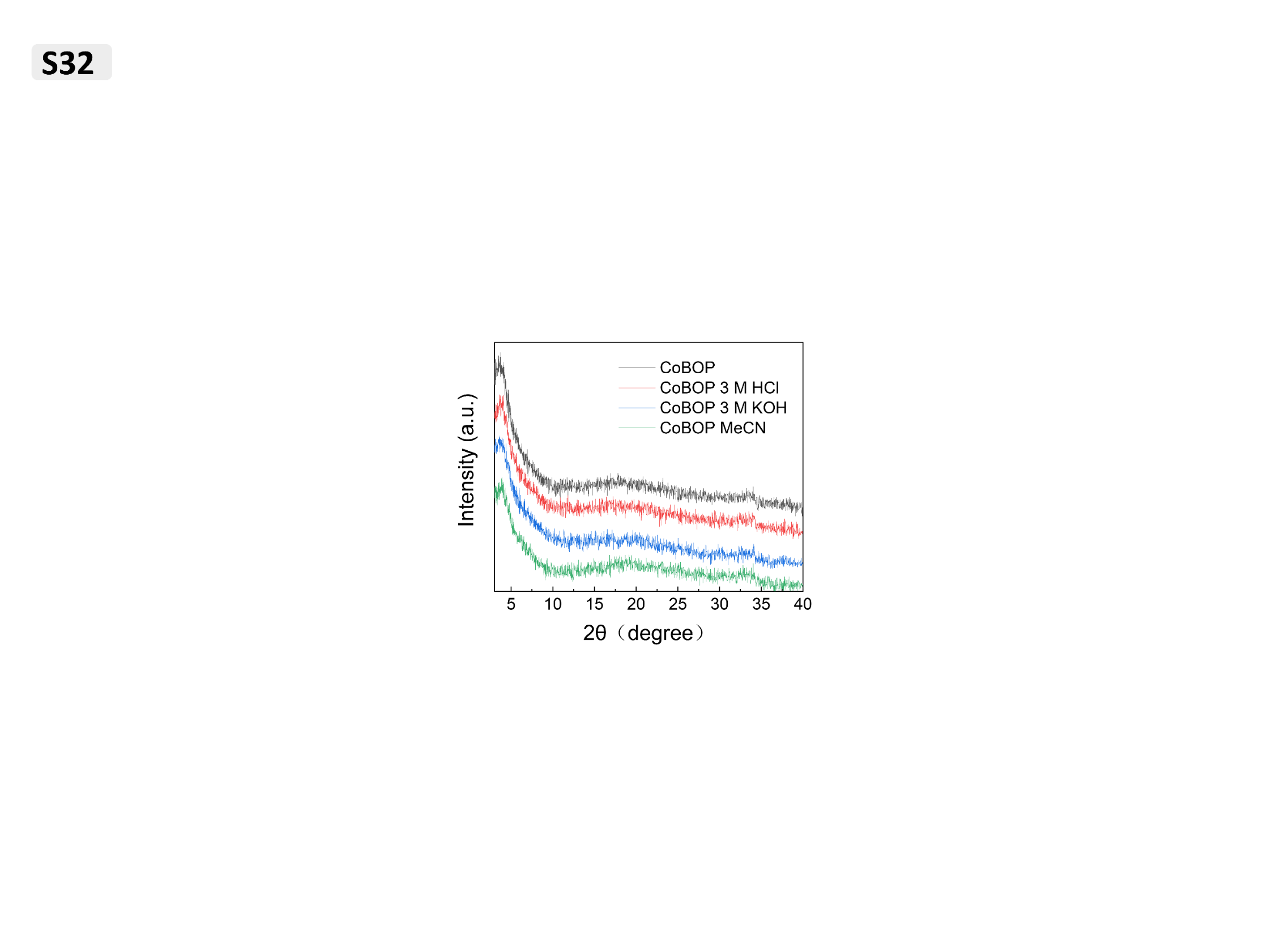


**Fig. S32** XRD spectra of CoBOP after immersion in various solutions


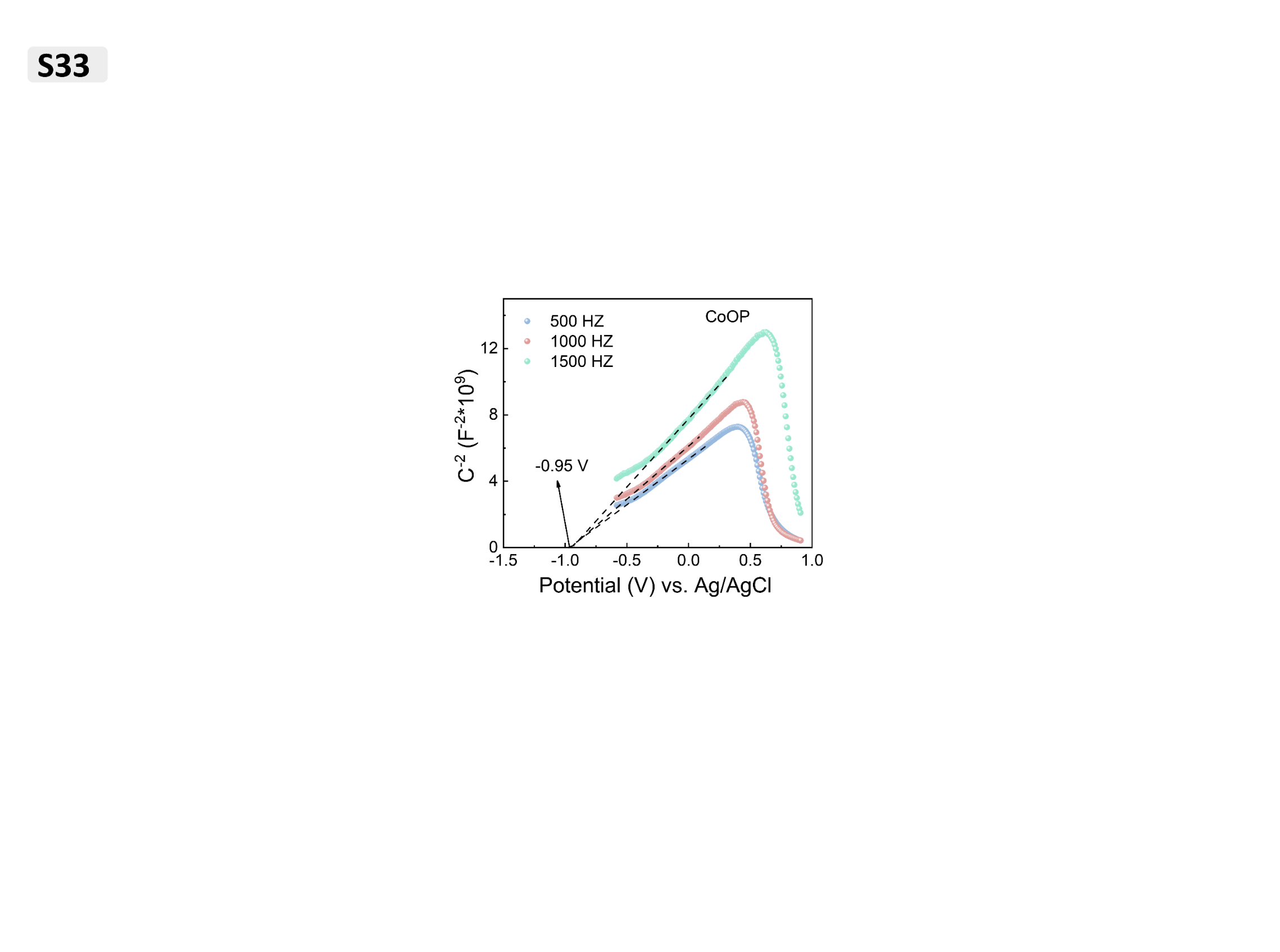


**Fig. S33** Mott-Schottky curves of CoOP at different frequencies


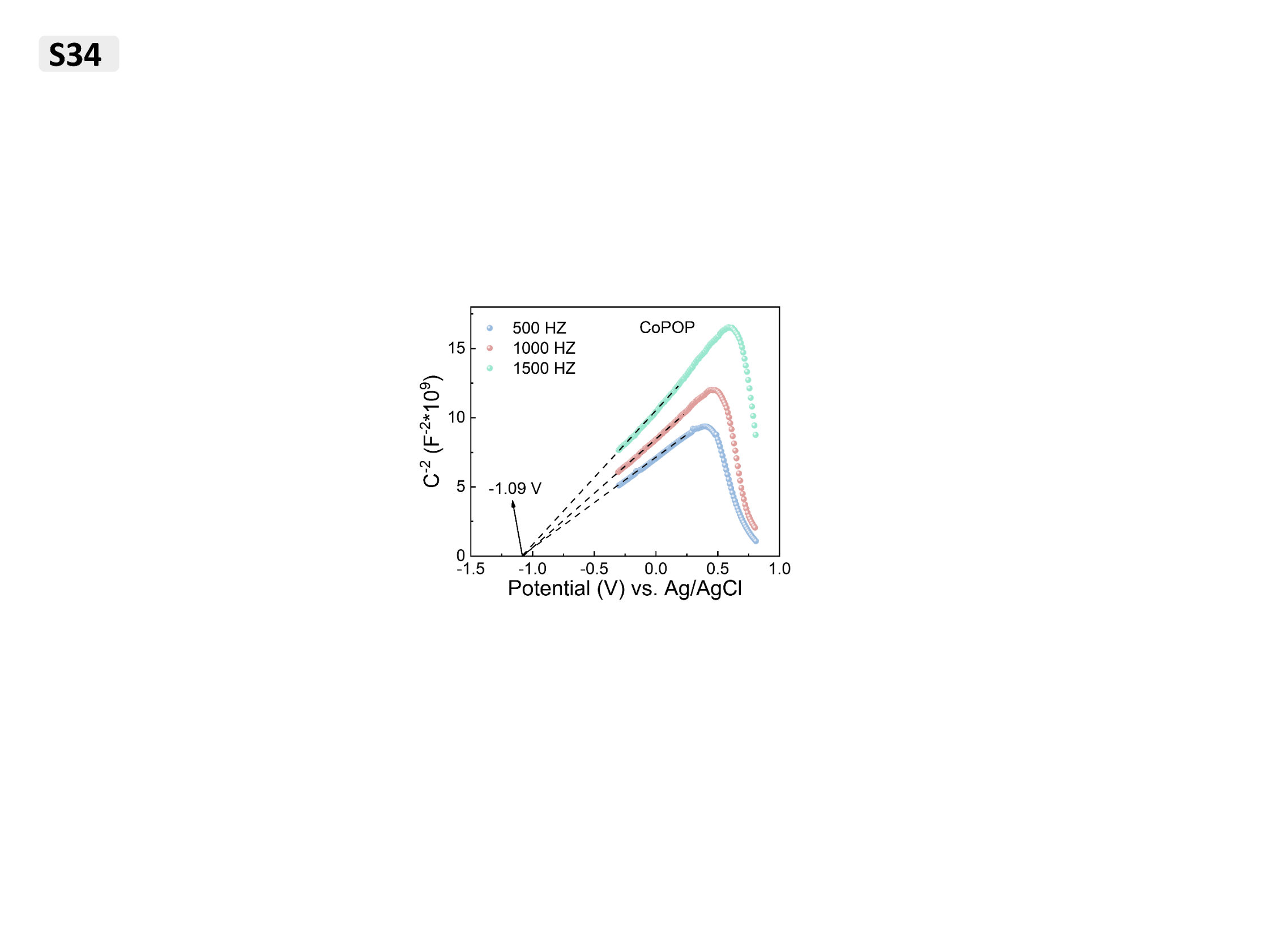


**Fig. S34** Mott-Schottky curves of CoPOP at different frequencies


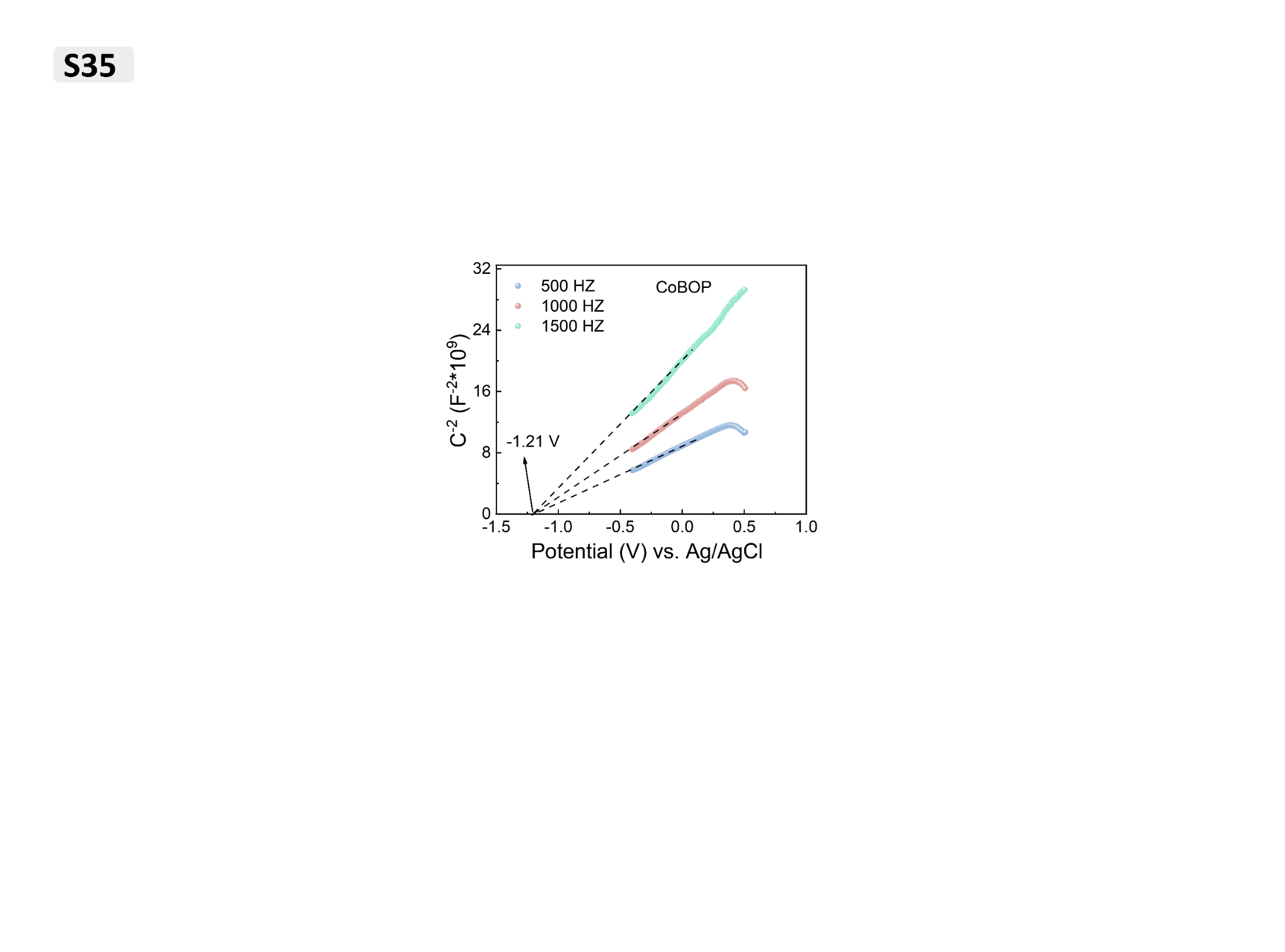


**Fig. S35** Mott-Schottky curves of CoBOP at different frequencies


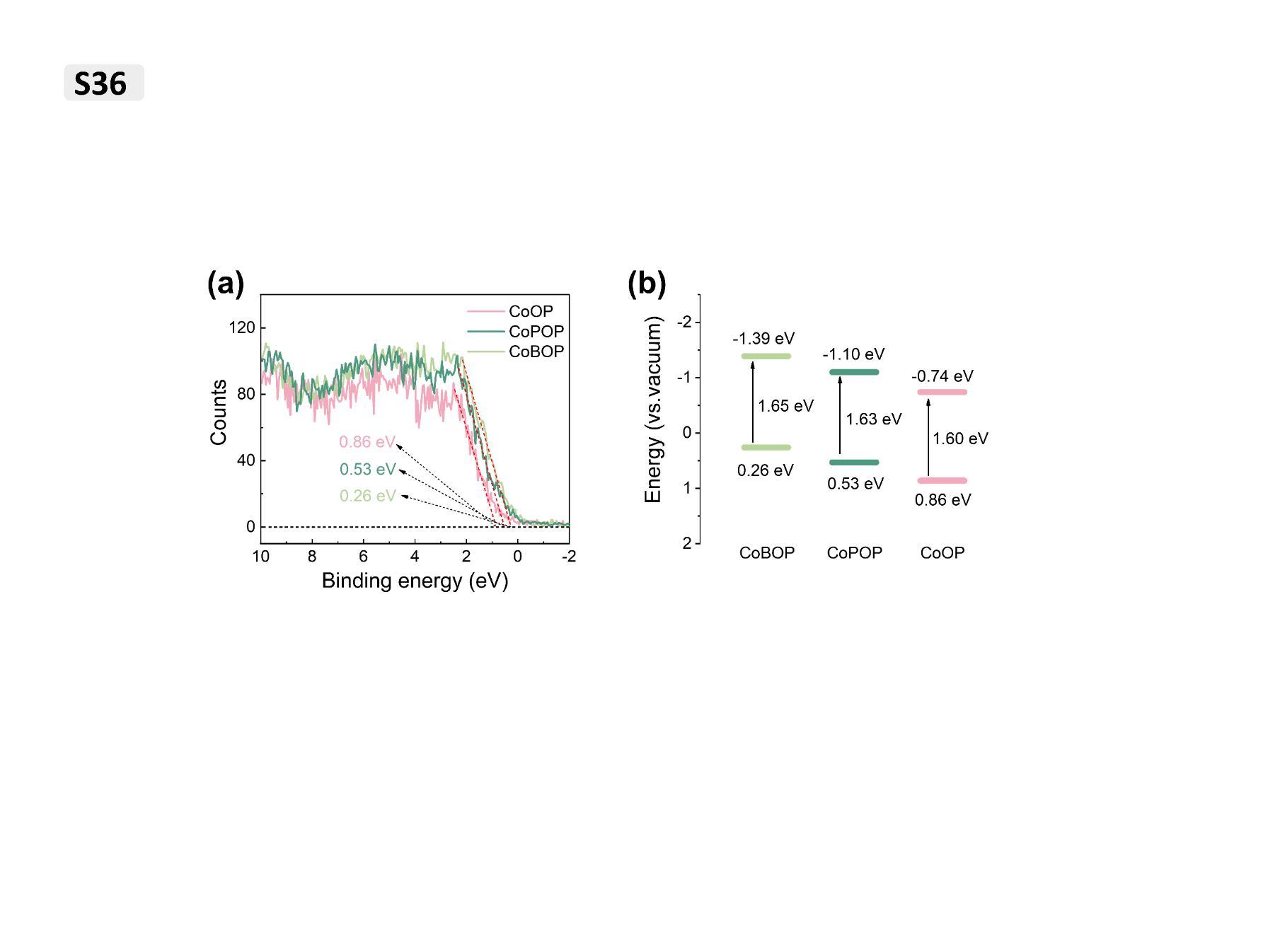


**Fig. S36** **a** Valence band XPS spectra of the CoOP, CoPOP, and CoBOP. **b** Band-structure (vs. vacuum) diagram based on UV-vis spectra and XPS-VB for CoOP, CoPOP, and CoBOP


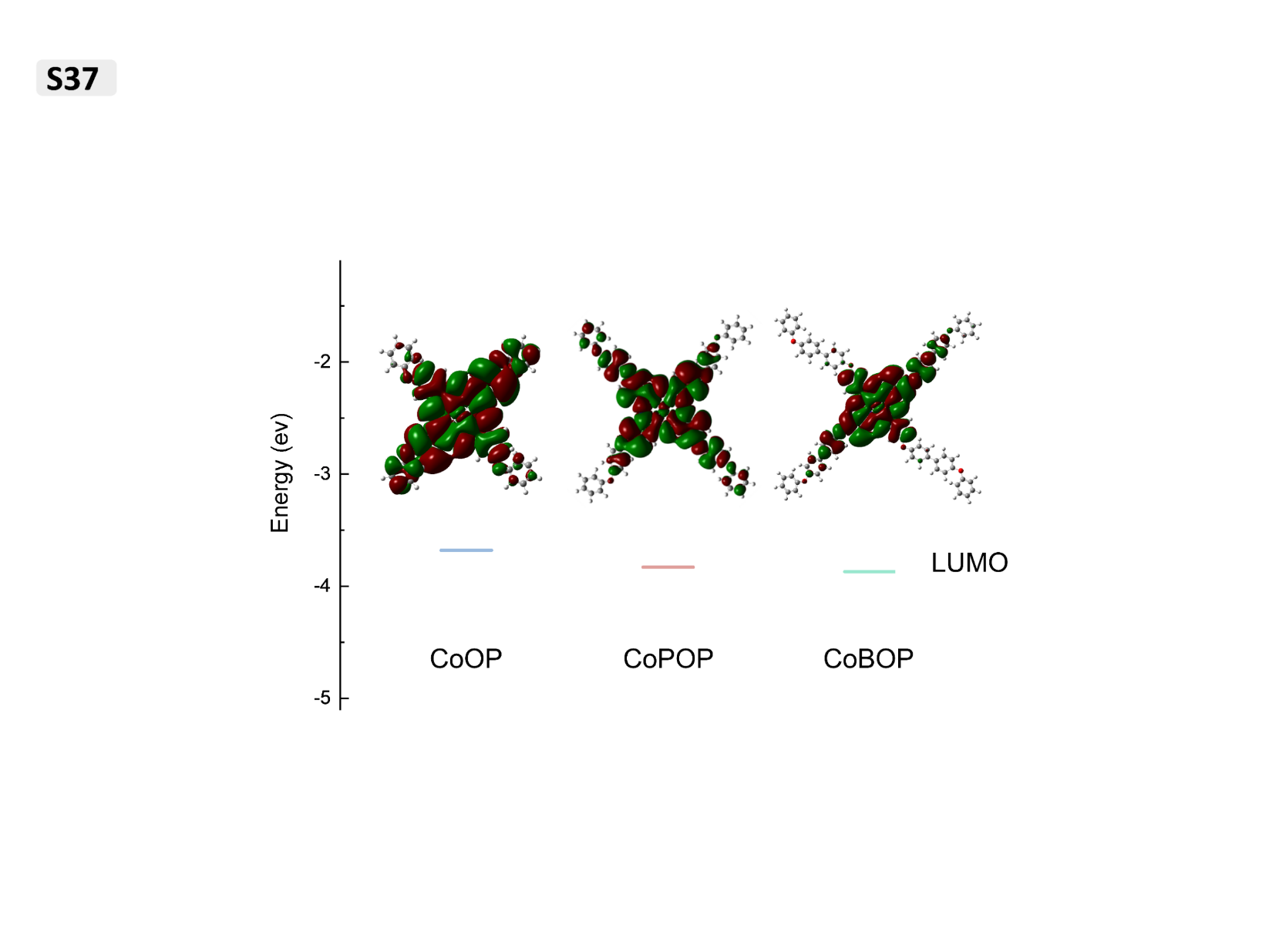


**Fig. S37** LUMO of CoOP、CoPOP and CoBOP


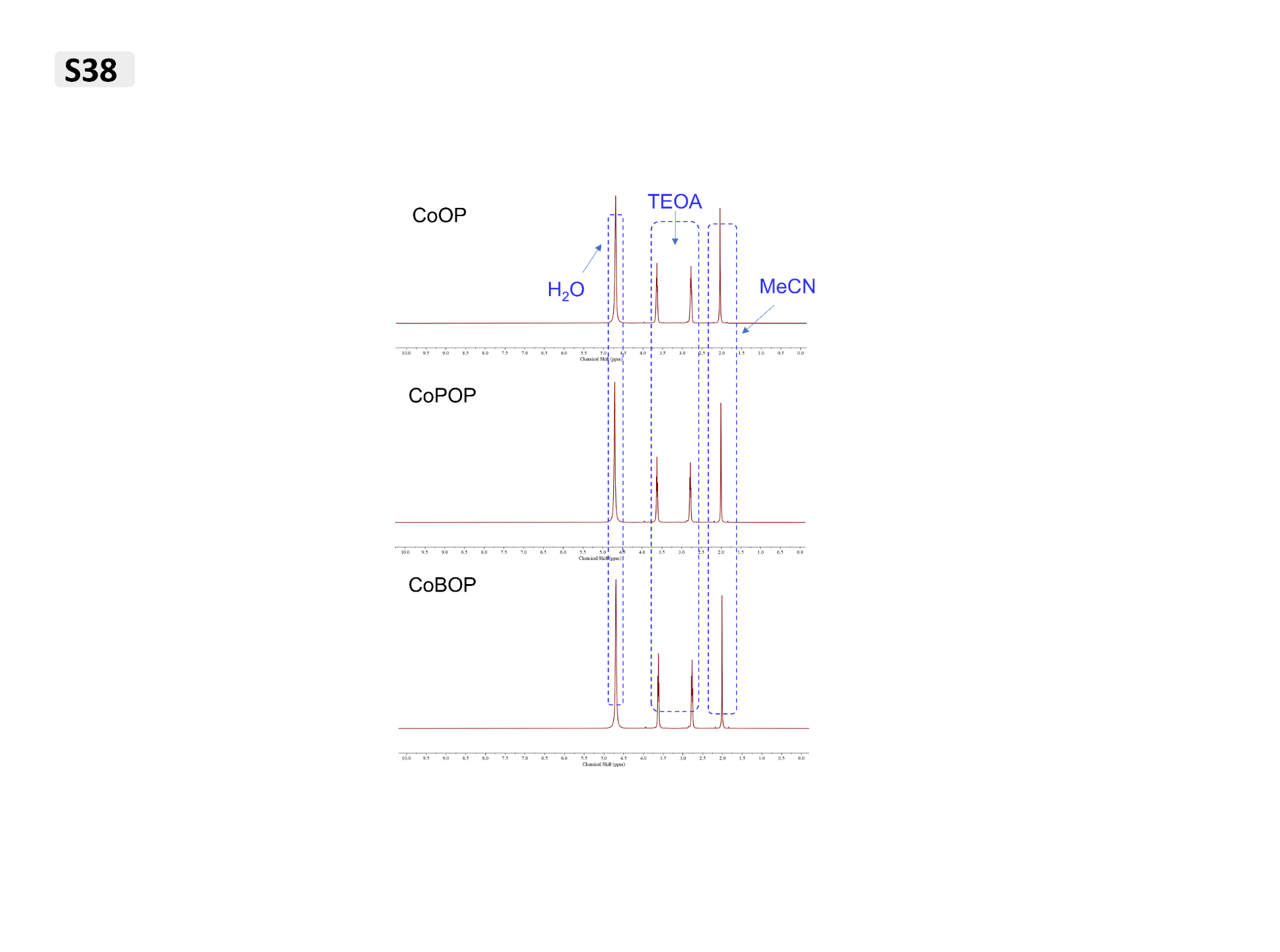


**Fig. S38** ^1^H NMR spectra of the liquid products resulting from the pCO_2_RR mediated by CoOP, CoPOP, and CoBOP


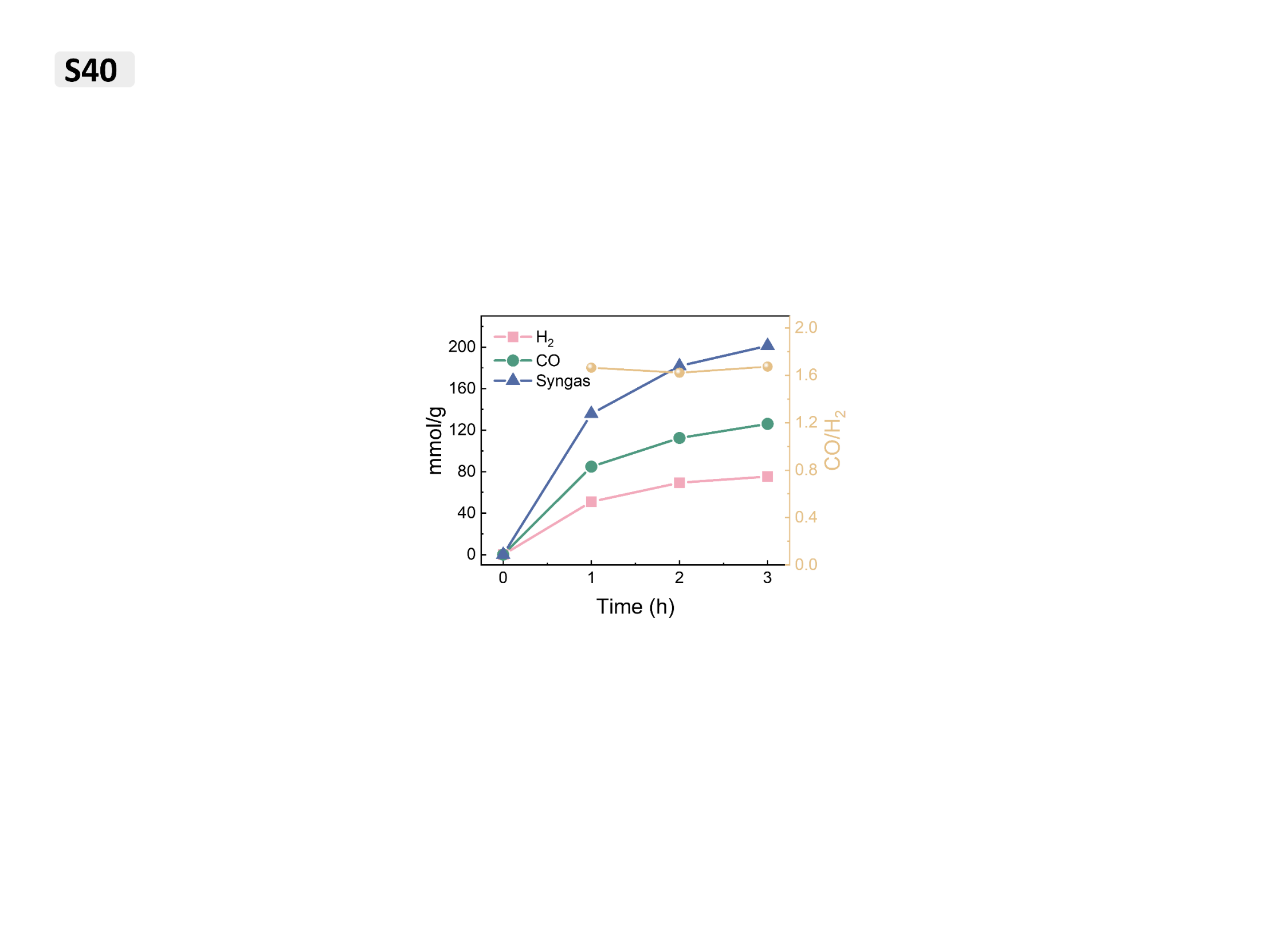


**Fig. S39** Time-dependent photocatalytic activity of CoBOP


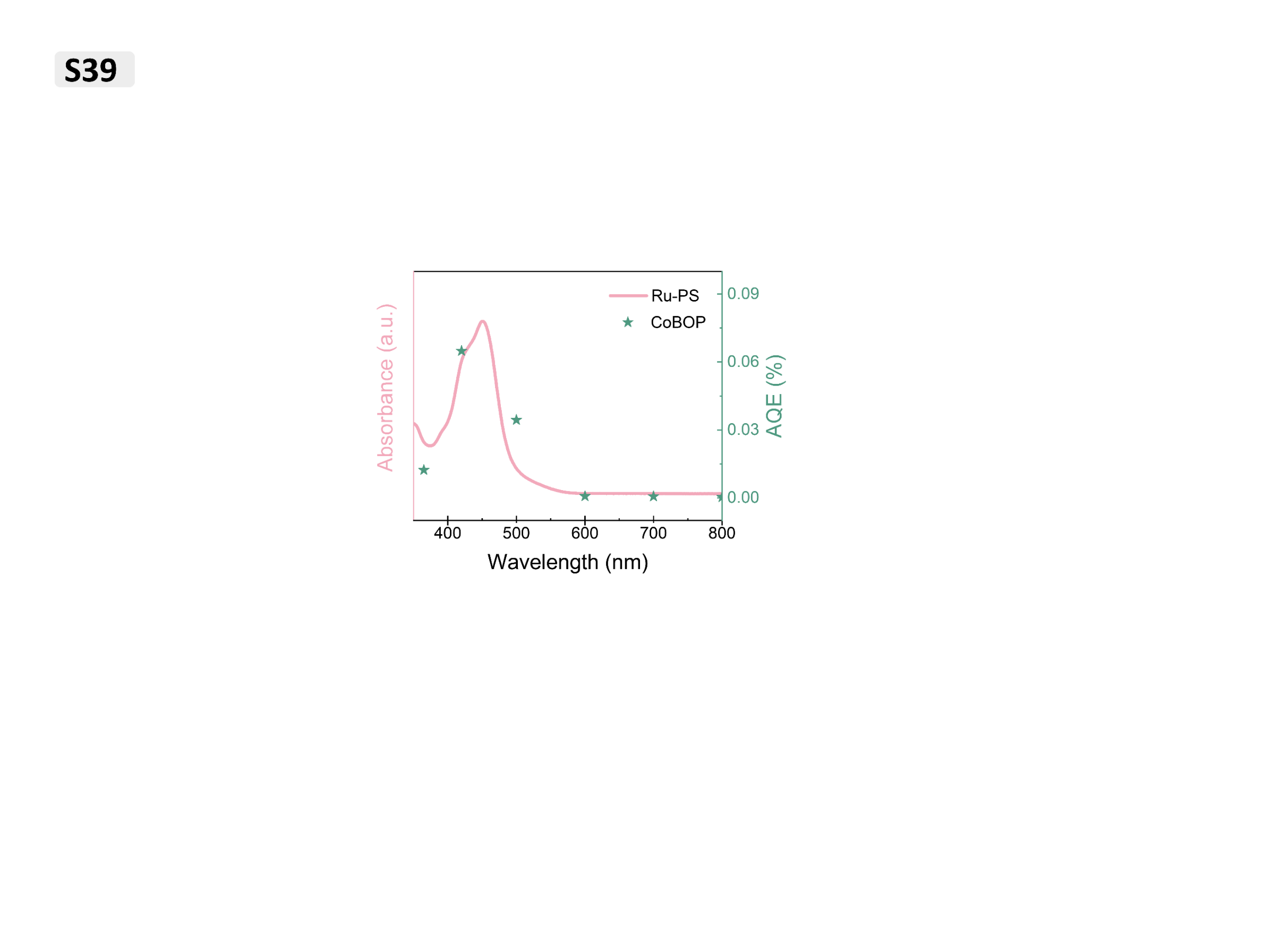


**Fig. S40** AQE of production catalyzed by CoBOP as a function of the related spectra


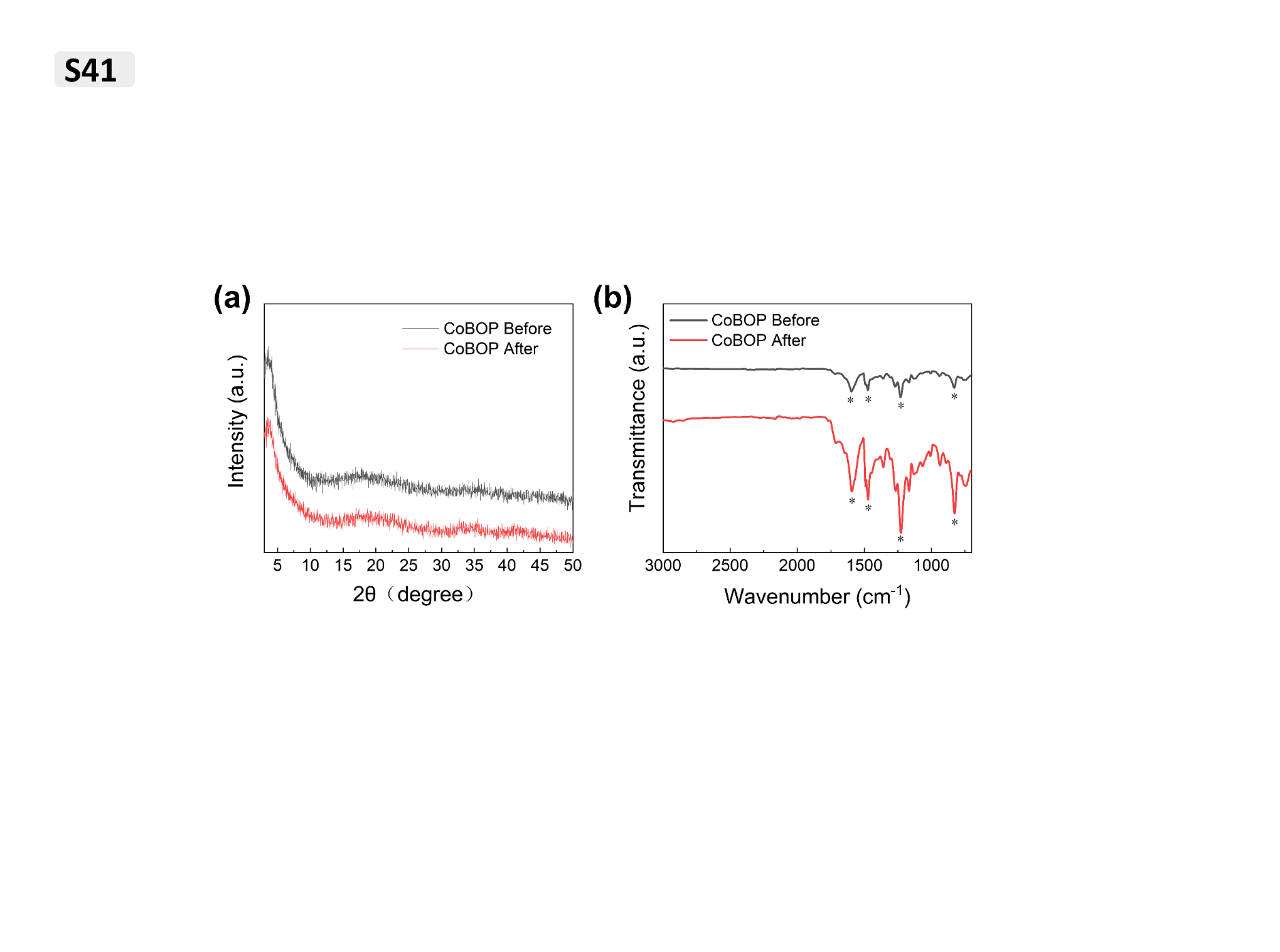


**Fig. S41** XRD and FT-IR spectra before and after photocatalytic reaction of CoBOP


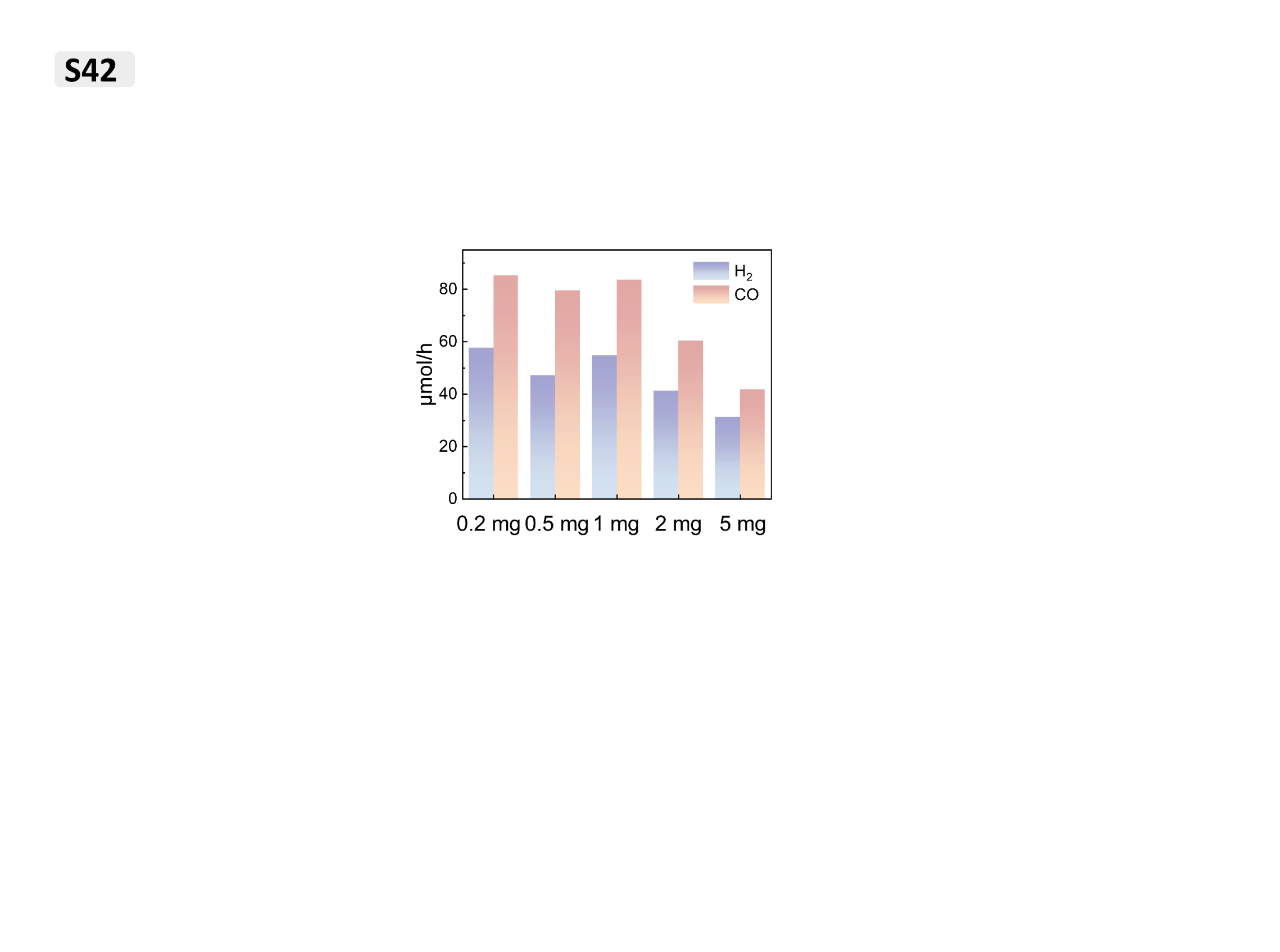


**Fig. S42**. Photocatalytic performance with different CoBOP dosages


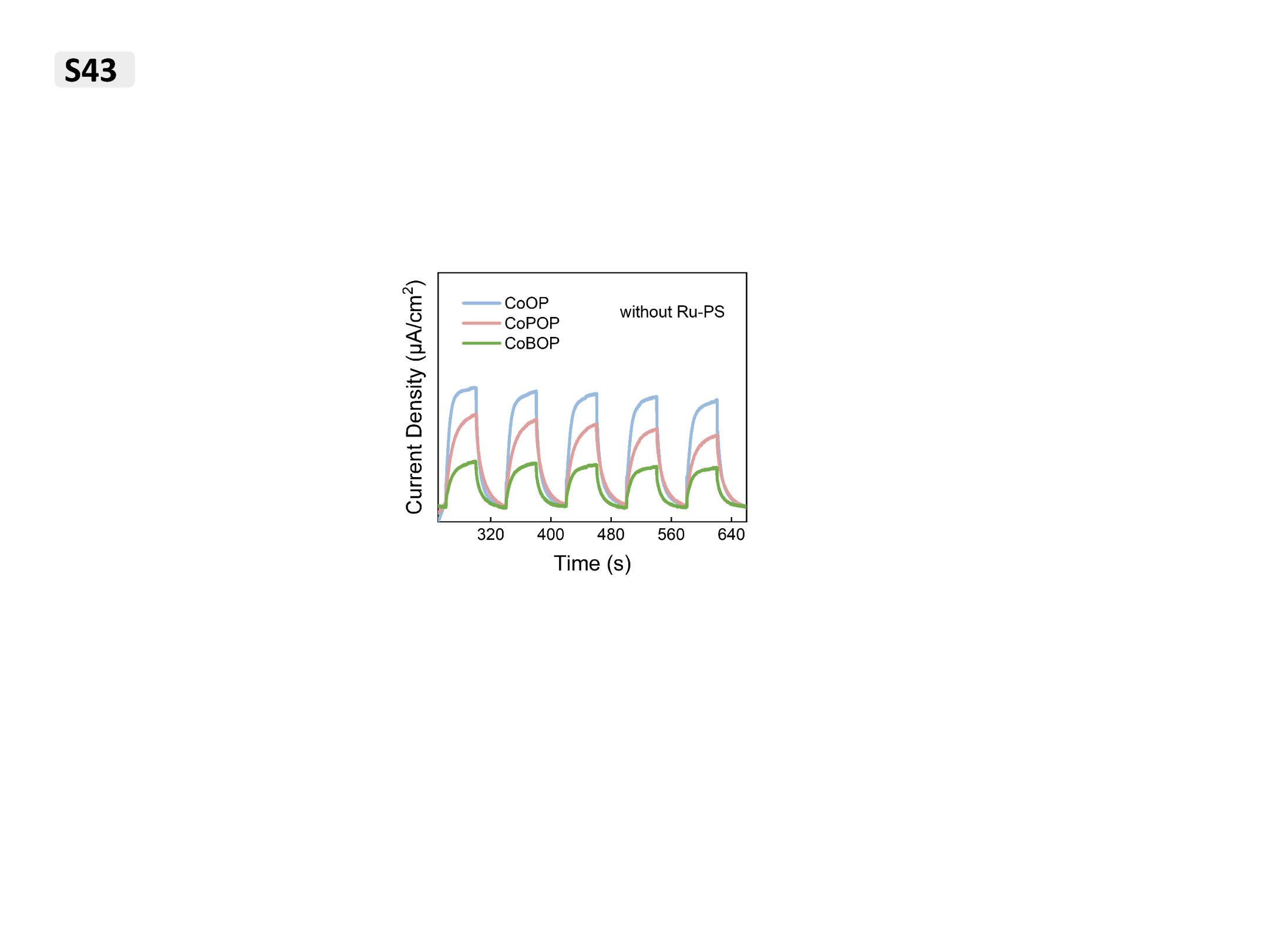


**Fig. S43** Transient photocurrent response of CoOP, CoPOP, and CoBOP at 0.2 M Na_2_SO_4_ electrolyte


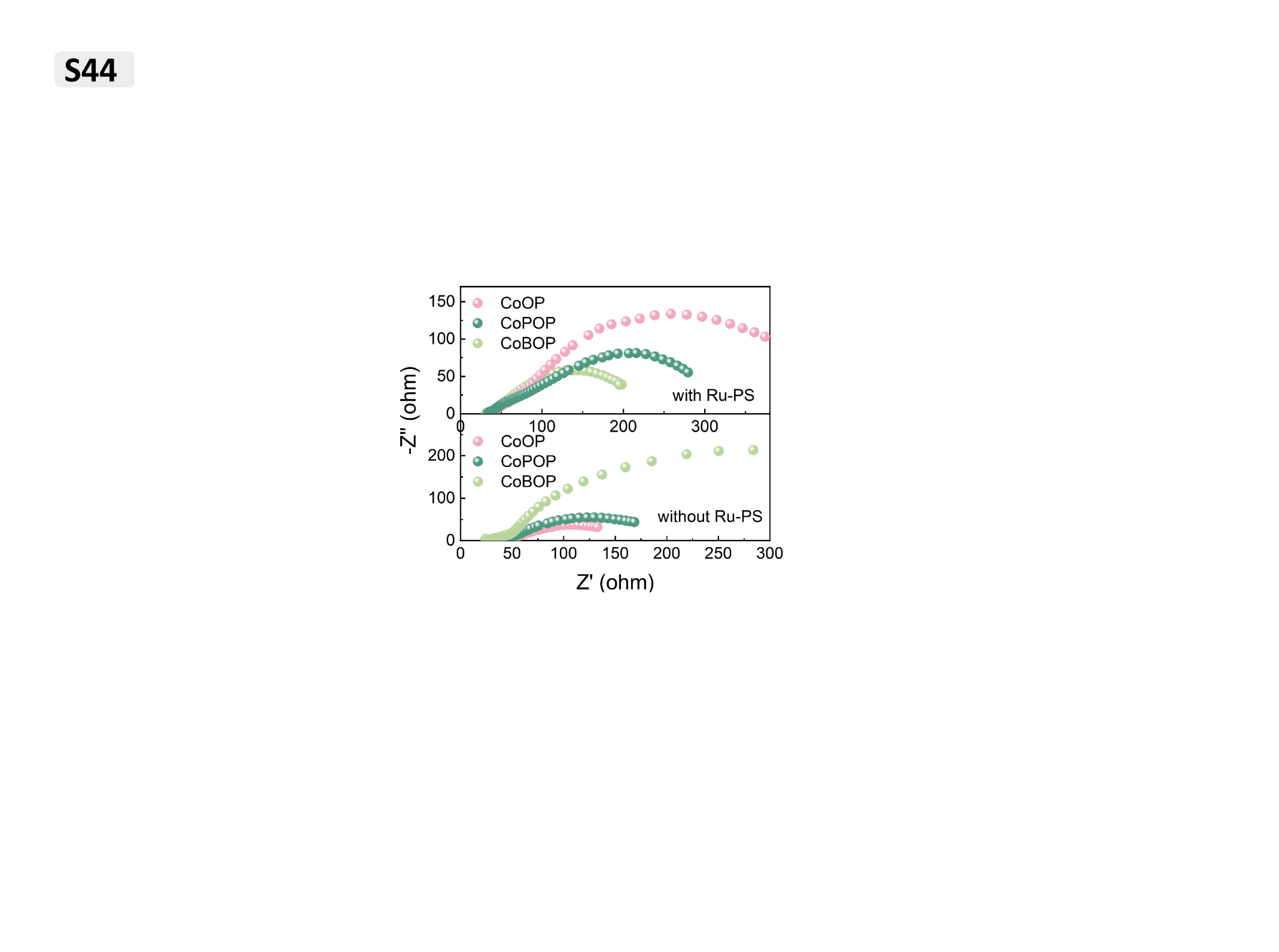


**Fig. S44** EIS plots of the CoOP, CoPOP, and CoBOP at 0.2 M Na_2_SO_4_ electrolyte


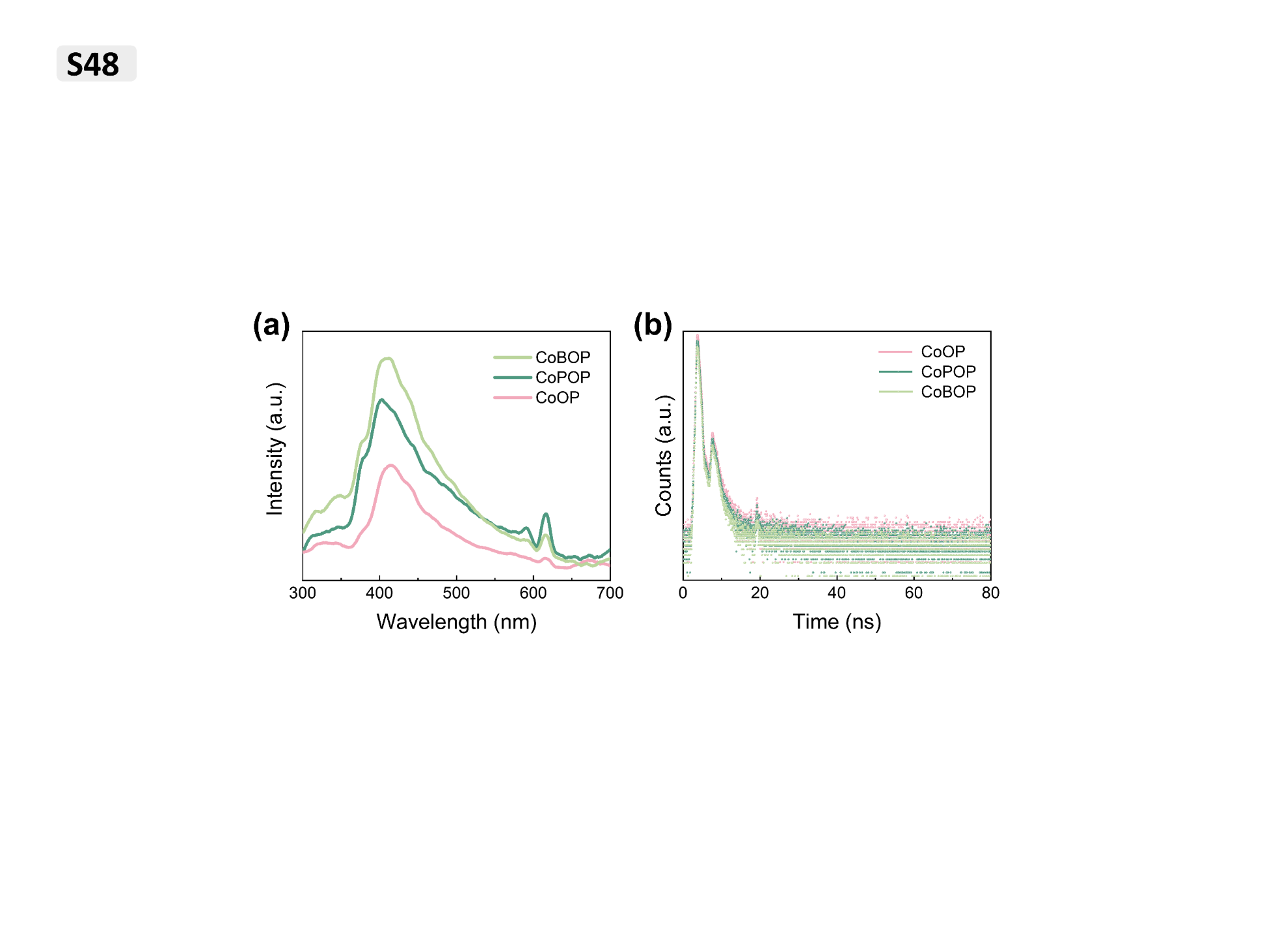


**Fig. S45** **a** Steady-state PL spectra of CoOP, CoPOP, and CoBOP. **b** Time-resolved PL decay spectra of CoOP, CoPOP, and CoBOP


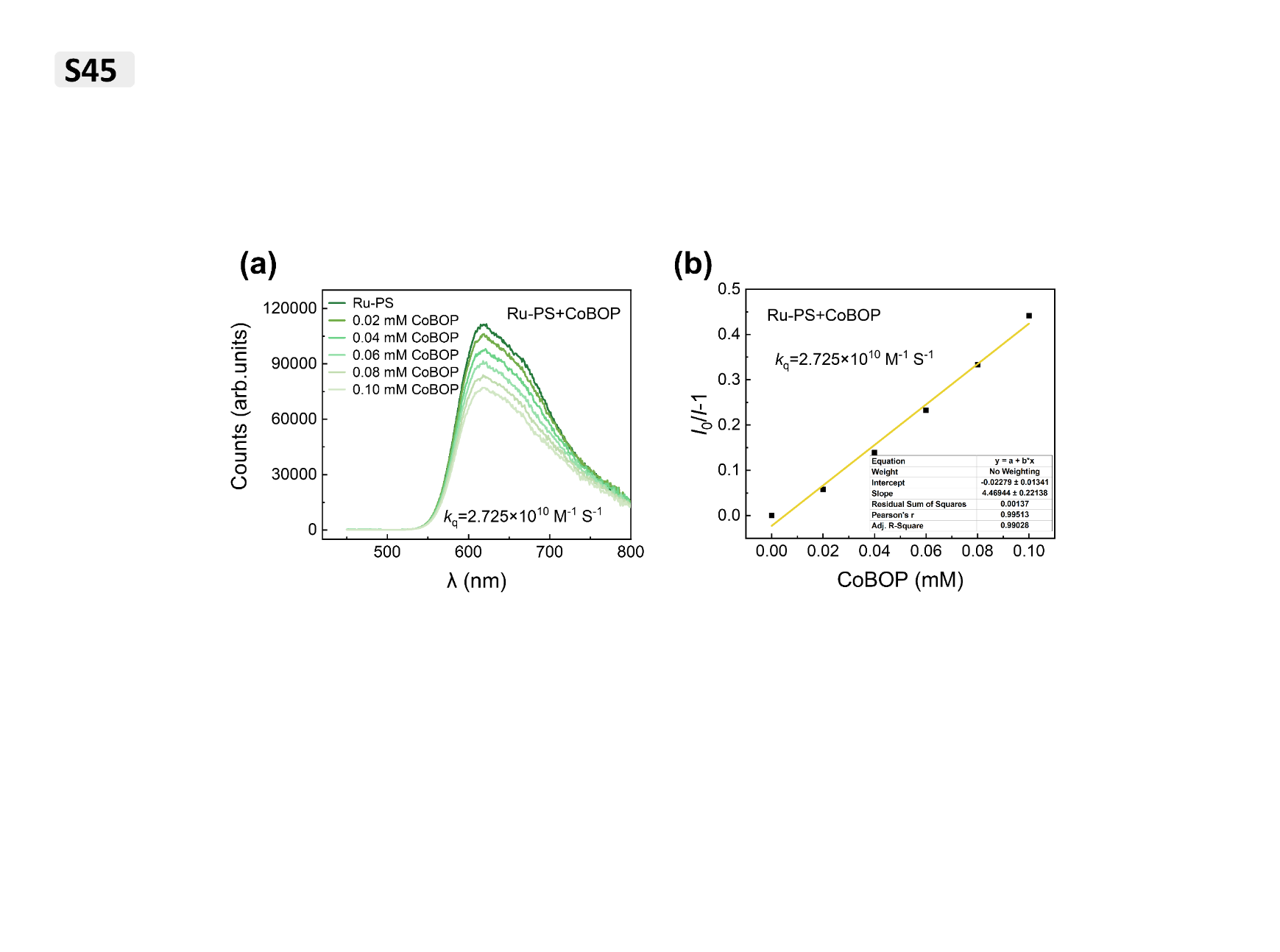


**Fig. S46** **a** Steady-state PL spectra of a CH_3_CN solution containing 0.05 mM Ru-PS in the presence of 0~0.10 mM CoBOP, respectively. **b** Linear fitting of the ratio of PL intensity versus CoBOP


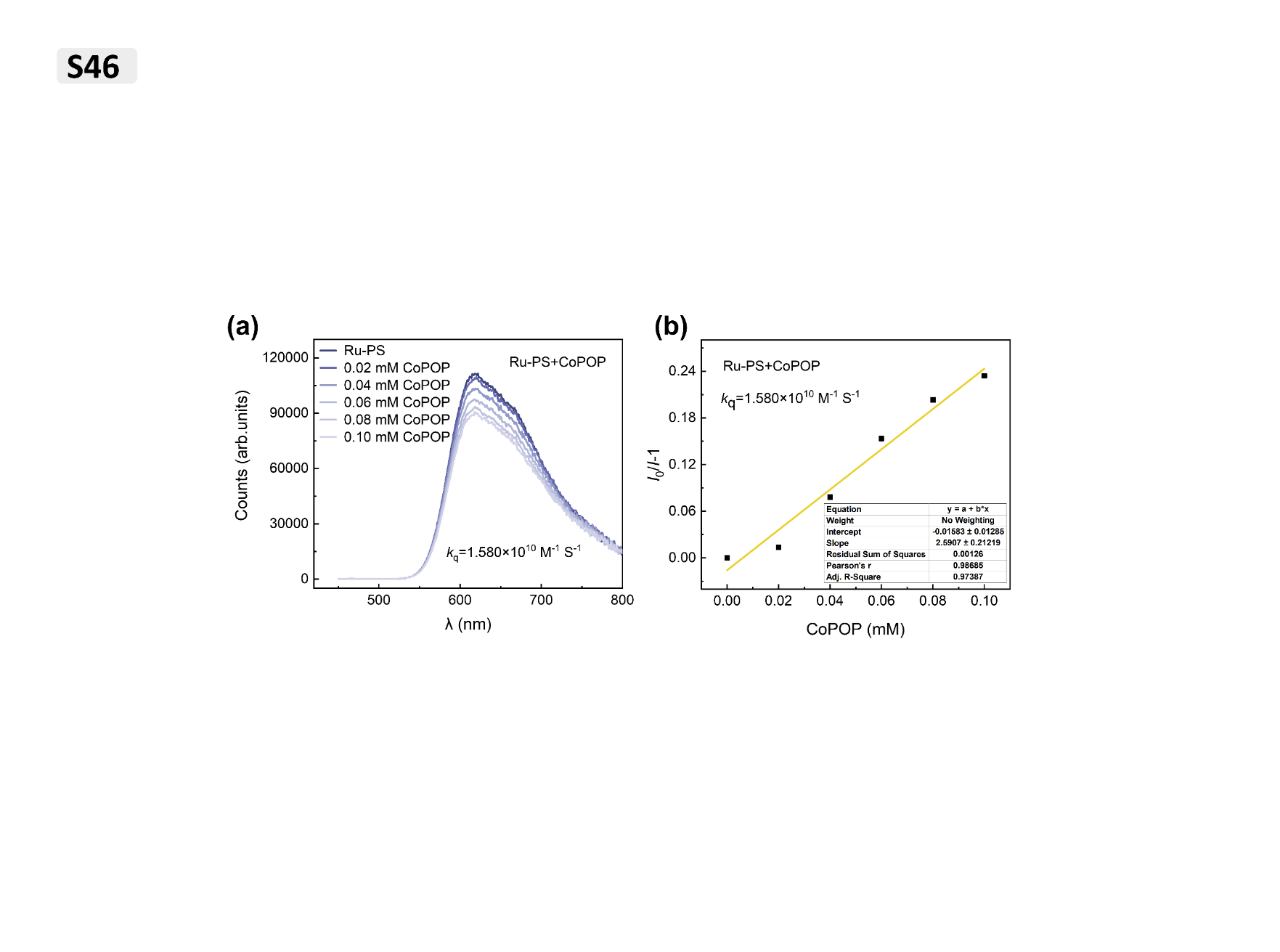


**Fig. S47** **a** Steady-state PL spectra of a CH_3_CN solution containing 0.05 mM Ru-PS in the presence of 0~0.10 mM CoPOP, respectively. **b** Linear fitting of the ratio of PL intensity versus CoPOP


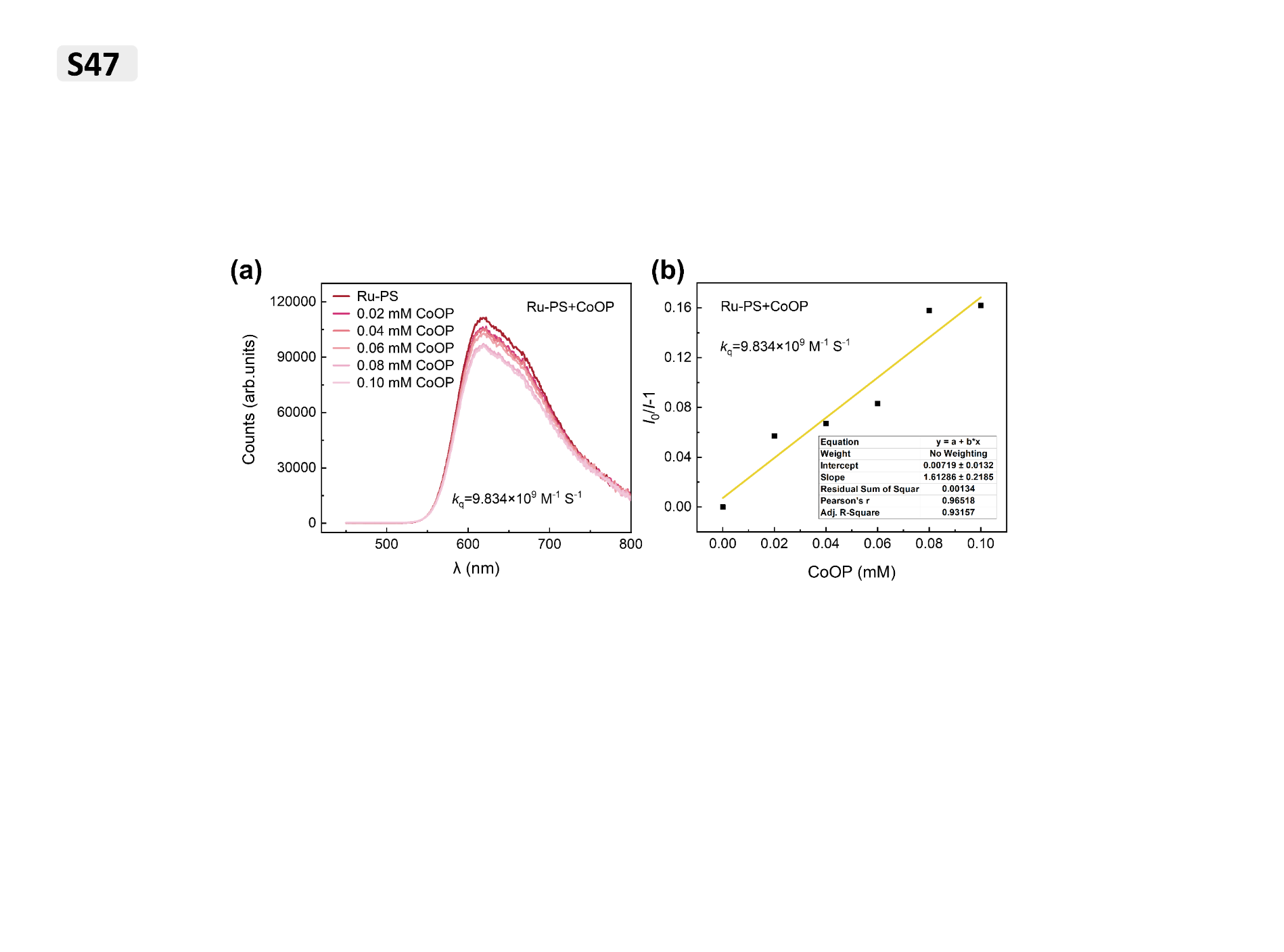


**Fig. S48** **a** Steady-state PL spectra of a CH_3_CN solution containing 0.05 mM Ru-PS in the presence of 0~0.10 mM CoOP, respectively. **b** Linear fitting of the ratio of PL intensity versus CoOP

**Table S1** The performance comparison of COF photocatalysts using photosensitizer and sacrificial agent for photocatalytic CO_2_-to-CO conversion

| **Photocatalyst** | **Photosensitizers** | **Sacrificial**  **Agent** | **solution**  **components** | **CO Yield**  **mmol g^-1^ h^-1^** | **H_2_ Yield**  **mmol g^-1^ h^-1^** | **CO Yield**  **μmol h^-1^** | **Refs.** |
| --- | --- | --- | --- | --- | --- | --- | --- |
| CoBOP  0.2 mg | [Ru(bpy)_3_]Cl_2_  1 mg/mL | TEOA | MeCN/H_2_O  20 mL | 426 | 288 | 85.2 | This  work |
| CoBOP  0.5 mg | [Ru(bpy)_3_]Cl_2_  1 mg/mL | TEOA | MeCN/H_2_O  20 mL | 147.2 | 94.4 | 73.6 | This  work |
| CoBOP  1 mg | [Ru(bpy)_3_]Cl_2_  1 mg/mL | TEOA | MeCN/H_2_O  20 mL | 83.7 | 54.7 | 83.7 | This  work |
| CoPOP  1 mg | [Ru(bpy)_3_]Cl_2_  1 mg/mL | TEOA | MeCN/H_2_O  20 mL | 68.8 | 46.2 | 68.8 | This  work |
| CoOP  1 mg | [Ru(bpy)_3_]Cl_2_  1 mg/mL | TEOA | MeCN/H_2_O  20 mL | 53.6 | 32.6 | 53.6 | This  work |
| TPy-COF-Co  1 mg | [Ru(bpy)_3_]Cl_2_  1.25 mg/mL | TEOA | MeCN/H_2_O  6 mL | 26.9 | 14.7 | 26.9 | [S3] |
| Co/Cu_3_-TPA-COF  1 mg | [Ru(bpy)_3_]Cl_2_  1 mg/mL | TEOA | MeCN/H_2_O  5 mL | 25.2 | 6.23 | 25.2 | [S4] |
| USTB-11(Cu,Ni)  2 mg | [Ru(bpy)_3_]Cl_2_  6 mg/mL | TEOA | MeCN/H_2_O  5 mL | 22.13 | 0.45 | 44.26 | [S5] |
| Co-2,3-DHTA-COF  1 mg | [SRu(bpy)_3_]Cl_2_  0.22 mg/mL | TEOA | MeCN/H_2_O  46 mL | 18.00 | 0.81 | 18 | [S6] |
| EPCo-COF-AT  2 mg | [SRu(bpy)_3_]Cl_2_  0.5 mg/mL | TEOA | MeCN/H_2_O  20 mL | 17.70 | 0.40 | 35.4 | [S7] |
| JUC-640-Co  3 mg | [Ru(bpy)_3_]Cl_2_  0.25 mg/mL | BIH | MeCN/H_2_O  20 mL | 15.14 | 0.90 | 45.42 | [S8] |
| TT-Por(Co)-COF  5 mg | [SRu(bpy)_3_]Cl_2_  6 mg/mL | TEOA | MeCN/H_2_O  5 mL | 10.50 | 8.94 | 52.5 | [S9] |
| CoPor-DPP-COF  2 mg | [SRu(bpy)_3_]Cl_2_  0.625 mg/mL | TIPA | MeCN/H_2_O  16 mL | 10.20 | 2.24 | 20.4 | [S10] |
| COF-367-Co NSs  5 mg | [SRu(bpy)_3_]Cl_2_  0.95 mg/mL | AA | 0.1 M KHCO3  20 mL | 10.16 | 3.00 | 50.8 | [S11] |
| Co-PyPor-COF  2 mg | [SRu(bpy)_3_]Cl_2_  1.25 mg/mL | TEOA | MeCN/H_2_O  8 mL | 9.60 | 0.33 | 19.2 | [S12] |
| CdS/TpBpy-20%  2 mg | Co(bpy)_3_^2+^ | TEOA | MeCN/H_2_O  15 mL | 8.80 | 1.71 | 17.6 | [S13] |
| Co-TAPB-COF-1  10 mg | [SRu(bpy)_3_]Cl_2_  0.6 mg/mL | TEOA | MeCN/H_2_O  100 mL | 8.39 | 11.31 | 83.9 | [S14] |
| Ni-COF  2 mg | [SRu(bpy)_3_]Cl_2_  1.25 mg/mL | TEOA | MeCN/H_2_O  6 mL | 5.31 | 0.29 | 10.62 | [S15] |
| Ni-TP-CON  5 mg | [SRu(bpy)_3_]Cl_2_  2 mg/mL | TEOA | MeCN/H_2_O  10 mL | 4.98 | 0.13 | 24.9 | [S16] |
| NiPc-CoPOP  2 mg | [SRu(bpy)_3_]Cl_2_  1 mg/mL | TEOA | MeCN/H_2_O  10 mL | 4.27 | 3.64 | 8.54 | [S17] |
| Ni@TPHH-COF  5 mg | [SRu(bpy)_3_]Cl_2_  1.17 mg/mL | TEOA | MeCN/H_2_O  6 mL | 3.28 | 0.17 | 16.4 | [S18] |
| HOF-25-Re  2 mg | [SRu(bpy)_3_]Cl_2_ | TIPA | MeCN | 3.03 | 0.26 | 6.06 | [S19] |
| CoNi-COF-3  1 mg | [SRu(bpy)_3_]Cl_2_  0.86 mg/mL | TEOA | MeCN/H_2_O  5 mL | 2.57 | 0.25 | 2.57 | [S20] |
| CoP-TPE-COF  15 mg | [SRu(bpy)_3_]Cl_2_  5 mg/mL | TEOA | MeCN/H_2_O  6 mL | 2.41 | 1.54 | 36.15 | [S21] |
| H-COF-Ni  2 mg | [SRu(bpy)_3_]Cl_2_  5 mg/mL | TEOA | MeCN/H_2_O  6 mL | 2.31 | 0.10 | 4.62 | [S22] |
| TFBD-COF-Co-SA  10 mg | [SRu(bpy)_3_]Cl_2_  0.6 mg/mL | TEOA | MeCN  50 mL | 1.48 | 0.16 | 14.8 | [S23] |
| Co@COF-TVBT-Bpy  2 mg | [SRu(bpy)_3_]Cl_2_  0.3 mg/mL | TEOA | MeCN/H_2_O  11.6 mL | 1.13 | 1.13 | 2.26 | [S24] |
| CH_3_-TPPD  5 mg | [SRu(bpy)_3_]Cl_2_  1.2 mg/mL | TEOA | MeCN/H_2_O  6 mL | 1.03 | 0.27 | 5.15 | [S25] |
| DQTP COF-Co  20 mg | [SRu(bpy)_3_]Cl_2_  0.45 mg/mL | TEOA | MeCN  50 mL | 1.02 | 0.11 | 20.4 | [S26] |
| CTF-Bpy-Co  10 mg | [SRu(bpy)_3_]Cl_2_  0.325 mg/mL | TEOA | MeCN/H_2_O  20 mL | 1.02 | 0.20 | 10.2 | [S27] |
| Fe-SAS/TrCOF  5 mg | [SRu(bpy)_3_]Cl_2_  2 mg/mL | TEOA | MeCN/H_2_O  5 mL | 0.98 | 0.04 | 4.9 | [S28] |
| Ni-TpBpy  10 mg | [SRu(bpy)_3_]Cl_2_  1.3 mg/mL | TEOA | MeCN/H_2_O  5 mL | 0.81 | 0.03 | 8.1 | [S29] |
| Ni-PCD@TD-COF  5 mg | [SRu(bpy)_3_]Cl_2_  2 mg/mL | TEOA | MeCN/H_2_O  10 mL | 0.48 | 0.01 | 2.4 | [S30] |
| α-Fe_2_O_3_@Por-CTF  20 mg | [SRu(bpy)_3_]Cl_2_  1 mg/mL | TEOA | MeCN/H_2_O  5 mL | 0.4 | 0.03 | 8 | [S31] |

**Table S2** Atomic coordinates for geometrically optimized CoOP using FORCITE calculation

| CoOP | Space group: P1  a=15.6 Å, b=15.4, Å c=3.5Å  α=94.8°, β=94.0°, γ= 89.3° | | | |
| --- | --- | --- | --- | --- |
| C1 | C | 1.23296 | -1.46239 | -0.60229 |
| C2 | C | 1.23226 | -1.54885 | -0.54671 |
| C3 | C | 1.32226 | -1.57577 | -0.51446 |
| N4 | N | 1.37489 | -1.50705 | -0.53806 |
| C5 | C | 1.32249 | -1.43747 | -0.59044 |
| C6 | C | 1.54195 | -1.2431 | -0.37551 |
| C7 | C | 1.45585 | -1.24216 | -0.47684 |
| C8 | C | 1.43058 | -1.32818 | -0.51958 |
| N9 | N | 1.49867 | -1.38201 | -0.45874 |
| C10 | C | 1.56663 | -1.32859 | -0.37082 |
| N11 | N | 1.34845 | -1.3543 | -0.58362 |
| C12 | C | 1.76732 | -1.54896 | -0.47702 |
| C13 | C | 1.76554 | -1.4623 | -0.36555 |
| C14 | C | 1.67576 | -1.43767 | -0.3613 |
| N15 | N | 1.62417 | -1.50733 | -0.45785 |
| C16 | C | 1.67759 | -1.57626 | -0.52508 |
| N17 | N | 1.64891 | -1.35458 | -0.32235 |
| C18 | C | 1.45624 | -1.77239 | -0.54813 |
| C19 | C | 1.5424 | -1.77242 | -0.59707 |
| C20 | C | 1.56761 | -1.68711 | -0.5847 |
| N21 | N | 1.49915 | -1.63324 | -0.53803 |
| C22 | C | 1.43087 | -1.68621 | -0.51975 |
| N23 | N | 1.34851 | -1.6592 | -0.5044 |
| C24 | N | 1.65198 | -1.66149 | -0.58614 |
| C25 | C | 1.84459 | -1.59355 | -0.53217 |
| C26 | C | 1.92207 | -1.54637 | -0.46891 |
| C27 | C | 1.91844 | -1.45672 | -0.3398 |
| C28 | C | 1.83991 | -1.41345 | -0.29125 |
| C29 | C | 1.58978 | -1.16755 | -0.30572 |
| C30 | C | 1.54621 | -1.08879 | -0.34941 |
| C31 | C | 1.45728 | -1.08574 | -0.46916 |
| C32 | C | 1.41085 | -1.16448 | -0.52614 |
| C33 | C | 1.41113 | -1.84951 | -0.52767 |
| C34 | C | 1.45739 | -1.92888 | -0.55796 |
| C35 | C | 1.54625 | -1.92702 | -0.6242 |
| C36 | C | 1.58993 | -1.84883 | -0.63992 |
| C37 | C | 1.15787 | -1.41395 | -0.64622 |
| C38 | C | 1.07995 | -1.45723 | -0.62654 |
| C39 | C | 1.07755 | -1.54648 | -0.55537 |
| C40 | C | 1.15567 | -1.59339 | -0.52146 |
| O41 | O | 1.4125 | -1.00702 | -0.52764 |
| O42 | O | 1.00011 | -1.59164 | -0.52719 |
| Co43 | Co | 1.49926 | -1.50751 | -0.4982 |
| H44 | H | 1.70161 | -1.71513 | -0.64155 |
| H45 | H | 1.84393 | -1.66611 | -0.62579 |
| H46 | H | 1.98043 | -1.41821 | -0.27297 |
| H47 | H | 1.83902 | -1.34108 | -0.19443 |
| H48 | H | 1.66141 | -1.17137 | -0.21697 |
| H49 | H | 1.58208 | -1.0246 | -0.28843 |
| H50 | H | 1.33885 | -1.16338 | -0.60976 |
| H51 | H | 1.33914 | -1.84716 | -0.48764 |
| H52 | H | 1.58399 | -1.99046 | -0.66635 |
| H53 | H | 1.66164 | -1.84971 | -0.68631 |
| H54 | H | 1.16128 | -1.34203 | -0.69595 |
| H55 | H | 1.01634 | -1.4208 | -0.66805 |
| H56 | H | 1.15502 | -1.66562 | -0.47482 |

**Table S3** Atomic coordinates for geometrically optimized CoPOP using FORCITE calculation

| CoPOP | Space group: P1  a=19.9 Å, b=20.4, Å c=4.3Å  α=66.8°, β=72.0°, γ= 90.0° | | | |
| --- | --- | --- | --- | --- |
| C1 | C | 0.45699 | -0.70028 | -0.5 |
| C2 | C | 0.52173 | -0.70778 | -0.5 |
| C3 | C | 0.54101 | -0.6454 | -0.5 |
| N4 | N | 0.4903 | -0.60115 | -0.5 |
| C5 | C | 0.4388 | -0.63309 | -0.5 |
| C6 | C | 0.29872 | -0.4562 | -0.5 |
| C7 | C | 0.29772 | -0.5197 | -0.5 |
| C8 | C | 0.36011 | -0.54544 | -0.5 |
| N9 | N | 0.39996 | -0.50067 | -0.5 |
| C10 | C | 0.36146 | -0.44605 | -0.5 |
| N11 | N | 0.37839 | -0.60696 | -0.5 |
| C12 | C | 0.52549 | -0.32321 | -0.5 |
| C13 | C | 0.46074 | -0.31572 | -0.5 |
| C14 | C | 0.44146 | -0.3781 | -0.5 |
| N15 | N | 0.49218 | -0.42234 | -0.5 |
| C16 | C | 0.54368 | -0.39039 | -0.5 |
| N17 | N | 0.38088 | -0.38911 | -0.5 |
| C18 | C | 0.68374 | -0.5673 | -0.5 |
| C19 | C | 0.68475 | -0.50379 | -0.5 |
| C20 | C | 0.62237 | -0.47804 | -0.5 |
| N21 | N | 0.58252 | -0.52282 | -0.5 |
| C22 | C | 0.62101 | -0.57745 | -0.5 |
| N23 | N | 0.60158 | -0.6344 | -0.5 |
| N24 | N | 0.60409 | -0.41652 | -0.5 |
| C25 | C | 0.55995 | -0.27354 | -0.5 |
| C26 | C | 0.52533 | -0.21452 | -0.5 |
| C27 | C | 0.45826 | -0.20555 | -0.5 |
| C28 | C | 0.42521 | -0.25778 | -0.5 |
| C29 | C | 0.24374 | -0.41431 | -0.5 |
| C30 | C | 0.18598 | -0.44023 | -0.5 |
| C31 | C | 0.18411 | -0.50722 | -0.5 |
| C32 | C | 0.24149 | -0.54682 | -0.5 |
| C33 | C | 0.7387 | -0.60921 | -0.5 |
| C34 | C | 0.79646 | -0.5833 | -0.5 |
| C35 | C | 0.79834 | -0.51629 | -0.5 |
| C36 | C | 0.74098 | -0.47668 | -0.5 |
| C37 | C | 0.42253 | -0.74995 | -0.5 |
| C38 | C | 0.45715 | -0.80898 | -0.5 |
| C39 | C | 0.52421 | -0.81796 | -0.5 |
| C40 | C | 0.55725 | -0.76573 | -0.5 |
| O41 | O | 0.42001 | -0.14974 | -0.5 |
| C42 | C | 0.45795 | -0.08219 | -0.5 |
| O43 | O | 0.12939 | -0.53301 | -0.5 |
| O44 | O | 0.85307 | -0.4905 | -0.5 |
| C45 | C | 0.92125 | -0.50229 | -0.5 |
| O46 | O | 0.56245 | -0.87376 | -0.5 |
| Co47 | Co | 0.49123 | -0.51175 | -0.5 |
| C48 | C | 0.52203 | 0.94651 | 0.5 |
| C49 | C | 0.55533 | 1.01602 | 0.5 |
| C50 | C | 0.52451 | 1.05869 | 0.5 |
| C51 | C | 0.46044 | 1.02999 | 0.5 |
| C52 | C | 0.42714 | 0.96047 | 0.5 |
| C53 | C | 0.95928 | 1.54312 | 0.5 |
| C54 | C | 1.02908 | 1.53348 | 0.5 |
| C55 | C | 1.06119 | 1.47878 | 0.5 |
| C56 | C | 1.02317 | 1.43336 | 0.5 |
| C57 | C | 0.95337 | 1.443 | 0.5 |
| H58 | H | 0.6106 | -0.28137 | -0.5 |
| H59 | H | 0.54945 | -0.17717 | -0.5 |
| H60 | H | 0.37344 | -0.25285 | -0.5 |
| H61 | H | 0.24562 | -0.36354 | -0.5 |
| H62 | H | 0.1436 | -0.40783 | -0.5 |
| H63 | H | 0.24173 | -0.59669 | -0.5 |
| H64 | H | 0.73681 | -0.65999 | -0.5 |
| H65 | H | 0.83883 | -0.6157 | -0.5 |
| H66 | H | 0.74074 | -0.4268 | -0.5 |
| H67 | H | 0.37189 | -0.74211 | -0.5 |
| H68 | H | 0.43304 | -0.84632 | -0.5 |
| H69 | H | 0.60902 | -0.77066 | -0.5 |
| H70 | H | 0.5452 | 0.91608 | 0.5 |
| H71 | H | 0.60464 | 1.03803 | 0.5 |
| H72 | H | 0.43726 | 1.06042 | 0.5 |
| H73 | H | 0.37782 | 0.93847 | 0.5 |
| H74 | H | 0.93409 | 1.58525 | 0.5 |
| H75 | H | 1.05816 | 1.56793 | 0.5 |
| H76 | H | 1.04835 | 1.39122 | 0.5 |
| H77 | H | 0.92429 | 1.40855 | 0.5 |

**Table S4** Atomic coordinates for geometrically optimized CoPOP using FORCITE calculation

| CoBOP | Space group: P1  a=24.7 Å, b=24.0 Å, c=4.0 Å  α=72.1°, β=95.6°, γ= 93.6° | | | |
| --- | --- | --- | --- | --- |
| C1 | C | 0.33296 | -0.48337 | -0.5 |
| C2 | C | 0.3297 | -0.53511 | -0.5 |
| C3 | C | 0.38502 | -0.54695 | -0.5 |
| N4 | N | 0.42079 | -0.50469 | -0.5 |
| C5 | C | 0.39024 | -0.46554 | -0.5 |
| C6 | C | 0.53654 | -0.34702 | -0.5 |
| C7 | C | 0.48181 | -0.35047 | -0.5 |
| C8 | C | 0.4628 | -0.40116 | -0.5 |
| N9 | N | 0.50405 | -0.42989 | -0.5 |
| C10 | C | 0.54922 | -0.39606 | -0.5 |
| N11 | N | 0.40987 | -0.41776 | -0.5 |
| C12 | C | 0.66581 | -0.5208 | -0.5 |
| C13 | C | 0.66889 | -0.46886 | -0.5 |
| C14 | C | 0.61342 | -0.4562 | -0.5 |
| N15 | N | 0.57777 | -0.49899 | -0.5 |
| C16 | C | 0.60852 | -0.53886 | -0.5 |
| N17 | N | 0.60002 | -0.40823 | -0.5 |
| C18 | C | 0.46192 | -0.65497 | -0.5 |
| C19 | C | 0.51729 | -0.65482 | -0.5 |
| C20 | C | 0.53619 | -0.60463 | -0.5 |
| N21 | N | 0.49451 | -0.57386 | -0.5 |
| C22 | C | 0.44898 | -0.60561 | -0.5 |
| N23 | N | 0.39811 | -0.59341 | -0.5 |
| N24 | N | 0.58915 | -0.5882 | -0.5 |
| C25 | C | 0.7113 | -0.54453 | -0.5 |
| C26 | C | 0.76155 | -0.51372 | -0.5 |
| C27 | C | 0.76589 | -0.4613 | -0.5 |
| C28 | C | 0.71812 | -0.43797 | -0.5 |
| C29 | C | 0.56888 | -0.29992 | -0.5 |
| C30 | C | 0.54339 | -0.25538 | -0.5 |
| C31 | C | 0.48625 | -0.25896 | -0.5 |
| C32 | C | 0.45519 | -0.30756 | -0.5 |
| C33 | C | 0.42959 | -0.69905 | -0.5 |
| C34 | C | 0.45619 | -0.74447 | -0.5 |
| C35 | C | 0.51395 | -0.74727 | -0.5 |
| C36 | C | 0.54482 | -0.70026 | -0.5 |
| C37 | C | 0.28768 | -0.45961 | -0.5 |
| C38 | C | 0.23771 | -0.49114 | -0.5 |
| C39 | C | 0.23377 | -0.54568 | -0.5 |
| C40 | C | 0.28099 | -0.56786 | -0.5 |
| O41 | O | 0.45958 | -0.2187 | -0.5 |
| O42 | O | 0.81477 | -0.42799 | -0.5 |
| O43 | O | 0.54265 | -0.79158 | -0.5 |
| O44 | O | 0.18618 | -0.58203 | -0.5 |
| C45 | C | 0.47426 | -0.15915 | -0.5 |
| C46 | C | 0.86617 | -0.45243 | -0.5 |
| C47 | C | 0.5295 | -0.85167 | -0.5 |
| C48 | C | 0.13532 | -0.55795 | -0.5 |
| C49 | C | 0.52117 | -0.13452 | -0.5 |
| C50 | C | 0.53343 | -0.07442 | -0.5 |
| C51 | C | 0.49697 | -0.03753 | -0.5 |
| C52 | C | 0.44864 | -0.06169 | -0.5 |
| C53 | C | 0.4376 | -0.12219 | -0.5 |
| C54 | C | 0.87742 | -0.51112 | -0.5 |
| C55 | C | 0.92942 | -0.53235 | -0.5 |
| C56 | C | 0.97281 | -0.49455 | -0.5 |
| C57 | C | 0.96188 | -0.43467 | -0.5 |
| C58 | C | 0.90926 | -0.41436 | -0.5 |
| C59 | C | 0.48524 | -0.87387 | -0.5 |
| C60 | C | 0.4741 | -0.93436 | -0.5 |
| C61 | C | 0.50884 | -0.97383 | -0.5 |
| C62 | C | 0.55502 | -0.95215 | -0.5 |
| C63 | C | 0.56452 | -0.8917 | -0.5 |
| C64 | C | 0.11935 | -0.50512 | -0.5 |
| C65 | C | 0.06694 | -0.48501 | -0.5 |
| C66 | C | 0.02886 | -0.51692 | -0.5 |
| C67 | C | 0.04526 | -0.57048 | -0.5 |
| C68 | C | 0.09739 | -0.59102 | -0.5 |
| Co69 | Co | 0.49927 | -0.50185 | -0.5 |
| H70 | H | 0.70783 | -0.58439 | -0.5 |
| H71 | H | 0.79616 | -0.53024 | -0.5 |
| H72 | H | 0.71959 | -0.39697 | -0.5 |
| H73 | H | 0.61231 | -0.29759 | -0.5 |
| H74 | H | 0.56889 | -0.21957 | -0.5 |
| H75 | H | 0.41183 | -0.31171 | -0.5 |
| H76 | H | 0.3856 | -0.69762 | -0.5 |
| H77 | H | 0.43202 | -0.77708 | -0.5 |
| H78 | H | 0.58891 | -0.69974 | -0.5 |
| H79 | H | 0.29115 | -0.41914 | -0.5 |
| H80 | H | 0.20309 | -0.47379 | -0.5 |
| H81 | H | 0.27974 | -0.60989 | -0.5 |
| H82 | H | 0.54801 | -0.16159 | -0.5 |
| H83 | H | 0.57053 | -0.05648 | -0.5 |
| H84 | H | 0.4199 | -0.0338 | -0.5 |
| H85 | H | 0.40028 | -0.14044 | -0.5 |
| H86 | H | 0.84622 | -0.54105 | -0.5 |
| H87 | H | 0.93472 | -0.57865 | -0.5 |
| H88 | H | 0.99369 | -0.40265 | -0.5 |
| H89 | H | 0.90195 | -0.36833 | -0.5 |
| H90 | H | 0.45948 | -0.84589 | -0.5 |
| H91 | H | 0.43927 | -0.95052 | -0.5 |
| H92 | H | 0.5829 | -0.98191 | -0.5 |
| H93 | H | 0.59945 | -0.87571 | -0.5 |
| H94 | H | 0.14691 | -0.48003 | -0.5 |
| H95 | H | 0.05632 | -0.4453 | -0.5 |
| H96 | H | 0.01885 | -0.59642 | -0.5 |
| H97 | H | 0.1088 | -0.63243 | -0.5 |

**Supplementary References**

1. V.S. Thoi, N. Kornienko, C.G. Margarit, P. Yang, C.J. Chang, Visible-light photoredox catalysis: selective reduction of carbon dioxide to carbon monoxide by a nickel N-heterocyclic carbene–isoquinoline complex. J. Am. Chem. Soc. **135**(38), 14413–14424 (2013). <https://doi.org/10.1021/ja4074003>
2. M. J. Frisch and g. W. Trucks and h. B. Schlegel, gaussian 16, revision a.03, gaussian inc. Wallingford ct. (2016).
3. P. Fu, C. Chen, C. Wu, B. Meng, Q. Yue et al., Covalent organic framework stabilized single CoN(4)Cl(2) site boosts photocatalytic CO_2_ reduction into tunable syngas. Angew. Chem. Int. Ed. **64**(3), e202415202 (2025). <https://doi.org/10.1002/anie.202415202>
4. X. Lan, H. Li, Y. Liu, Y. Zhang, T. Zhang et al., Covalent organic framework with Donor1-acceptor-Donor2 motifs regulating local charge of intercalated single cobalt sites for photocatalytic CO_2_ reduction to syngas. Angew. Chem. Int. Ed. **63**(31), e202407092 (2024). <https://doi.org/10.1002/anie.202407092>
5. X. Wang, X. Ding, Y. Jin, D. Qi, H. Wang et al., Post-nickelation of a crystalline trinuclear copper organic framework for synergistic photocatalytic carbon dioxide conversion. Angew. Chem. Int. Ed. **62**(18), e202302808 (2023). <https://doi.org/10.1002/anie.202302808>
6. Q. Zhang, S. Gao, Y. Guo, H. Wang, J. Wei et al., Designing covalent organic frameworks with Co-O(4) atomic sites for efficient CO_2_ photoreduction. Nat. Commun. **14**(1), 1147 (2023). <https://doi.org/10.1038/s41467-023-36779-4>
7. W. Lin, F. Lin, J. Lin, Z. Xiao, D. Yuan et al., Efficient photocatalytic CO_2_ reduction in ellagic acid–based covalent organic frameworks. J. Am. Chem. Soc. **146**(23), 16229–16236 (2024). <https://doi.org/10.1021/jacs.4c04185>
8. J. Ding, X. Guan, J. Lv, X. Chen, Y. Zhang et al., Three-dimensional covalent organic frameworks with ultra-large pores for highly efficient photocatalysis. J. Am. Chem. Soc. **145**(5), 3248–3254 (2023). <https://doi.org/10.1021/jacs.2c13817>
9. S. Zhao, J. Liang, D.-H. Si, M.-J. Mao, Y. Huang et al., Superheterojunction covalent organic frameworks: Supramolecular synergetic charge transfer for highly efficient photocatalytic CO_2_ reduction. Appl. Catal. B Environ. Energy **333**, 122782 (2023). <https://doi.org/10.1016/j.apcatb.2023.122782>
10. X. Wang, X. Ding, T. Wang, K. Wang, Y. Jin et al., Two-dimensional porphyrin-based covalent organic framework with enlarged inter-layer spacing for tunable photocatalytic CO_2_ reduction. ACS Appl. Mater. Interfaces **14**(36), 41122–41130 (2022). <https://doi.org/10.1021/acsami.2c12542>
11. W. Liu, X. Li, C. Wang, H. Pan, W. Liu et al., A scalable general synthetic approach toward ultrathin imine-linked two-dimensional covalent organic framework nanosheets for photocatalytic CO_2_ reduction. J. Am. Chem. Soc. **141**(43), 17431–17440 (2019). <https://doi.org/10.1021/jacs.9b09502>
12. T.-X. Luan, J.-R. Wang, K. Li, H. Li, F. Nan et al., Highly enhancing CO_2_ photoreduction by metallization of an imidazole-linked robust covalent organic framework. Small **19**(44), 2303324 (2023). <https://doi.org/10.1002/smll.202303324>
13. K.H. Do, D.P. Kumar, A.P. Rangappa, J. Lee, S. Yun et al., Design and synthesis of a covalent organic framework bridging CdS nanoparticles and a homogeneous cobalt–bipyridine cocatalyst for a highly efficient photocatalytic CO_2_ reduction. J. Mater. Chem. A **11**(15), 8392–8403 (2023). <https://doi.org/10.1039/D3TA00079F>
14. W. Zhou, X. Wang, W. Zhao, N. Lu, D. Cong et al., Photocatalytic CO_2_ reduction to syngas using metallosalen covalent organic frameworks. Nat. Commun. **14**(1), 6971 (2023). <https://doi.org/10.1038/s41467-023-42757-7>
15. B. Han, X. Ou, Z. Zhong, S. Liang, H. Deng et al., Rational design of FeNi bimetal modified covalent organic frameworks for photoconversion of anthropogenic CO_2_ into widely tunable syngas. Small **16**(38), 2002985 (2020). <https://doi.org/10.1002/smll.202002985>
16. H. Lv, P. Li, X. Li, A. Chen, R. Sa et al., Boosting photocatalytic reduction of the diluted CO_2_ over covalent organic framework. Chem. Eng. J. **451**, 138745 (2023). <https://doi.org/10.1016/j.cej.2022.138745>
17. X.-Y. Dong, Y.-N. Si, Q.-Y. Wang, S. Wang, S.-Q. Zang, Integrating single atoms with different microenvironments into one porous organic polymer for efficient photocatalytic CO_2_ reduction. Adv. Mater. **33**(33), 2101568 (2021). <https://doi.org/10.1002/adma.202101568>
18. M. Dong, J. Zhou, J. Zhong, H.-T. Li, C.-Y. Sun et al., CO_2_ dominated bifunctional catalytic sites for efficient industrial exhaust conversion. Adv. Funct. Mater. **32**(8), 2110136 (2022). <https://doi.org/10.1002/adfm.202110136>
19. B. Yu, L. Li, S. Liu, H. Wang, H. Liu et al., Robust biological hydrogen-bonded organic framework with post-functionalized rhenium(I) sites for efficient heterogeneous visible-light-driven CO_2_ reduction. Angew. Chem. Int. Ed. **60**(16), 8983–8989 (2021). <https://doi.org/10.1002/anie.202016710>
20. J. Wang, W. Zhu, F. Meng, G. Bai, Q. Zhang et al., Integrating dual-metal sites into covalent organic frameworks for enhanced photocatalytic CO_2_ reduction. ACS Catal. **13**(7), 4316–4329 (2023). <https://doi.org/10.1021/acscatal.3c00126>
21. H. Lv, R. Sa, P. Li, D. Yuan, X. Wang et al., Metalloporphyrin-based covalent organic frameworks composed of the electron donor-acceptor dyads for visible-light-driven selective CO_2_ reduction. Sci. China Chem. **63**(9), 1289–1294 (2020). <https://doi.org/10.1007/s11426-020-9801-3>
22. S. Yang, R. Sa, H. Zhong, H. Lv, D. Yuan et al., Microenvironments enabled by covalent organic framework linkages for modulating active metal species in photocatalytic CO_2_ reduction. Adv. Funct. Mater. **32**(17), 2110694 (2022). <https://doi.org/10.1002/adfm.202110694>
23. Y. Yang, Y. Lu, H.-Y. Zhang, Y. Wang, H.-L. Tang et al., Decoration of active sites in covalent–organic framework: an effective strategy of building efficient photocatalysis for CO_2_ reduction. ACS Sustainable Chem. Eng. **9**(39), 13376–13384 (2021). <https://doi.org/10.1021/acssuschemeng.1c04994>
24. J.-X. Cui, Y.-M. Fu, B. Meng, J. Zhou, Z.-Y. Zhou et al., A novel cobalt-anchored covalent organic framework for photocatalytic conversion of CO_2_ into widely adjustable syngas. J. Mater. Chem. A **10**(25), 13418–13427 (2022). <https://doi.org/10.1039/D2TA02648A>
25. M. Dong, W. Li, J. Zhou, S.-Q. You, C.-Y. Sun et al., Microenvironment modulation of imine-based covalent organic frameworks for CO_2_ photoreduction. Chin. J. Chem. **40**(22), 2678–2684 (2022). <https://doi.org/10.1002/cjoc.202200046>
26. M. Lu, Q. Li, J. Liu, F.-M. Zhang, L. Zhang et al., Installing earth-abundant metal active centers to covalent organic frameworks for efficient heterogeneous photocatalytic CO_2_ reduction. Appl. Catal. B Environ. **254**, 624–633 (2019). <https://doi.org/10.1016/j.apcatb.2019.05.033>
27. X. Hu, L. Zheng, S. Wang, X. Wang, B. Tan, Integrating single Co sites into crystalline covalent triazine frameworks for photoreduction of CO_2_. Chem. Commun. **58**(58), 8121–8124 (2022). <https://doi.org/10.1039/D2CC02481K>
28. L. Ran, Z. Li, B. Ran, J. Cao, Y. Zhao et al., Engineering single-atom active sites on covalent organic frameworks for boosting CO_2_ photoreduction. J. Am. Chem. Soc. **144**(37), 17097–17109 (2022). <https://doi.org/10.1021/jacs.2c06920>
29. W. Zhong, R. Sa, L. Li, Y. He, L. Li et al., A covalent organic framework bearing single Ni sites as a synergistic photocatalyst for selective photoreduction of CO_2_ to CO. J. Am. Chem. Soc. **141**(18), 7615–7621 (2019). <https://doi.org/10.1021/jacs.9b02997>
30. H. Zhong, R. Sa, H. Lv, S. Yang, D. Yuan et al., Covalent organic framework hosting metalloporphyrin-based carbon dots for visible-light-driven selective CO_2_ reduction. Adv. Funct. Mater. **30**(35), 2002654 (2020). <https://doi.org/10.1002/adfm.202002654>
31. S. Zhang, S. Wang, L. Guo, H. Chen, B. Tan et al., An artificial photosynthesis system comprising a covalent triazine framework as an electron relay facilitator for photochemical carbon dioxide reduction. J. Mater. Chem. C **8**(1), 192–200 (2020). <https://doi.org/10.1039/C9TC05297F>
